# Supplementary material for: Grey wolf genomic history reveals a dual ancestry of dogs
Source: Nature. 2022 Jun 29;607(7918):313–20. doi: 10.1038/s41586-022-04824-9 (PMC9279150; doi:10.1038/s41586-022-04824-9)
Supplement: Supplementary file 1 — This file contains the following five sections: section 1: mitochondrial phylogeny and dating; section 2: wolf population history analyses; section 3: MSMC2 analyses of Ne history and divergence times; section 4: natural selection analyses; section 5: dog ancestry analyses. The file also includes Supplementary Figs. 1–22, Supplementary Tables 1–3 and additional references. [file 41586_2022_4824_MOESM1_ESM.pdf]

nature portfolio

https://doi.org/10.1038/s41586-022-04824-9

Supplementary information

Grey wolf genomic history reveals a dual ancestry of dogs

In the format provided by the authors and unedited

Supplementary Material

Gray wolf genomic history reveals a dual ancestry of dogs

Supplementary Information Guide

SI Section 1: Mitochondrial phylogeny and dating 2
Assessing the accuracy of the mitochondrial tip dating 2
Chronology adjustment for three specimens informed by autosomal ancestry 5

SI Section 2: Wolf population history analyses 7
Continuous ancestry turnovers in wolf population history 7
Relationships to coyotes and other non-wolf canids 17
qpAdm modelling of post-LGM wolf ancestry 21
Persistence of deep, local ancestries 25
Genetic differentiation analyses (F_{ST}) 29

SI section 3: MSMC2 analyses of N_e history and divergence times 30
Data processing for MSMC2 analyses 30
Population size inferences 31
Divergence time inferences 32

SI section 4: Natural selection analyses 38
Neutral simulations to assess the robustness of the selection scan 38
Inference of TMRCAs along the genome using Relate 43
Genotyping the K^B melanism deletion 44

SI Section 5: Dog ancestry analyses 46
Model-free analyses of dog relationships to wolves 46
f_4-statistics relating dogs to ancient wolves 49
qpWave tests of dog cladality 49
qpAdm modelling of eastern dog ancestry 52
qpAdm modelling of western dog ancestry 55
Testing for a correlation between present-day dog and wolf population structures 59
Reinterpreting evidence for gene flow between dogs and wolves 60
Reinterpreting relationships among early dog lineages 64
Radiocarbon dating of Near Eastern dogs 68

A separate Excel spreadsheet file contains:

Supplementary Data 1: Metadata for ancient wolf genomes
Supplementary Data 2: Metadata and sources of previously published genomic data
Supplementary Data 3: Detailed results from the natural selection scan

A separate text file contains newly reported mitochondrial consensus sequences in fasta format.

SI Section 1: Mitochondrial phylogeny and dating

Assessing the accuracy of the mitochondrial tip dating

In order to test the accuracy of our mitochondrial tip date estimates, we applied a randomization test and a prediction test. The randomization test involves randomly shuffling the prior values for the ages of all ingroup sequences in the phylogeny, and reanalyzing the dataset. Comparing the original mutation rate estimate to that of the date-randomized dataset provides a test of whether the structure and spread of the sequence ages are sufficient to provide reliable information on the rate underlying the evolution of the data set, whereby the original estimate should fall outside of the range of randomized estimates[1]. We found that the rates estimated from the randomized datasets differed significantly from the original estimate in all 20 replicate tests, indicating that the dataset behaves as would be expected from one that has sufficient reliability and power to accurately estimate tip dates (**Fig. S2a**).

In the prediction test, we randomly selected five of the n = 62 ^{14}C dated ancient samples and treated them as if they were undated (uniform prior of 0 to 1,000,000 years before present) in a new phylogeny with identical parameters and number of repeats as in the primary analysis, to see how the resulting predicted dates compared to the known ^{14}C dates. This was then repeated until all n = 62 ^{14}C dated ancient samples had been tested (selected randomly without replacement). For samples where the ^{14}C date fell outside of the 95% Highest Posterior Density intervals of the predicted date, the prediction was repeated where only the age of that single sample was removed. We found that the confidence intervals of the predicted dates overlapped the known radiocarbon dates for all except four samples (CGG16, TH4, SC19.MCJ014 and SC19.MCJ010), and that the average prediction error was 5,133 years (**Fig. S2b**).

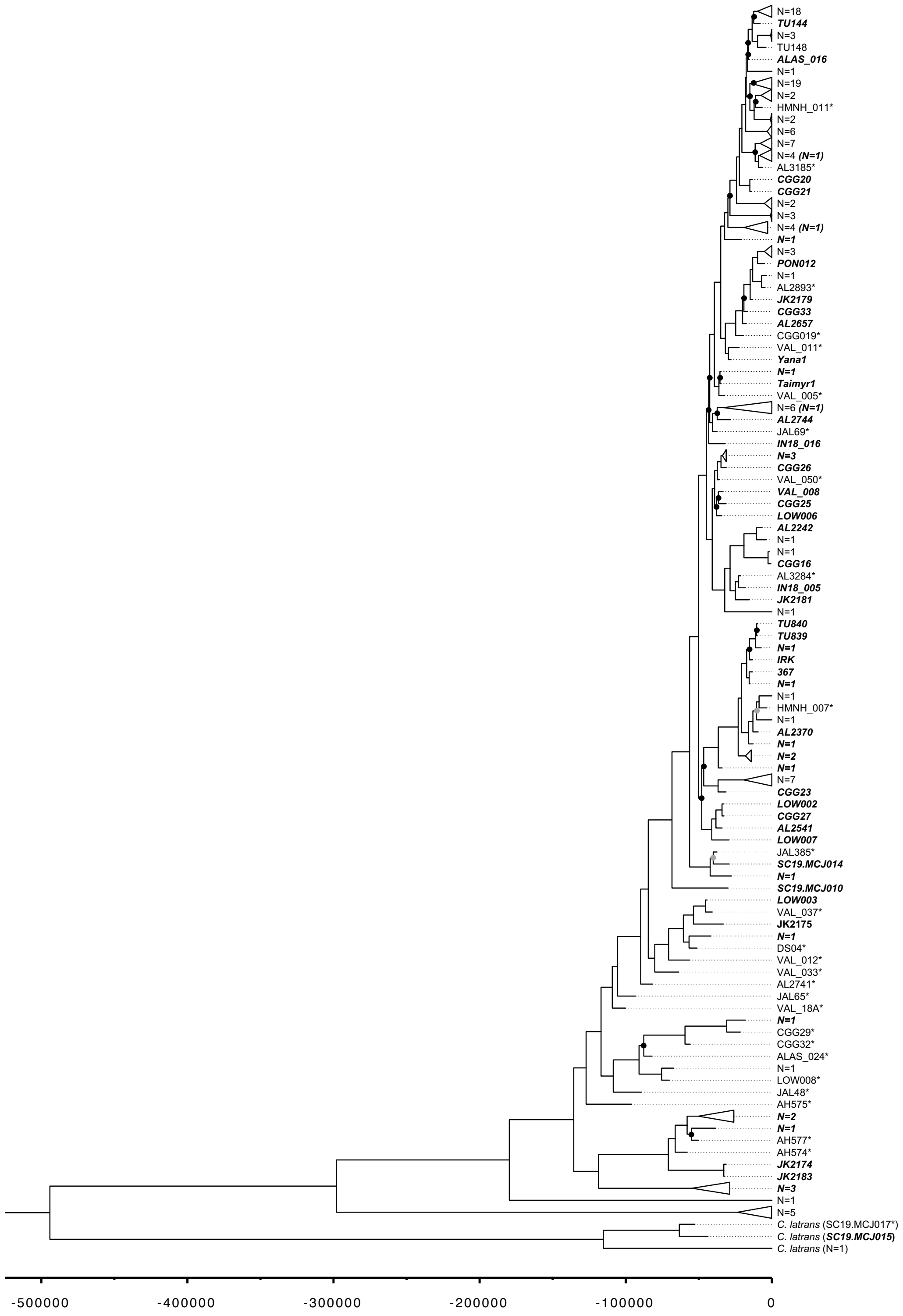

Fig. S1. Mitochondrial phylogeny. Phylogeny of wolf mitochondrial genomes constructed using BEAST v1.10.1[2], used to tip date samples without ^{14}C dates. Samples that are not present in the nuclear dataset are collapsed, with sample numbers shown instead of IDs. Samples for which ^{14}C dates are available are given in bold italics, and samples where tip dates were estimated as a part of the present study are suffixed with an asterisk. Nodes with posterior probabilities less than 0.6 are shown with black dots, those with less than 0.75 are shown with grey dots.

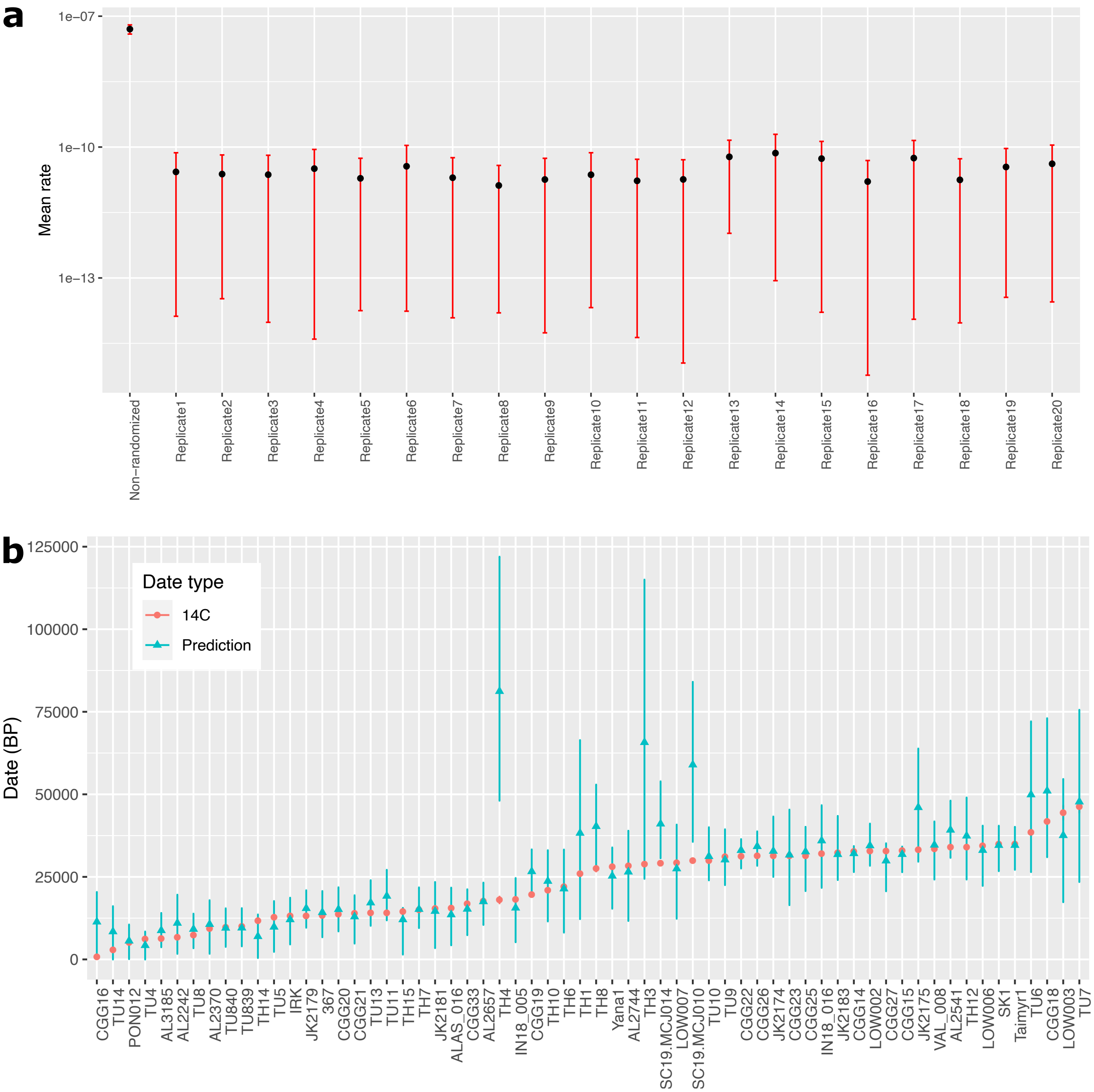

Fig. S2. Assessing the accuracy of mitochondrial tip dating. a) Results of the randomization test whereby the prior values for the ages of all ingroup sequences in the phylogeny were shuffled and the phylogeny was reconstructed. If the new mutation rate estimates from the date-randomized trees are different from the original non-randomized tree, it provides evidence that the structure and spread of the sequence ages are sufficient to provide reliable information on the rate underlying the evolution of the data set[1]. 95% Highest Posterior Density intervals are shown as red whiskers. b) Results of the prediction test, whereby we sequentially treated ^{14}C dated ancient samples as if they were undated and attempted to estimate their age using mitochondrial tip dating. The ^{14}C dates are shown in red and the corresponding tip-date predictions in teal, with bars denoting 95% Highest Posterior Density intervals.

Chronology adjustment for three specimens informed by autosomal ancestry

For three of the ancient wolves, date estimates were obtained on the basis of their autosomal genomes:

- AH575, from Aufhausener Höhle, Germany. This sample had no radiocarbon date, and obtained a mitochondrial tip date of 96,867 years (95% CI: 57,233 - 136,466). This was much older than for two other samples from the same site, AH574 (58,002, 95% CI: 40,480 - 78,292) and AH577 (50,238, 95% CI: 35,908 - 64,814). We tested for any differences in ancestry between AH575 and the other two wolves using f_4-statistics, and across all tests of the form f_4*(AndeanFox,X;AH575,AH574)* and f_4*(AndeanFox,X;AH575,AH577)* where X are other ancient and present-day wolves and dogs, found no significantly non-zero values. Given the strong temporal dimension in wolf relationships discovered across the dataset as a whole, it seems very unlikely that AH575 would be tens of thousands of years older than the other two individuals from the site, yet show indistinguishable ancestry. The mitochondrial tip date of AH575 is therefore likely an overestimate, caused by this individual falling on an isolated branch in the phylogeny and its age therefore not being well-constrained (**Fig. S1**). Rather than using the mean mitochondrial tip date for AH575, we therefore used an age corresponding to the low end of its confidence interval (57,233) for all analyses, which brings it in line with the other two samples from the site.

- CGG13, from Duvannyi Yar, Siberia. This sample has an infinite radiocarbon date, but did not have enough mitochondrial data to be included in the tip dating. Using f_4-statistics of the form f_4*(AndeanFox,X;CGG13,VAL_18A)*, where VAL_18A is the sample in the dataset with the oldest mitochondrial tip date (100,706, 95% CI: 74,464 - 132,716) and X are other ancient and present-day wolves and dogs, we observed positive values for all except three wolves (although most of these were not significantly positive). This implies that all later individuals are more similar to VAL_18A than to CGG13. Given the strong temporal dimension in wolf relationships discovered across the dataset as a whole, it therefore seems likely that CG133 is older than VAL_18A. We therefore used an age equal to that of VAL_18A plus an arbitrary 1000 years: 101,706, for all analyses, but note that it is possibly much older than that.

- AL3272, from Rasik, the Ural mountains. This sample has no radiocarbon date, and did not have enough mitochondrial data to be included in the tip dating. It comes from a layer where other material has infinite radiocarbon dates, suggesting it is quite old. Using f_4-statistics of the form f_4*(AndeanFox,X;AL3272,VAL_18A)* and f_4*(AndeanFox,X;AL3272,AL2741)*, where VAL_18A is a wolf with a mitochondrial tip date of 100,706 (95% CI: 74,464 - 132,716), AL2741 is a wolf with a mitochondrial tip date of 81,793 (95% CI: 60,502 - 106,039), and X are other ancient and present-day wolves and dogs, we observed a trend towards negative values for the former statistic and positive values for the latter statistic. This suggests that AL3272 is intermediate in time, *i.e.* later individuals are closer to AL3272 than to VAL_18A, but closer to AL2741 than to AL3272.

We therefore used an age for AL3272 equal to the midpoint between those of the other two wolves: 91,250.

These three individuals for which the chronology was adjusted based on autosomal ancestry were kept out when calculating the correlation between sample age and PC1 from the PCA on outgroup f_3-statistics (**Fig. 1c**), because the latter statistics are capturing essentially the same autosomal ancestry information as the f_4-statistics used above to assign dates, which would give rise to a circularity that would inflate the correlation.

SI Section 2: Wolf population history analyses

Continuous ancestry turnovers in wolf population history

We used various *f*-statistics to study relationships between wolf genomes. First, we used outgroup f_3-statistics of the form $f_3(X,Y;CoyoteCalifornia)$ where Y is a fixed present-day wolf (or dog) individual, and X are other ancient and modern individuals. This statistic measures the amount of genetic drift that is shared by X and Y since their divergence from the coyote. We used these statistics in two ways:

- First, we performed a principal components analysis (PCA) on a matrix of f_3 values (**Methods**), which recovered a first principal component that strongly correlated with sample age (**Fig. 1c**). No meaningful additional principal components were recovered. Time is thus the primary variable explaining genetic similarities between wolves, with all individuals living in a given time period—regardless of whether they live in Europe, Siberia or North America—being genetically similar to each other. Several North American wolves are exceptions to this trend, and do not cluster with other individuals, which is explained by their coyote admixture. Coyote admixture dilutes the amount of genetic drift that a given wolf shares with other wolves, making it look 'older' on this principal component.

- Second, we studied these f_3-statistics directly, for various choices of present-day genomes Y. This revealed that the amount of genetic drift that a given ancient wolf X shares with a present-day individual increases steadily over the Late Pleistocene as the sample age of X approaches the present day (**Extended Data Fig. 1a, Fig. S3**). This is true for all choices of present-day genomes Y, including dogs. The shape of the f_3 values plotted over time actually greatly resembles the PCA results (**Fig. 1c**), and like in the PCA several North American wolves with coyote admixture are shifted downwards. If there were large-scale replacement events in wolf history, e.g. if the ancestors of present-day wolves had suddenly replaced older, divergent populations that they did not share much genetic drift with, we would expect to see a sudden increase in f_3 values at the time of replacement. The lack of any observed discontinuities over time thus argues against any dramatic replacement events, and instead suggests that strong genetic connectivity over time is driving the observed patterns.

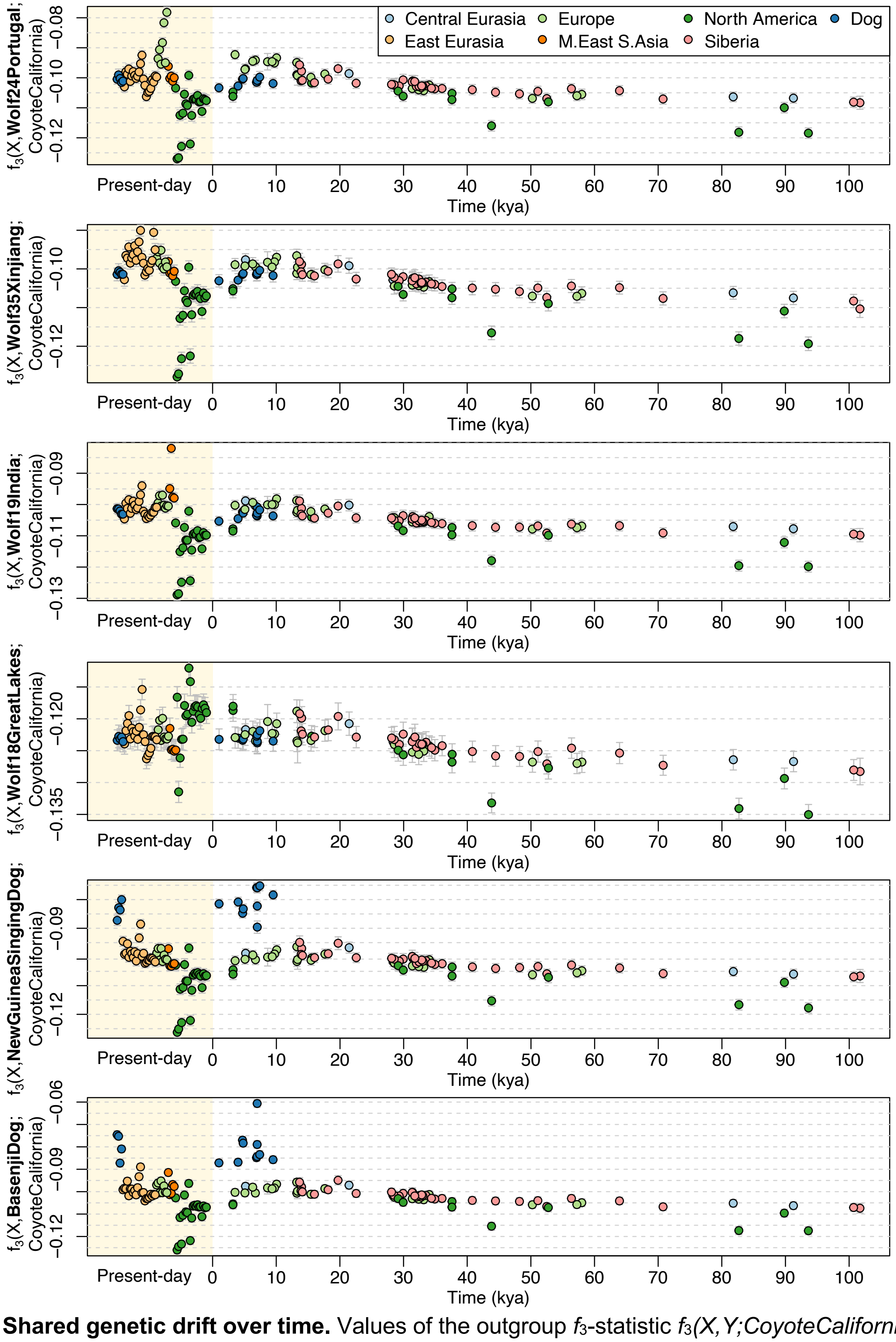

Fig. S3. Shared genetic drift over time. Values of the outgroup f_3-statistic $f_3(X,Y;CoyoteCalifornia)$ for six different present-day individuals Y (bold), quantifying how much genetic drift they share with other individuals X. Only sites ascertained as heterozygous in CoyoteCalifornia were used, which causes the nominal values to be negative. Bars denote ±1.96 standard errors estimated from a block jackknife.

We also studied f_4-statistics of the form f_4*(Armenia_Hovk1.HOV4,X;Younger wolf,Older wolf)*. This uses the ancient dhole *Armenia_Hovk1.HOV4* as an outgroup, and for every given individual X asks if X is more similar to a more recent wolf (giving rise to negative values) or to an older wolf (giving rise to positive values). We found that, when contrasting a more recent wolf and an older wolf separated in time by at least 10-20k years, later individuals (i.e. living closer to the present day) are always more similar to the more recent wolf. This is the case throughout the whole time series (**Fig. S4**). For individuals that predate the older wolf, the statistics tend to be around 0, meaning they are equally close to the more recent and the older wolf.

These patterns are observed in the period around the last glacial maximum (LGM), where all post-LGM individuals (including present-day individuals) are more similar to other post-LGM wolves than to pre-LGM wolves. This is consistent with a LGM turnover as hypothesised by previous studies[3–5], but our results also show that this dynamic is not a novel phenomenon in wolf history. Instead, the same pattern—later individuals being more similar to more recent wolves than to older wolves—is manifested throughout the last 100,000 years. These results thus suggest that what occurred around the time of the LGM was not necessarily a dramatic replacement event, but simply the most recent manifestation of a process that had been ongoing for as long as our data can tell.

Patterns like those observed here (**Fig. S4**) are what would be expected in a panmictic population moving forward in time, in which individuals at a more recent time point t_3 are simply the descendents of earlier individuals at time point t_2, who in turn are the descendents of even earlier individuals at time point t_1, etc. In such a population, samples from t_3 will always be more similar to samples from t_2 than t_1, meaning $f_4(Outgroup,t_3;t_2,t_1)$ will be negative. Samples that are older than both t_2 and and t_1, e.g. from a time point t_0, will be equally similar to all later, descendent populations, meaning $f_4(Outgroup,t_0;t_2,t_1)$ will be zero. To further understand expectations in a panmictic population, we simulated a single population without structure and with constant effective population size (N_e = 50,000) and calculated $f_4(Outgroup,X;t_2,t_1)$ for individuals X sampled over time (this used the same simulations as used for the selection analyses, see SI section 4 further details). The patterns observed in this simulated panmictic population (**Fig. S5**) are very similar to those observed in the empirical data (**Fig. S4**).

Other analyses reveal that there is some degree of geographical structure among Late Pleistocene wolves that persists over time, meaning they are not actually fully panmictic, but these temporal patterns suggest that strong gene flow is connecting all populations. Over a time scale of ~10-20k years, all wolves across the geographical regions sampled here become more genetically similar to other contemporaneous wolves than to earlier individuals living in the same place.

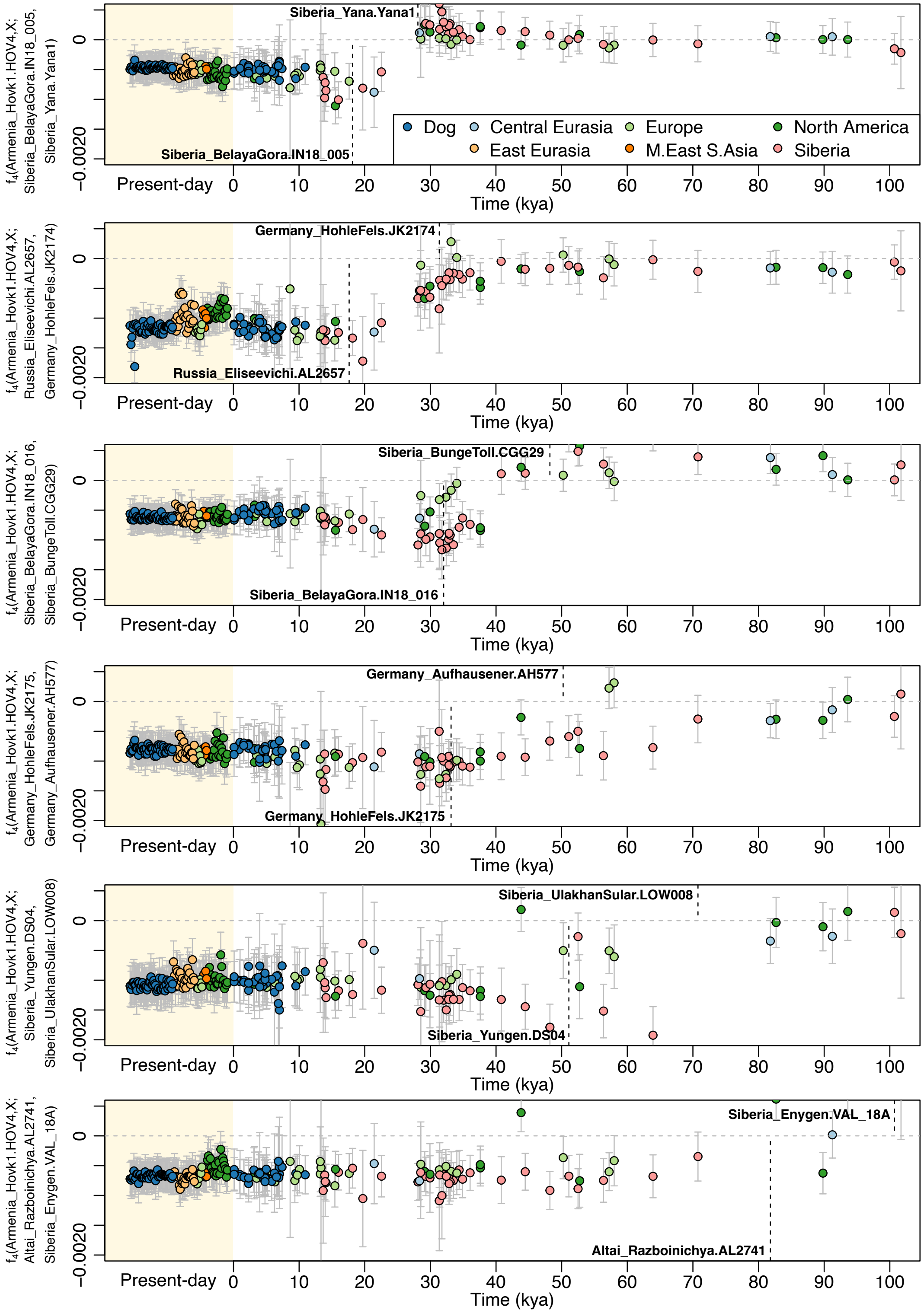

Fig. S4. Continuous ancestry turnovers in wolf population history. f_4-statistics of the form f_4*(Armenia_Hovk1.HOV4,X;Younger wolf,Older wolf)*, where Armenia_Hovk1.HOV4 is an ancient dhole serving as an outgroup and X are individuals displayed in the plots. The ages of the two wolves being contrasted are indicated with dashed lines, with positive values indicating affinity to the upper individual (older) and negative values indicating affinity to the lower (younger) individual. Bars denote ±3 standard errors estimated from a block jackknife.

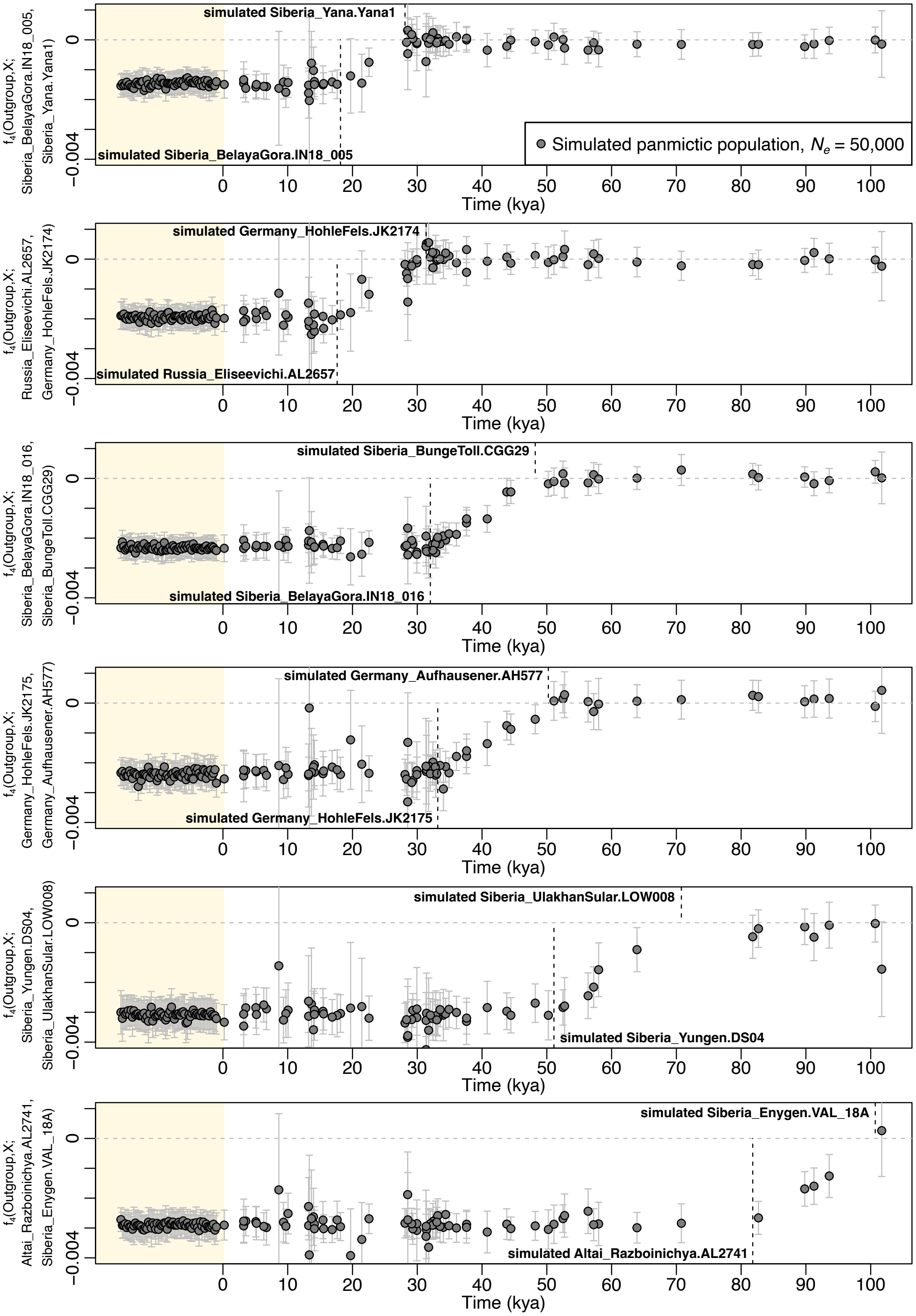

Fig. S5. Temporal relationships in a panmictic simulation. Genomes were sampled from a panmictic simulation to match the empirical genomes, and *f_4(Outgroup,X;Younger wolf,Older wolf)* were calculated, where Outgroup is sampled at 1000 kya and X are displayed in the plots. The ages of the two genomes being contrasted are indicated with dashed lines, with positive values indicating affinity to the upper individual (older) and negative values indicating affinity to the lower (younger) individual. Bars denote ±3 standard errors estimated from a block jackknife. The plots match those for the empirical data in **Fig. S4**.

Next, we wanted to understand if there's any geographical signal in the gene flow that connects wolf populations over time. To do this, we studied f_4-statistics of the form f_4*(Armenia_Hovk1.HOV4,X;A,B)*. This uses the ancient dhole *Armenia_Hovk1.HOV4* as an outgroup, and asks for every given individual X if X is more similar to the ancient wolf A or the ancient wolf B, where A and B are two wolves that lived at a similar point in time but in different geographical regions (**Extended Data Fig. 1c, Fig. S6**).

Contrasting a Siberian wolf and a European wolf that both lived ~32 kya, i.e. not long before the LGM, revealed that post-LGM individuals tend to be more similar to the Siberian wolf. Similarly, contrasting a Siberian wolf and a central Asian (Altai mountains) wolf that both lived ~28 kya, post-LGM individuals tend to be more similar to the Siberian wolf. This thus suggests that the ancestry that connects all wolves living after the LGM is coming from Siberia, or at least a population that is related to the Siberian wolves that we have sampled. This is consistent with analyses of mitochondrial genomes which suggested an ancestry expansion out of Beringia at the end of the LGM[4]. Looking at a similar contrast but earlier in time, contrasting a Siberian wolf and a European wolf that both lived around ~50 kya, there is a very similar pattern: individuals living more recently are more similar to the Siberian wolf. Gene flow from Siberian, or Siberian-related, wolves thus seems to be driving the ancestry homogenization throughout the Late Pleistocene.

These analyses also show that the ancestry turnovers are not complete. For example, in the first contrast between wolves that lived at ~32 kya, while all post-LGM wolves are Siberian-shifted, post-LGM European (as well as present-day Middle Eastern & South Asian) wolves are slightly less Siberian-shifted. A similar pattern is observed in the contrast between wolves that lived at ~50 kya. This suggests that west Eurasian wolves are retaining some degree of deep, local ancestry, and are not fully replaced by wolves from Siberia.

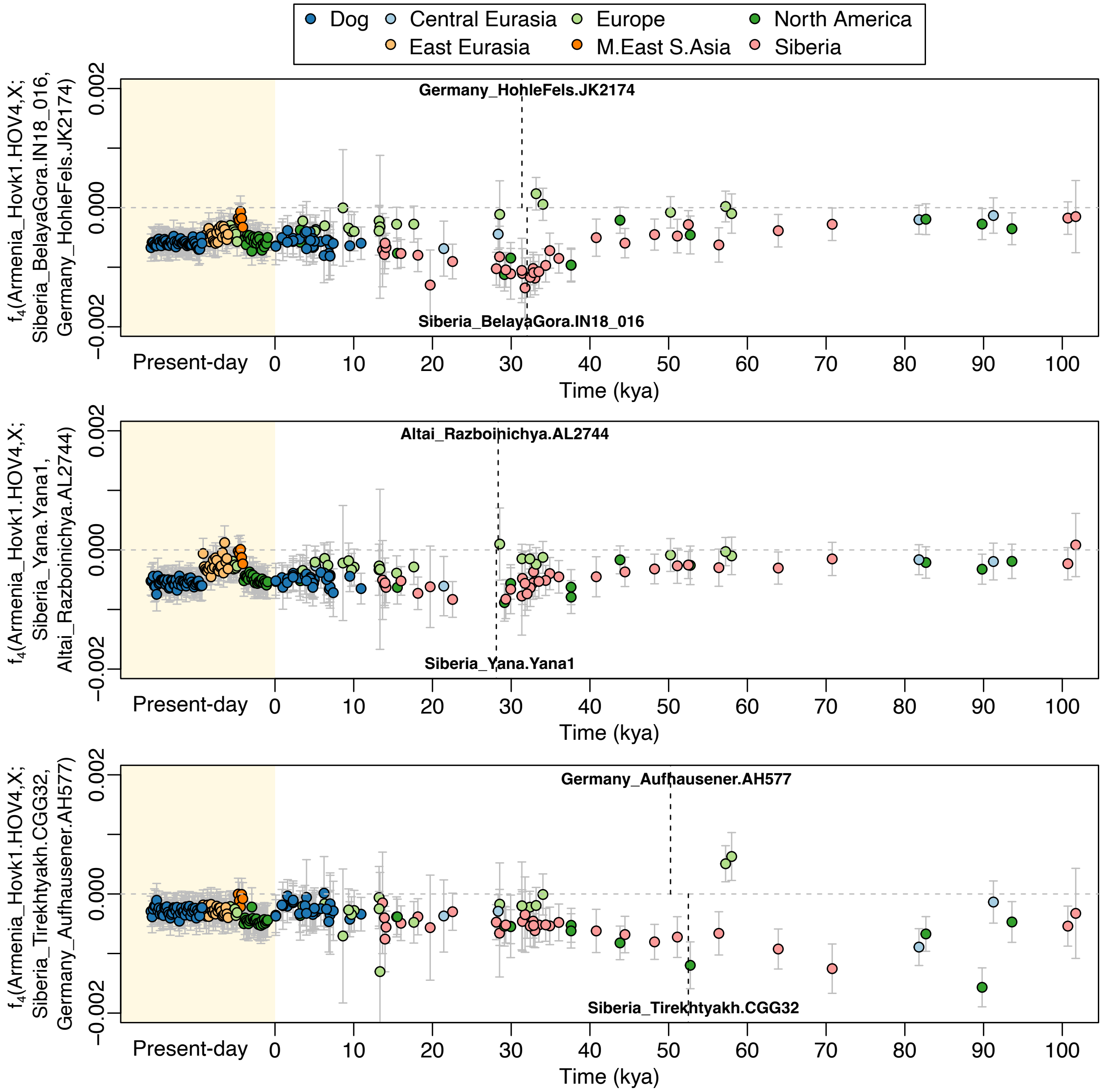

Fig. S6. Geographical drivers of ancestry turnovers. f_4-statistics of the form f_4*(Armenia_Hovk1.HOV4,X;A,B)*, where Armenia_Hovk1.HOV4 is an ancient dhole serving as an outgroup, A and B are two ancient wolves living at approximately the same time in different geographical regions, and X are other individuals displayed in the plots. The ages of the two wolves being contrasted are indicated with dashed lines, with positive values indicating affinity to the upper individual and negative values indicating affinity to the lower individual. Bars denote ±3 standard errors estimated from a block jackknife. Upper plot: later individuals tend to be more similar to a Siberian than a European wolf living at ~32 kya. Middle plot: later individuals tend to be more similar to a Siberian than to an Altai mountains wolf living at ~28 kya. Lower plot: later individuals tend to be more similar to a Siberian wolf than a European wolf living at ~51 kya.

To further understand the geographical signal in the gene flow among wolf populations over time, we also studied f_4-statistics of another form: f_4*(Armenia_Hovk1.HOV4,Old wolf A;Old wolf B,X)*. This uses the ancient dhole *Armenia_Hovk1.HOV4* as an outgroup, and two fixed ancient wolves A and B which lived at a similar time but in different geographical regions. For each other individual X that lived more recently, this statistic then asks whether A is more similar to B (giving rise to a negative value) or to X (giving rise to a positive value). Since gene flow cannot occur backwards in time, a positive value cannot be caused by gene flow from X to A, and instead implies gene flow from A to X.

These statistics showed that Siberian wolves that lived ~60 kya are more similar to more recent European wolves than to European wolves that also lived ~60 kya, which means there must have been Siberian-related gene flow into the more recent European wolves (**Extended Data Fig. 1d, Fig. S7**). However, the inverse is not the case: European wolves that lived ~60 kya are equally close to more recent Siberian wolves and Siberian wolves that also lived ~60 kya, which suggests there was no detectable gene flow from Europe into the more recent Siberian wolves. Looking at the same type of statistics but centred on wolves that lived ~32 kya reveals the same pattern. It thus appears that, throughout the Late Pleistocene, European wolves become more Siberian-like over time, but Siberian wolves do not become more European-like.

On the basis of these observations, we constructed and fit an admixture graph to a subset of the ancient genomes, featuring unidirectional gene flow events from Siberia into Europe. We also grafted on a number of ancient North American wolves as products of admixture between Siberian branches from a similar point in time as the given North American wolf, and coyote. We did not attempt a more systematic search of the space of possible admixture graphs (which would be very large for this number of populations), and we think it's certainly possible that there are variations of this graph that will fit the data equally well. But the above f_4 analyses support the central aspects of the graph, namely that Siberia acts as the ancestry epicentre throughout the Late Pleistocene, with gene flow from here into the other regions, but not from the other regions into Siberia (**Fig. 2a**, **Fig. S8**).

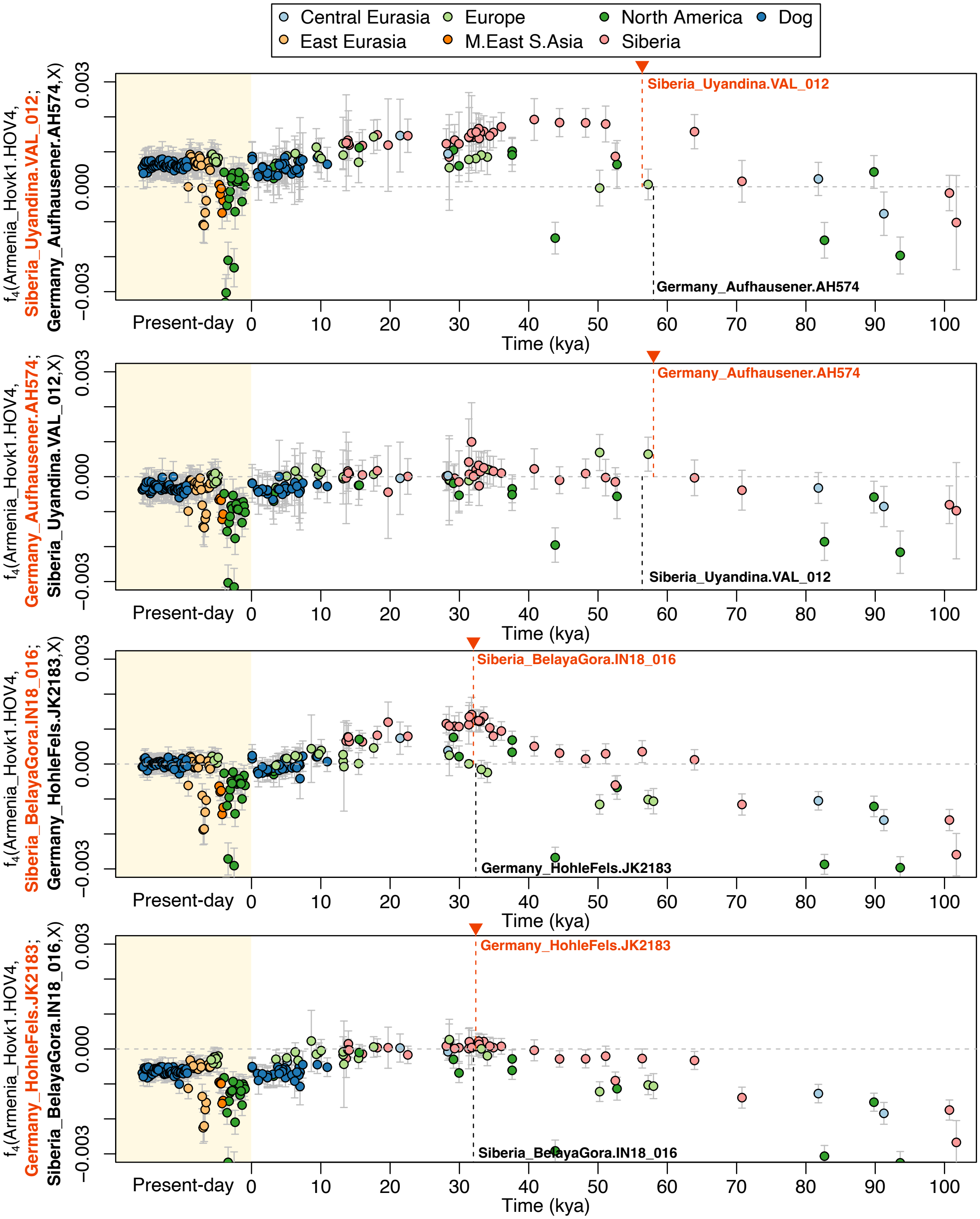

Fig. S7. European wolves become more Siberian-like over time, but not vice versa. f_4-statistics of the form f_4*(Armenia_Hovk1.HOV4,A;B,X)*, where Armenia_Hovk1.HOV4 is an ancient dhole serving as an outgroup, A and B are two ancient wolves living at a similar time and X are other individuals displayed in the plots. Bars denote ±3 standard errors estimated from a block jackknife. For an X living later than A and B, a positive value indicates that A (in orange, with dotted line and arrowhead indicating its age) is closer to X than to B (block dotted line), meaning there must have been gene flow into X from a source related to A. The results indicate gene flow from Siberia into Europe (first and third plot), but not from Europe into Siberia (second and fourth plot).

snps: 223067

Outlier statistics	Fit	Obs	Diff	Std. error	Z
f_4(Yukon_IndependenceCreek.SC19.MCJ014,Siberia_Tirekhtyakh.VAL_033; Canada_Umingmak.TU144,Siberia_BelayaGora.IN18_005)	0.000007	-0.001278	-0.001285	0.000427	-3.006
f_4(Siberia_Enygen.VAL_18A,Siberia_Tirekhtyakh.VAL_033; Siberia_BelayaGora.IN18_016,Siberia_BelayaGora.IN18_005)	0.000000	-0.001322	-0.001322	0.000419	-3.157

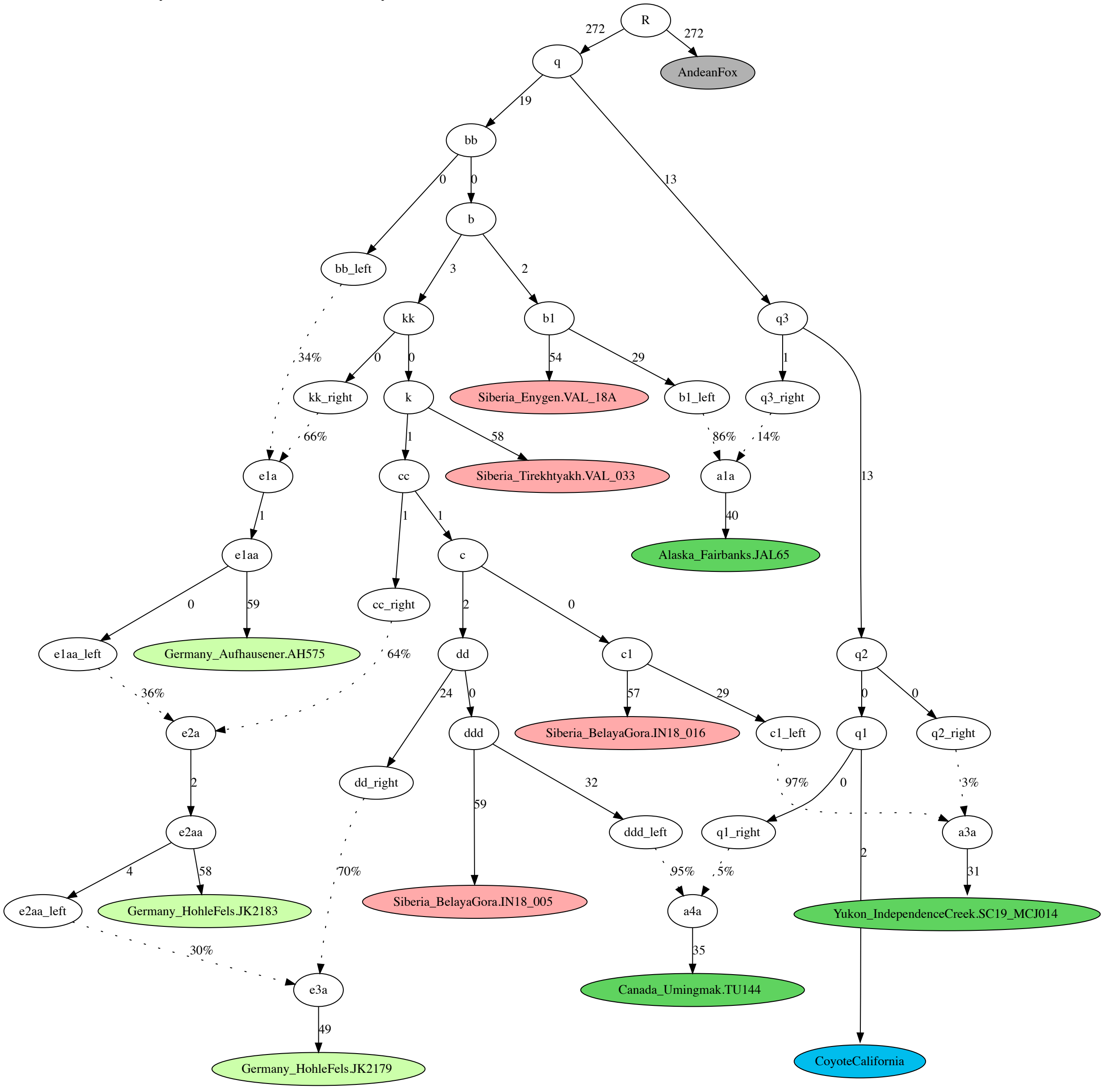

Fig. S8. Admixture graph model of wolf population history. Dot visualization of the admixture graph redrawn in **Fig. 2a**. Observed and fitted values for the two outlier f_4-statistics are displayed above the graph.

Relationships to coyotes and other non-wolf canids

We calculated the f_4-statistic f_4*(AndeanFox,CoyoteCalifornia;Siberia_Enygen.VAL_18A,X)* for all ancient and present-day wolves, using the ~100k-year old Siberian wolf *Siberia_Enygen.VAL_18A* as a baseline for coyote affinity. A positive value of this statistic implies that the coyote is more similar to X than to *Siberia_Enygen.VAL_18A*. Siberian wolves across the time series display values consistent with 0 for this statistic, meaning that they display the same coyote affinity as *Siberia_Enygen.VAL_18A*. This implies that there is no influx of coyote ancestry into Siberian wolves during the last 100k years, nor any dilution of coyote affinity due to admixture from some unsampled lineage that is more deeply divergent than coyote (**Extended Data Fig. 1e**).

However, European wolves display slightly negative values of the statistic f_4*(AndeanFox,CoyoteCalifornia;Siberia_Enygen.VAL_18A,X)*, implying that the coyote is slightly closer to the Siberian wolves than to the European wolves. This signal is strongest for the earliest European genomes at 60-50 kya, but is to some extent visible in later individuals. To further explore this, we extended the analysis beyond the single *Siberia_Enygen.VAL_18A* individual to all possible f_4-statistics of the form f_4*(AndeanFox,CoyoteCalifornia;X,Y)*, where X are ancient individuals from one geographical region, and Y are ancient individuals from another geographical region (ancient genomes of all ages were pooled together by region). Plotting these in quartile-quartile plots reveals systematic differences in the coyote affinity of different populations in the form of deviations from the diagonal (**Fig. S9**). These results confirm the higher coyote affinity in Siberian wolves compared to European wolves, and reveal a similarly higher affinity in Siberian wolves compared to central Eurasian wolves. Siberian wolves also show higher coyote affinity than dogs, suggesting that the wolf ancestors of dogs had lower coyote affinity than Siberian wolves.

These results can thus be described as a gradient of coyote affinity across ancient Eurasian wolves, with the highest affinity in Siberia and the lowest in Europe. Two different scenarios could explain this pattern. First, there could have been gene flow from coyotes (including gene flow mediated by wolves carrying minority fractions of coyote ancestry) into Siberian wolves before the earliest Siberian wolves in our dataset lived, i.e. prior to 100 kya. Second, there might be some amount of wolf admixture in the *CoyoteCalifornia* individual used in these f_4-statistics. If that wolf admixture came from a population that was more similar to Siberian than to other Eurasian wolves - as would be expected if that source population was North American wolves - then that would give rise to the observed higher affinity to Siberian wolves. We cannot distinguish between these two scenarios, but given the widespread coyote admixture observed in North American wolves, it seems probable that there would be some amount of wolf admixture in coyotes. The second possibility, admixture from Siberian-related wolves into coyotes, thus seems like the more likely explanation. One implication of these results is that, since coyotes are not fully symmetrically related to wolf and dog populations in Eurasia, the coyote is not a good choice for outgroup in analyses of Eurasian wolf and dog relationships.

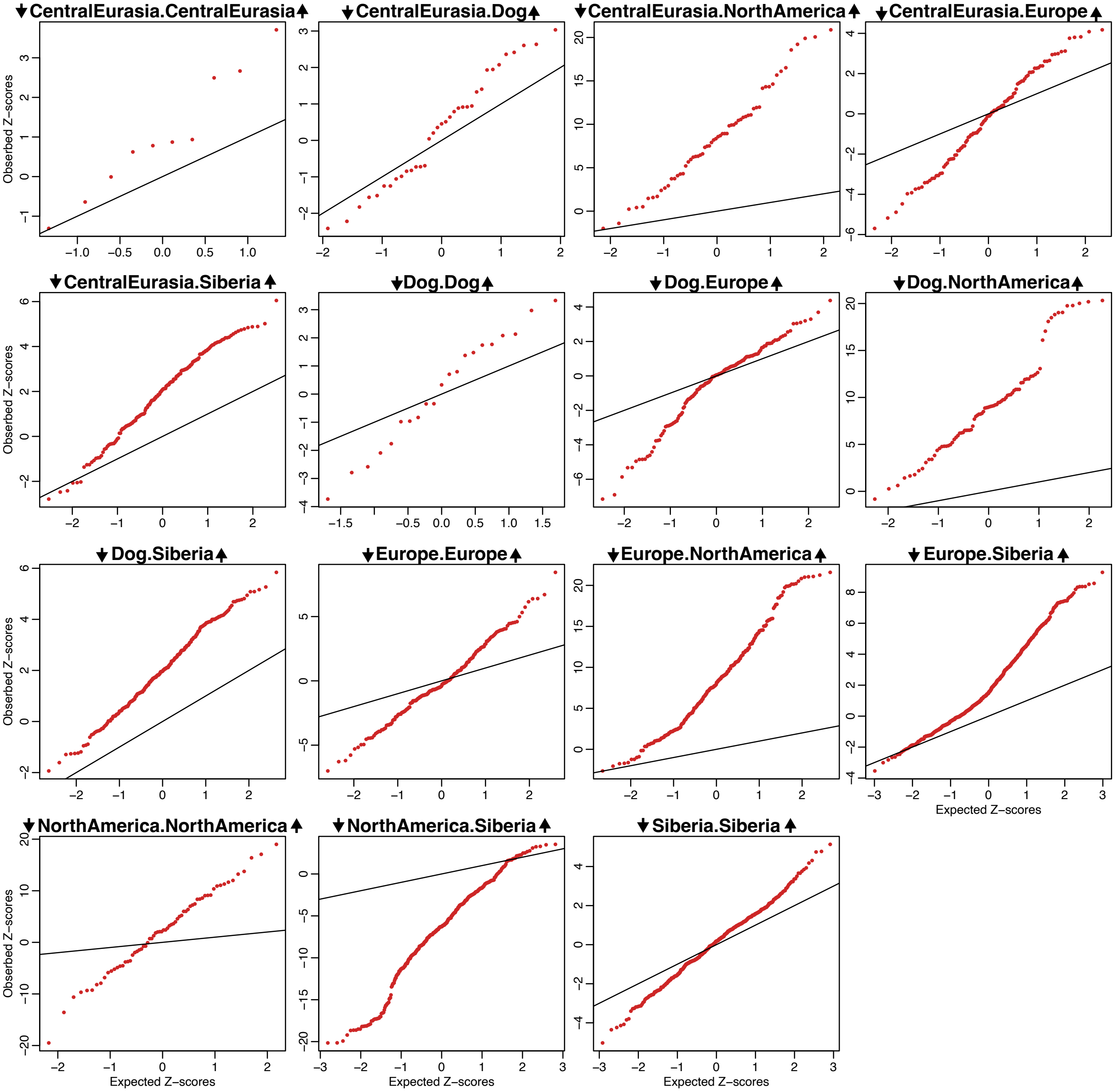

Fig. S9. Asymmetries in the relationships to coyotes. Quartile-quartile (QQ) plots of the Z-scores from f_4-statistics of the form f_4*(AndeanFox,CoyoteCalifornia;X,Y)*, where X are individuals from one geographical region, and Y are individuals from another geographical region. Values observed in the data are plotted on the vertical axis, and values expected under a normal distribution (calculated using the qnorm R function) on the horizontal axis. Only ancient genomes are included (ancient American dogs are excluded due to their known affinity to coyotes). Negative values of the observed statistic imply that coyote is closer to X than to Y, while positive values indicate coyote is closer to Y than to X (as also indicated by the arrows next to the regions in each plot, which relate to the values on the vertical axis). North American wolves show very strong coyote affinities relative to all other regions, but Siberian wolves also show stronger affinities than European and Central Eurasian wolves.

We also analysed relationships to other non-wolf canid species: golden jackal, African golden wolf, and Ethiopian wolf. We calculated f_4-statistics of the form f_4*(AndeanFox,Y;Siberia_Enygen.VAL_18A,X)*, where Y is one of the non-wolf canid species, Siberia_Enygen.VAL_18A is a ~100k-year old Siberian wolf and X are other individuals displayed in the plots. The Siberia_Enygen wolf thus acts as an ancestry baseline. A positive value implies that the species Y is closer to X than to Siberia_Enygen, which could be caused by gene flow between X and Y (in either direction), or by unsampled, divergent (or "deep") ancestry in Siberia_Enygen. Given that Siberia_Enygen is ~100k years old, it seems unlikely that it would have some deep ancestry that is not shared with later wolves, and so positive statistics should likely primarily reflect gene flow between X and Y (**Fig. S10**).

Golden jackal, African golden wolf and Ethiopian wolf all display positive values for many present-day Eurasian wolves and dogs, though the direction of the gene flow cannot be determined from these f_4-statistics. For African golden wolves and Ethiopian wolves, the signal is strongest for Near Eastern and South Asian wolves. Across the ancient time series there is not much change visible, and there are no sudden jumps in the affinity to these non-wolf species. A slight increase towards the present-day might be visible in, for example, the African golden wolf, but this does not allow for the direction of gene flow to be inferred: a correlation to time would be expected if there was continuous gene flow from African golden wolves into wolves over time, but it would also be expected if there was gene flow from recent wolves into African golden wolves (because moving forward in the ancient time series we get closer and closer to the source of that gene flow). In any case, these results confirm a likely complex history of admixture between wolves and these other species in southern Eurasia and/or Africa[6]. However, the continuous and largely stable levels of affinity across the ancient wolf time series, especially in Siberia, gives no strong reason to suspect that gene flow from other species made a substantial impact on the species-wide, shared ancestry of wolves throughout the last 100 ky.

A negative value implies that species Y is closer to Siberia_Enygen than X, which could be caused by gene flow from Y into Siberia_Enygen, or by deep ancestry in X. Given that Siberia_Enygen is ~100k years old, it seems unlikely that it would have gene flow from non-wolf species that is not shared with later wolves, and so negative statistics should likely primarily reflect deep ancestry in X. As an example of this, golden jackal and African golden wolf display negative values for many North American wolves, reflecting the coyote admixture in the latter. This also implies that at least part of the ancestry of golden jackal and African golden wolf is less divergent from gray wolves than what coyote ancestry is. Coyote, golden jackal and African golden wolf display slightly more negative values for wolves from western China (Tibet, Inner Mongolia, Qinghai) than for other Eurasian wolves, suggesting these Chinese wolves have a partial component of ancestry that is more divergent than these species. However, due to the possibility of admixture from non-Chinese wolves into these species (which could push the statistics in the same direction), it's difficult to say if this reflects admixture from an unknown lineage in the Chinese wolves, or just deep, local gray wolf ancestry. In any case, it likely reflects the same divergent ancestry previously reported for wolves in Tibet and the Himalayas[7]. There are also highly divergent mitochondrial lineages in these wolf populations[8,9], which plausibly reflect the same source of ancestry.

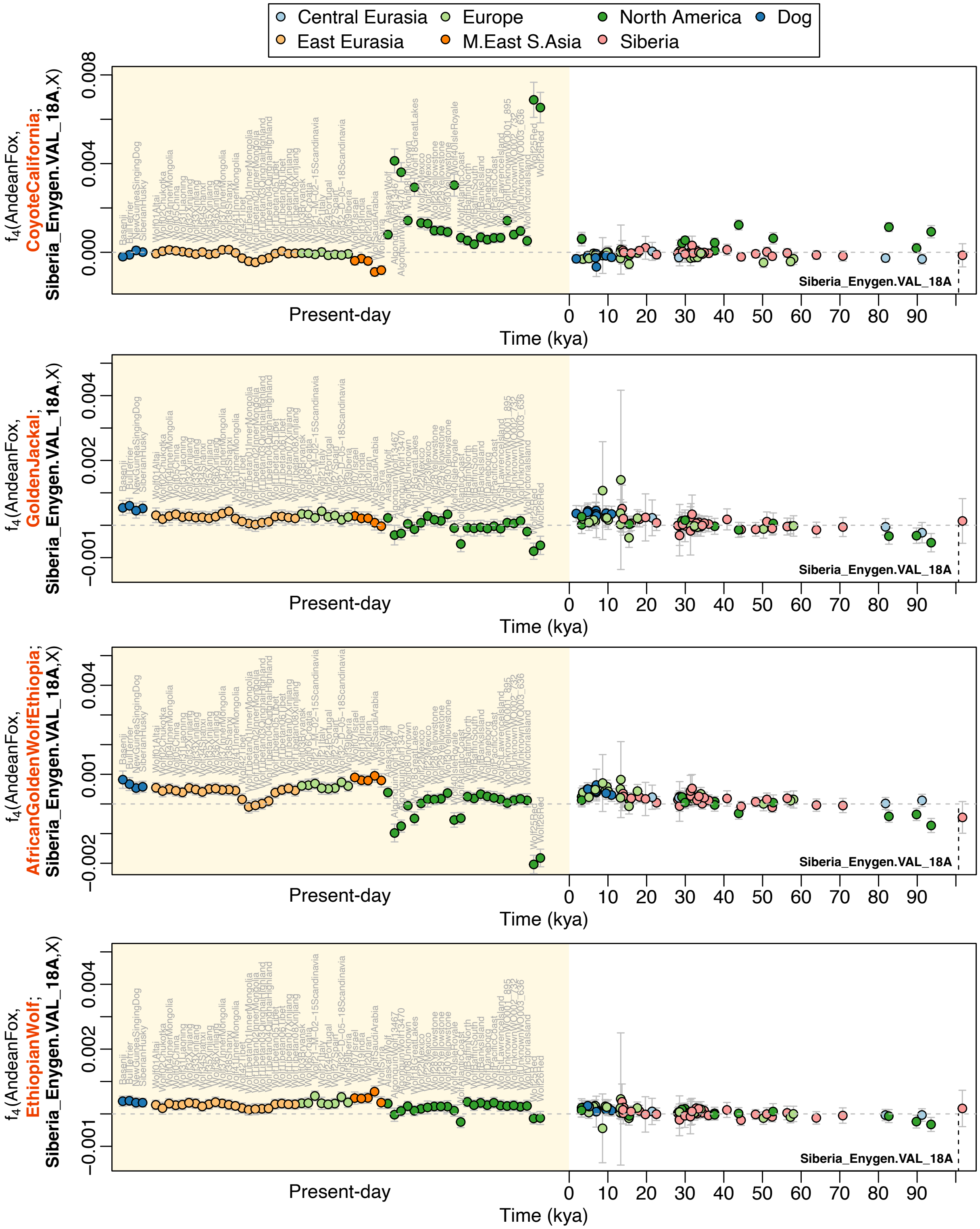

Fig. S10. Testing for ancestry more divergent than, or related to, other canid species. f_4-statistics of the form f_4*(AndeanFox,Y;Siberia_Enygen.VAL_18A,X)*, where Y is a non-wolf canid species, Siberia_Enygen.VAL_18A is a ~100k-year old Siberian wolf (bold, with dotted lines indicating its age) and X are other individuals displayed in the plots. A positive value of this statistic implies that the non-wolf species is closer to X, and a negative value implies it's closer to Siberia_Enygen.VAL_18A. Bars denote ±3 standard errors estimated from a block jackknife.

qpAdm modelling of post-LGM wolf ancestry

We used the *qpAdm* and *qpWave* programs from ADMIXTOOLS 5.0[10] to test ancestry models for wolf and dog targets post-dating 25 kya. For the primary analyses we used the following set of candidate source populations (age estimate in parenthesis, years BP):
Armenia_Hovk1.HOV4 (Ancient dhole)
Siberia_UlakhanSular.LOW008 (70,772)
Germany_Aufhausener.AH575 (57,233)
Siberia_BungeToll.CGG29 (48,210)
Germany_HohleFels.JK2183 (32,366)
Siberia_BelayaGora.IN18_016 (32,020)
Yukon_QuartzCreek.SC19.MCJ010 (29,943)
Altai_Razboinichya.AL2744 (28,345)
Siberia_BelayaGora.IN18_005 (18,148)
Germany_HohleFels.JK2179 (13,229)

We employed a rotating approach, where for each target we tested all possible one, two and three-source models that can be enumerated from the above set. Individuals from the set that were not used as a source in a given model served as the reference set (or the "right" population in the *qpAdm* framework). This means that in every model, each of the above individuals was either in the source list or in the reference list. We ranked models based on their p-values, but prioritised models with fewer sources using a p-value threshold of 0.01: if a simpler (meaning fewer sources) model had a p-value above this threshold, it ranked above a more complex (meaning more sources) model regardless of the p-value of the latter. We also failed models with inferred ancestry proportions >1.1 or <-0.1. For single-source models, the *qpWave* program was run instead of *qpAdm*. Both programs were run with the "allsnps: YES" option since, without this option, there was very little power to reject models.

An outgroup species is included in the above set - we used the ancient dhole *Armenia_Hovk1.HOV4*, but results were similar when using a coyote or Andean fox. As in the additional discussion below, and in the context of dog ancestry, models that featured the outgroup population are favoured for certain targets, but we do not interpret this as reflecting actual ancestry from that species in the target. Rather, we interpret it as reflecting ancestry that is divergent from, and lacking shared genetic drift with, the available wolf genomes in the above set - and we refer to it as "unsampled ancestry", "divergent ancestry" or "deep ancestry". This is similar to how African populations that are outgroups to non-Africans can be used as proxies for "basal Eurasian" or "deep" ancestry in non-African human populations[11]. However, we note that since the private drift in the true, unsampled source population is not represented by the outgroup proxy, the degree of divergence of the unsampled source and the proportion of ancestry deriving from it cannot necessarily be disentangled. The inferred proportions of unsampled ancestry might thus not be accurate, and we do not attach much importance to them.

North American wolves are among those targets that required an outgroup component to achieve a good fit in *qpAdm*. In this case, the previous literature and other analyses make it clear that the source of this ancestry is coyotes. Therefore, when modelling North American targets, we used *CoyoteCalifornia* in place of *Armenia_Hovk1*.HOV4 in the above set, as this more proximate source should lead to better model fits and more accurate admixture proportions. Other than this, the analysis set-up for North American wolves was the same as for other wolves.

Applying this *qpAdm* analysis to all post-LGM and present-day wolves provided results for wolves in different parts of the world (**Fig. 2b**). Due to the strongly temporal patterns driving wolf relationships, the most recent genomes included as available sources—the 18 ky old *Siberia_BelayaGora.IN18_005* and the 13 ky old *Germany_HohleFels.JK2179*—always come out as the best sources. While these sources in themselves have complex histories tracing back over the past 100,000 years, in what follows we refer to ancestry assigned to them by *qpAdm* simply as “Siberian-related” and “European-related” ancestry, respectively. Other analyses (e.g. **Fig. 2a**) show that post-LGM European wolves ultimately trace much of their ancestry to Siberia, but by using the 13 ky old *Germany_HohleFels.JK2179* we ‘screen off’ that deeper history and refer to European wolf ancestry as it looked at the time that this individual lived.

- **Siberia**: Post-LGM Siberian wolves, available in the period 23-13 kya but not more recently than that, are well-modelled as having 100% ancestry related to the 18k-year old *Siberia_BelayaGora.IN18_005*. This is not surprising since that source is from the same time period and geographical region as these targets. However, the fit of this single-source model implies that there is no evidence, within the resolution of our data, for the persistence of deeper population structure within Siberia - the older Siberian wolves can be rejected as contributors of additional ancestry when *Siberia_BelayaGora.IN18_005* is available as a source.

  Only one present-day Siberian wolf is available, Wolf02Chukotka. The single-source Siberia model is rejected for this individual ($p = 1.7 \cdot 10^{-12}$), in favour of models that also feature the 13k-year old European wolf *Germany_HohleFels.JK2179* as a source. The single-source Europe model is not rejected ($p = 0.072$), though other analyses show an Eastern Eurasian affinity for this individual (**Extended Data Fig. 2**) meaning that this is probably a failure to reject a false model. While the two-source Europe + Siberia model performs slightly less well ($p = 0.01$), it models Wolf02Chukotka as 24% Siberia-related and 76% Europe-related, which may be more accurate. In any case, this demonstrates that the ancestry continuity that seems to have characterised Siberia through the late Pleistocene does not extend up until the present-day, as this present-day individual has European-related ancestry. Ancient Siberian wolf genomes from the last 14 kya will be needed to uncover what process resulted in this.

- **Europe**: Most post-LGM, Holocene and present-day European wolves are well-modelled as having 100% ancestry related to the 13k-year old European wolf *Germany_HohleFels.JK2179*. For many late Pleistocene and early Holocene wolves,

this is not surprising, as that source is from the same time period and geographical region as these targets. However, the fit of this single-source model implies that there is no evidence for the persistence of deeper population structure within Europe - the older European wolves can be rejected as contributors of additional ancestry when *Germany_HohleFels.JK2179* is available as a source.

Two exceptions to this rule are an 18k-year old wolf from Eliseevichi in western Russia that is modelled as 48% European-related and 52%-Siberian related, and a 21k-year old wolf from Shaitanskaya Cave in the Ural mountains that is modelled as 17% European-related and 83% Siberian-related. This demonstrates substantial amounts of Siberian-related ancestry in, or just east of eastern Europe, at the end of the LGM, and could plausibly reflect a west-east gradient of European versus Siberian-related ancestry across northern Eurasia.

The observation that all present-day European wolves are also well-modelled by the single-source *Germany_HohleFels.JK2179* model implies ancestry continuity within Europe over the last 13 ky. The pervasive Siberian-related gene flow that seems to have characterised European wolf history throughout the late Pleistocene thus appears to have ceased at least by 13 kya and remained absent throughout the Holocene.

- **China and central Asia**: The best-fitting models for present-day Chinese wolves all involve both the Siberian (*Siberia_BelayaGora.IN18_005*) and European (*Germany_HohleFels.JK2179*) post-LGM sources. The proportions of ancestry assigned to the European-related sources varies between ~20% and 90%, with higher levels in wolves in western China (Xinjiang, Qinghai, Inner Mongolia). This is consistent with the PCA analyses in which Chinese wolves fall on a cline between late Pleistocene Siberian and West Eurasian wolves (**Fig. 4a**). A present-day wolf from the Altai mountains is similarly modelled as 80% European-related and 20% Siberian-related. A 5k-year old wolf from Kazakhstan is modelled as 100% European-related (it also clusters with European wolves in the PCA). One hypothesis is that there was an eastward spread of European-related wolf ancestry into central and eastern Eurasia, including China, during the Holocene, though late Pleistocene wolf genomes from China and central Asia are required to test this. If this is the case, Chinese wolves predating this dispersal are expected to be less European-related and more Siberian-related.

  In addition to these two sources of ancestry, most Chinese wolves also require smaller amounts of a third, unsampled component of ancestry. The largest amounts (up to 27%) are estimated in wolves in Tibet and Inner Mongolia. This likely reflects the persistence of deep, local ancestry that is more divergent than both the ancient European and Siberian wolves available here, and which was not completely replaced by the Late Pleistocene ancestry homogenization. These results likely capture the same deep ancestry that has previously been described for wolves in Tibet and the Himalayas[7], and which might derive from an unknown, extinct canid lineage. These populations also carry mitochondrial lineages that are highly diverged from other wolf mitochondria[8,9,12], and

these were plausibly inherited from this unknown source. Largely based on these mitochondrial lineages, it has been proposed that wolf populations in Tibet and the Himalayas are distinct from other Eurasian wolves. Our results show that, while they carry a minority component of ancestry that is divergent, most of their ancestry is still connected to and shared with other Eurasian wolves within the timeframe of the last ~25,000 years.

- **The Near East and South Asia**: The five analysed present-day wolf genomes from the Near East and South Asia are modelled as 88-100% related to the 13k-year old European wolf *Germany_HohleFels.JK2179*, with the remainder being unsampled, divergent ancestry. This unsampled ancestry could reflect local wolf ancestry that has persisted in the face of ancestry homogenization, and/or admixture from other canid species such as the African golden wolf[6]. Like for European wolves, wolves in the Near East and South Asia do not seem to have received any Siberian-related gene flow since the onset of the Holocene.

- **North America**: All analysed Holocene and present-day North American wolves are well-modelled as a combination of ancestry related to the 18k-year old *Siberia_BelayaGora.IN18_005* and a present-day coyote. An exception is a 15k-year old wolf from the North Slope region of Alaska, which fits without the need for coyote ancestry (and it clusters with contemporaneous Siberian wolves in the PCA (**Extended Data Fig. 2**)), suggesting it might be a relatively recent migrant from Siberia that had not yet picked up coyote ancestry. All other analysed North American wolves are estimated to have at least a few percent coyote ancestry, including those in the Canadian Arctic and Greenland.

  These results imply that all of the wolf-related ancestry in the present-day North American wolves analysed here derives from post-LGM populations in Siberia, without any persistence of deep, local North American wolf ancestry. These models include the 29.9k-year old wolf *Yukon_QuartzCreek.SC19.MCJ010* in the reference set, thereby explicitly testing for and rejecting any contribution from populations related to this pre-LGM North American individual. These *qpAdm* results are consistent with the admixture graph analyses, where a graph without persistence of ancestry from earlier wolves in Alaska and the Yukon fits the data (**Fig. 2a**).

  The same two-source Siberia + coyote models also fit well for the four red and Algonquin wolves analyzed here, and thus are consistent with the model that they are the product of admixture between wolves and coyotes[13,14]. Like for North American grey wolves, the results imply that all of the wolf-related component of their ancestry is derived from post-LGM Siberian wolves, with no evidence for ancestry from any earlier wolf populations related to the older individuals in the *qpAdm* reference set, and that the admixture with the coyote-related component has occurred since the LGM. However, the results imply less about the history of the coyote-related component, and we cannot rule out that this component is in fact a composite of two or more ancestries with some degree of

divergence between them, but all in a clade with the ancestry represented by the *CoyoteCalifornia* individual used here. Ancient canid genomes from further into the North American continent, i.e. further east and south of Alaska and the Yukon, are necessary to characterise the history of the coyote-related ancestry in red wolves, Algonquin wolves and North American gray wolves, and how different they are from each other.

Persistence of deep, local ancestries

To further study the persistence of non-Siberian ancestries in Eurasian wolf populations, and to better understand the behaviour of *qpAdm* when the actual source populations are not available, we performed experiments in which we only included ancient Siberian wolves as *qpAdm* sources and reference populations. With ancient European wolves excluded, we can then observe how post-LGM and present-day European wolves are modelled. For this, we included (age estimate in parentheses, years BP):
Siberia_BelayaGora.IN18_005 (18,148)
Siberia_BelayaGora.IN18_016 (32,020)
Siberia_BungeToll.CGG29 (48,210)
Siberia_UlakhanSular.LOW008 (70,772)
Siberia_Enygen.VAL_18A (100,706)
Armenia_Hovk1.HOV4 (Ancient dhole)

The results revealed that for post-LGM and present-day European wolves, the single-source model involving the 18k-year Siberian wolf is strongly rejected, and a two-source model involving the outgroup species (dhole) is favoured (**Fig. S11**). The amount of ancestry assigned to the outgroup, or unsampled ancestry component, varies from ~10% to ~20%. This thus confirms the results obtained in the full *qpAdm* results, as well as in the admixture graph, that European ancestry is not completely replaced by Siberian ancestry. It also demonstrates that this conclusion can be reached even in the absence of genomes from the older European wolves that contributed this non-Siberian ancestry, and that the assignment of outgroup ancestry by *qpAdm* can reflect the persistence of deep, local ancestry.

All other analysed Eurasian wolves obtain similar results and are also assigned some amount of unsampled, divergent ancestry, with the exception of the post-LGM Siberian wolves which, as in the full *qpAdm* analyses described above, are well-fit by the single-source Siberian model. These results imply that most wolves outside of Europe also retain some amount of local, non-Siberian ancestry (though not necessarily the same local ancestry), and that populations in these regions were not completely genomically replaced by Siberian-related gene flow. As the results for European wolves demonstrate, this conclusion can be reached in the absence of ancient genomes from these regions (though of course, the identities of the local ancestries remain unknown). The largest amounts of unsampled ancestry are assigned to populations in Tibet and western China (~30-50%), as well as the Near East (~20%), consistent with other analyses indicating that these populations carry larger amounts of ancestry that is divergent from that of Siberian wolves.

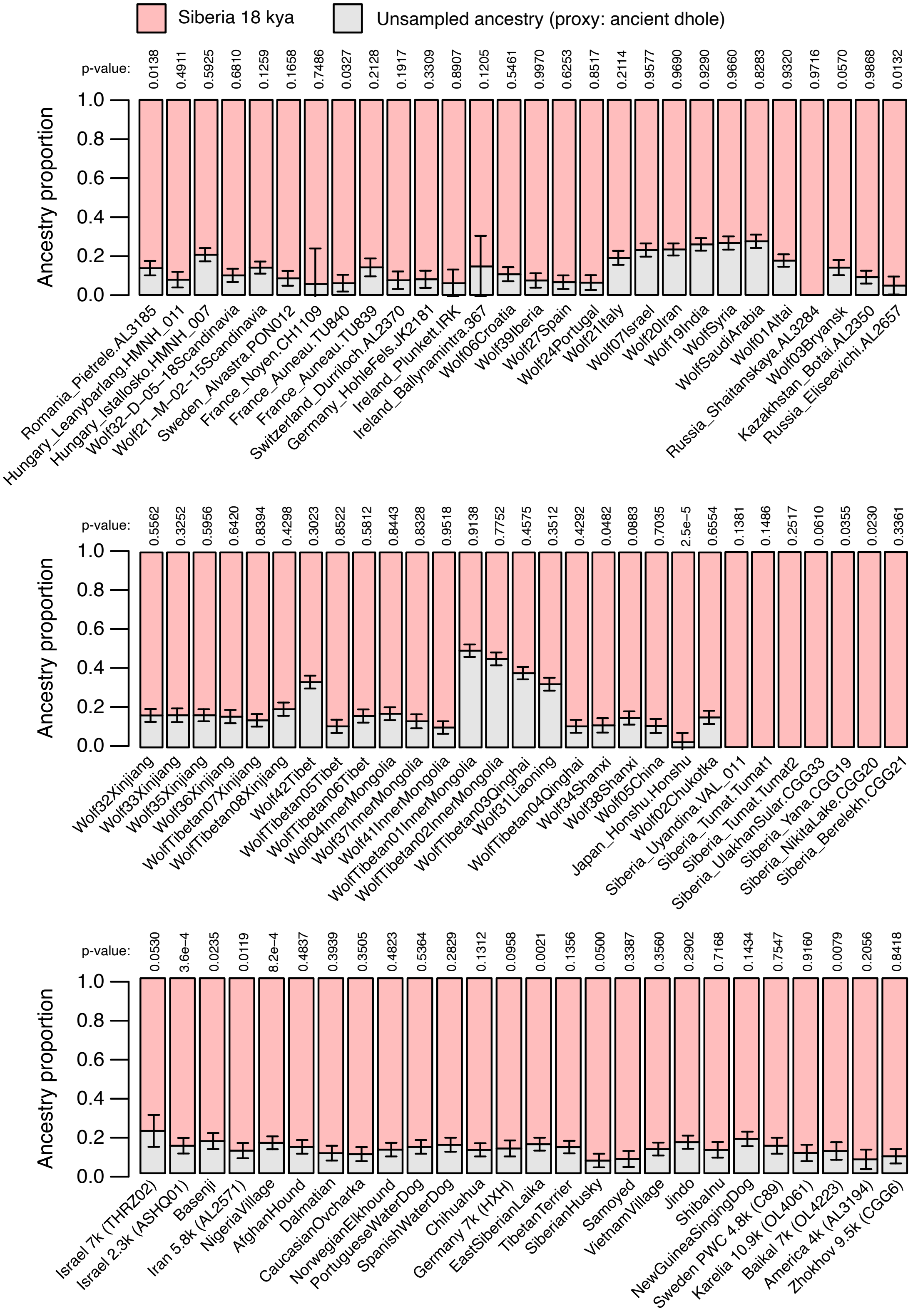

Fig. S11. *qpAdm* results with only Siberian sources reveal widespread non-Siberian ancestry. Best-fitting *qpAdm* models for post-LGM and present-day wolves and dogs, with only ancient Siberian wolves available as sources and reference populations. An ancient dhole genome was used to capture unsampled, divergent ancestry. Bars denote ±1 standard error estimated from a block jackknife.

The deep ancestry signal that *qpAdm* is picking up in these analyses should be visible directly in f_4-statistics, in particular in statistics of the form f_4*(Armenia_Hovk1.HOV4,Y; Siberia_BelayaGora.IN18_005,X)*, where *Siberia_BelayaGora.IN18_005* is the 18k-year old Siberian wolf, Y is some older Siberian wolf and X is some non-Siberian wolf. If X has some ancestry that is divergent from and not shared by Y and *Siberia_BelayaGora.IN18_005*, this will lead to a negative value of a statistic like this (that is, the old Siberian wolf Y is more similar to *Siberia_BelayaGora.IN18_005* than to X).

We investigated statistics of this form directly for different choices of older Siberian wolves Y, and indeed found negative values for most other wolves X living after the LGM (**Fig. S12**). This is thus consistent with most wolf populations outside of Siberia having some degree of ancestry that is divergent from the analysed Siberian wolves. Or in other words, that there is some degree of ancestry continuity over time among the Siberian wolves that is not fully shared by the non-Siberian wolves. Had there been a complete ancestry turnover at some point in the history of *Siberia_BelayaGora.IN18_005*, for example at the LGM, then all of these statistics would be expected to be 0, but that is not the case.

The negative signal is stronger the closer in time the older Siberian wolf Y is to the 18k-year old *Siberia_BelayaGora.IN18_005*, but negative values are obtained even when Y is set to be the ~100k-year old *Siberia_Enygen.VAL_18A*. This thus implies that part of the population structure that exists between Siberian and other wolves is at least 100k years old. Despite the dominant pattern in wolf relationships being strong connectivity and most ancestry being shared recently in time, a small part of wolf ancestries thus retain temporally very deep population structure.

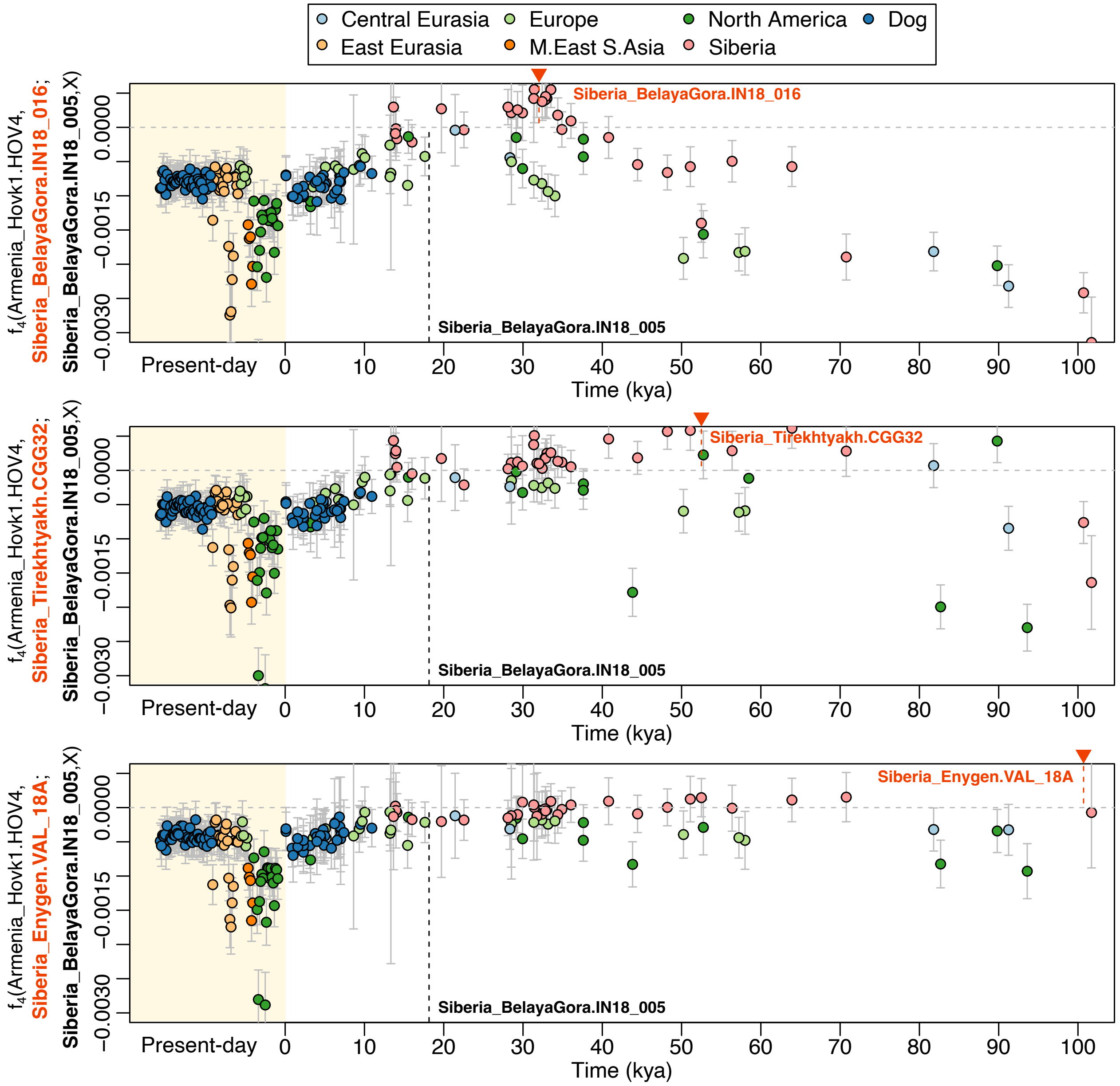

Fig. S12. Ancestry continuity in Siberia is not fully shared with other populations. f_4-statistics of the form f_4*(Armenia_Hovk1.HOV4 ,Y;Siberia_BelayaGora.IN18_005,X)*, where Armenia_Hovk1.HOV4 is an ancient dhole serving as an outgroup, Y is one of three ancient Siberian wolves that lived at different points prior to 30 kya (in orange, with dotted lines and arrowheads indicating their age), Siberia_BelayaGora.IN18_005 is an 18k-year old Siberian wolf (bold, with dotted lines indicating its age) and X are other individuals displayed in the plots. A negative value of this statistic implies that the old Siberian wolf is closer to the 18k-year old Siberian wolf than to X, meaning there is some deep ancestry in X that is not shared by the two Siberian wolves. All worldwide present-day wolves, as well as dogs, display negative values, suggesting the persistence of some amount of non-Siberian ancestry in these. Bars denote ±3 standard errors estimated from a block jackknife.

Genetic differentiation analyses (F_{ST})

Table S1. Sample pools used for pairwise F_{ST} calculations. The “age” column was used for plotting.

Pool name	Age	Individuals
Alaska_88k.pool2	88000	Alaska_Fairbanks.JAL65, Alaska_LillianCreek.ALAS_024
Germany_Aufhausener_55k.pool3	55000	Germany_Aufhausener.AH574, Germany_Aufhausener.AH575, Germany_Aufhausener.AH577
Siberia_50k.pool5	50000	Siberia_BungeToll.CGG29, Siberia_BungeToll.LOW003, Siberia_Tirekhtyakh.VAL_033, Siberia_Uyandina.VAL_012, Siberia_Yungen.DS04
Yukon_48k.pool2	48000	Yukon_ParadiseHill.SC19.MCJ015, Yukon_HunkerCreek.SC19.MCJ017
Alaska_38k.pool2	38000	Alaska_Fairbanks.JAL385, Alaska_Fairbanks.JAL69
Germany_HohleFels_33k.pool2	33000	Germany_HohleFels.JK2175, Germany_HohleFels.JK2183
Siberia_Indigirka_33k.pool6	33000	Siberia_Badyarikha.VAL_008, Siberia_BelayaGora.IN18_016, Siberia_Letniaya.LOW002, Siberia_Ogorokha.VAL_005, Siberia_Ogorokha.VAL_050, Siberia_Tirekhtyakh.LOW006
Siberia_Yana_32k.pool5	32000	Siberia_Yana.CGG23, Siberia_Yana.CGG25, Siberia_Yana.CGG26, Siberia_Yana.CGG27, Siberia_Yana.CGG28
Yukon_30k.pool2	30000	Yukon_QuartzCreek.SC19.MCJ010, Yukon_IndependenceCreek.SC19.MCJ014
Siberia_Indigirka_29k.pool2	29000	Siberia_Badyarikha.CGG34, Siberia_Tirekhtyakh.LOW007
Siberia_Indigirka_20k.pool2	20000	Siberia_Uyandina.VAL_011, Siberia_BelayaGora.IN18_005
Germany_HohleFels_14k.pool2	14000	Germany_HohleFels.JK2179, Germany_HohleFels.JK2181
Siberia_BerelekhNikita_14k.pool2	14000	Siberia_Berelekh.CGG21, Siberia_NikitaLake.CGG20
Siberia_Tumat_14k.pool2	14000	Siberia_Tumat.Tumat1, Siberia_Tumat.Tumat2
Ireland_13k.pool2	13000	Ireland_Ballynamintra.367, Ireland_Plunkett.IRK
France_9k.pool2	9000	France_Auneau.TU840, France_Noyen.CH1109
Canada_Umingmak_3k.pool2	3000	Canada_Umingmak.TU144, Canada_Umingmak.TU148
Wolf_Algonquin.pool2	0	AlgonquinWolf13467, AlgonquinWolf13470
Wolf_Baffin.pool2	0	WolfBaffinNorth, WolfBaffinSouth
Wolf_Iberia.pool3	0	Wolf24Portugal, Wolf27Spain, Wolf39Iberia
Wolf_InnerMongolia.pool5	0	Wolf04InnerMongolia, Wolf37InnerMongolia, Wolf41InnerMongolia, WolfTibetan01InnerMongolia, WolfTibetan02InnerMongolia
Wolf_Levant.pool2	0	Wolf07Israel, WolfSyria
Wolf_Mexico.pool2	0	Wolf22Mexico, Wolf23Mexico
Wolf_Qinghai.pool2	0	WolfTibetan03QinghaiHighland, WolfTibetan04QinghaiHighland
Wolf_Scandinavia.pool12	0	Wolf21-M-02-15Scandinavia, Wolf32-D-05-18Scandinavia, Wolf33-M-05-01Scandinavia, Wolf42-M-07-02Scandinavia, Wolf63-M-10-10Scandinavia, Wolf71-G82-10Scandinavia, Wolf79-M-09-03Scandinavia, Wolf7-D-77-01Scandinavia, Wolf82-G23-13Scandinavia, Wolf83-G31-13Scandinavia, Wolf8-D-79-01Scandinavia, WolfD-85-01Scandinavia
Wolf_Shanxi.pool2	0	Wolf34Shanxi, Wolf38Shanxi
Wolf_Tibet.pool3	0	Wolf42Tibet, WolfTibetan05Tibet, WolfTibetan06Tibet
Wolf_Xinjiang.pool6	0	Wolf32Xinjiang, Wolf33Xinjiang, Wolf35Xinjiang, Wolf36Xinjiang, WolfTibetan07Xinjiang, WolfTibetan08Xinjiang
Wolf_Yellowstone.pool2	0	Wolf28Yellowstone, Wolf29Yellowstone

SI section 3: MSMC2 analyses of N_e history and divergence times

Data processing for MSMC2 analyses

For MSMC2 analyses, we included six ancient wolf genomes that had sufficiently high coverage to enable the calling of diploid genotypes (age estimate in parentheses, years BP, followed by x-fold coverage): *Siberia_Tirekhtyakh.CGG32* (52,500, 15.1x), *Siberia_BungeToll.CGG29* (48,210, 6.3x), *Siberia_BungeToll.LOW003* (44,450, 5.9x), *Germany_HohleFels.JK2183* (32,366, 7.8x), *Siberia_BelayaGora.IN18_016* (32,020, 13.0x), *Siberia_UlakhanSular.CGG33* (16,864, 15.4x). We also included the ancient dogs *Zhokhov.CGG66*[15] (9,500, 9.2x) and *Ireland_Neolithic.Newgrange*[16] (4,800, 30.7x). We included a number of previously published present-day wolf genomes, processed as described in the Methods section on Illumina read processing. We also included four present-day dog genomes obtained as read alignments from the DoGSD database[17] (x-fold coverage in parentheses): GS2, German Shepherd (16.0x), Dog08, Chow Chow (6.7x), DQ10, village dog Diqing, China (16.0x) and Basenji (5.8x).

We called genotypes using HaplotypeCaller from the Genome Analysis Toolkit (GATK) v3.6[18], applying the "--sample_ploidy 1" argument to HaplotypeCaller when calling male X chromosomes, and the "--includeNonVariantSites" argument to the GenotypeGVCFs command to obtain genotype calls at all sites. The calls were then filtered by setting any genotype to missing if the number of reads at the site was less than half, or greater than double, the genome-wide average coverage for the given sample. Indel variants were also excluded. MSMC2 "multihetsep" input files were then generated using the included generate_multihetsep.py script. A mappability map was also applied at this stage, generated using the gem-mappability program[19] v1.315 using a kmer length of 35. We ran MSMC2[20] version 2.1.1 with default parameter settings unless otherwise noted.

We scaled all MSMC2 results using a mutation rate of $0.4 \cdot 10^{-8}$ per site per generation[3,21], and a mean generational interval of 3 years[3]. However, we note that there is uncertainty surrounding both of these parameters, and that if either of them are inaccurate this would mean that our results will be biased. For example, it has been suggested that a generational interval in wolves of three years is too short, and that a value of 4 or more would be more accurate[22] - applying a larger value would lead to deeper inferred divergence times. For analyses restricted to the X chromosome, we scaled down the mutation rate by 25%, to account for the lower mutation rate of this chromosome relative to the autosomes (this scaling factor estimate was derived from humans[23], and we made the assumption that it is similar in wolves). When plotting curves obtained from a single diploid, ancient genome, we shifted back the curve in time by the age of that genome. When plotting curves obtained on two male X chromosomes from individuals of different ages, we shifted the curves back by an amount of time corresponding to the midpoint between their ages, to account for how the rate of mutations accumulated between the two genomes will be halved after one of them has died.

Population size inferences

To enable population size (N_e) inferences also on ancient wolf genomes, we applied an approach aiming to circumvent the potentially confounding effects that apparent transition variants caused by ancient DNA damage can have on such analyses. Since MSMC2 uses all callable sites, not just those that are variable, simply removing transition variants would lead to large underestimation of the divergences between chromosomes, and therefore skew the inferences of effective population sizes. Instead, we ignored transition variants by treating them as monomorphic, and then scaled the inferences by applying a transversions-only mutation rate.

A transversions-only mutation rate could be inferred in various ways - here we did this as follows. For each of 24 present-day wolf genomes, which do not have ancient DNA damage, we ran MSMC2 once with all variants included and once with transition variants set to monomorphic. We then searched, through systematic testing between 0 and 1 in increments of 0.001, for the mutation rate scaling factor that minimised the sum of squared euclidean distances between the all-mutations and transversions-only curve (ignoring time segments falling outside the interval 10^4 - 10^6 years in the all-mutations curve). Across the 24 genomes, the average of these scaling factors was 0.308375 (standard deviation: 0.0213). This agrees with the general expectation that transversions constitute roughly one third of all variants, i.e. a transition to transversion ratio of about 2 (e.g. the human 1000 Genomes Project found a ratio of 1.98[24]). Applying this inferred scaling factor to the mutation rate, we can thus scale MSMC2 curves that have been inferred on genomes in which transitions have been ignored.

We ran MSMC2 with the argument "--timeSegmentPattern 25*1+1*2+1*3" to enable higher resolution in the more recent past. We found that the curves for the ancient genomes displayed more noisy behaviour in the recent past, and so for them we used default values for this time pattern parameter ("1*2+25*1+1*2+1*3"). The curve for the sample Siberia_BungeToll.LOW003, which had the lowest sequence coverage out of the included genomes, displayed a sharp increase in N_e in recent time despite ignoring transition and so was excluded from this analysis (it behaved well in the X chromosome divergence analyses, however).

The results (**Fig. S13**) on present-day wolves are similar to previously published results[25–27], and the curves from most individuals displayed a decline in N_e in the last ~30,000 years. Some individuals displayed less of this, including Wolf19India, Wolf20Iran, WolfTibetan08Xinjiang, Wolf35Xinjiang, though this could reflect either that N_e remained high in the history of these individuals, or that they have experienced admixture from other populations more recently. The ancient wolf genomes display curves that are similar to those inferred from modern genomes, but without the recent decline. This implies that these individuals lived before most of the N_e reduction occurred. In general, all estimates are somewhat noisy in the most recent time segments (e.g. the last 10,000 years), reflecting the lack of resolution in recent times when analysing just one genome.

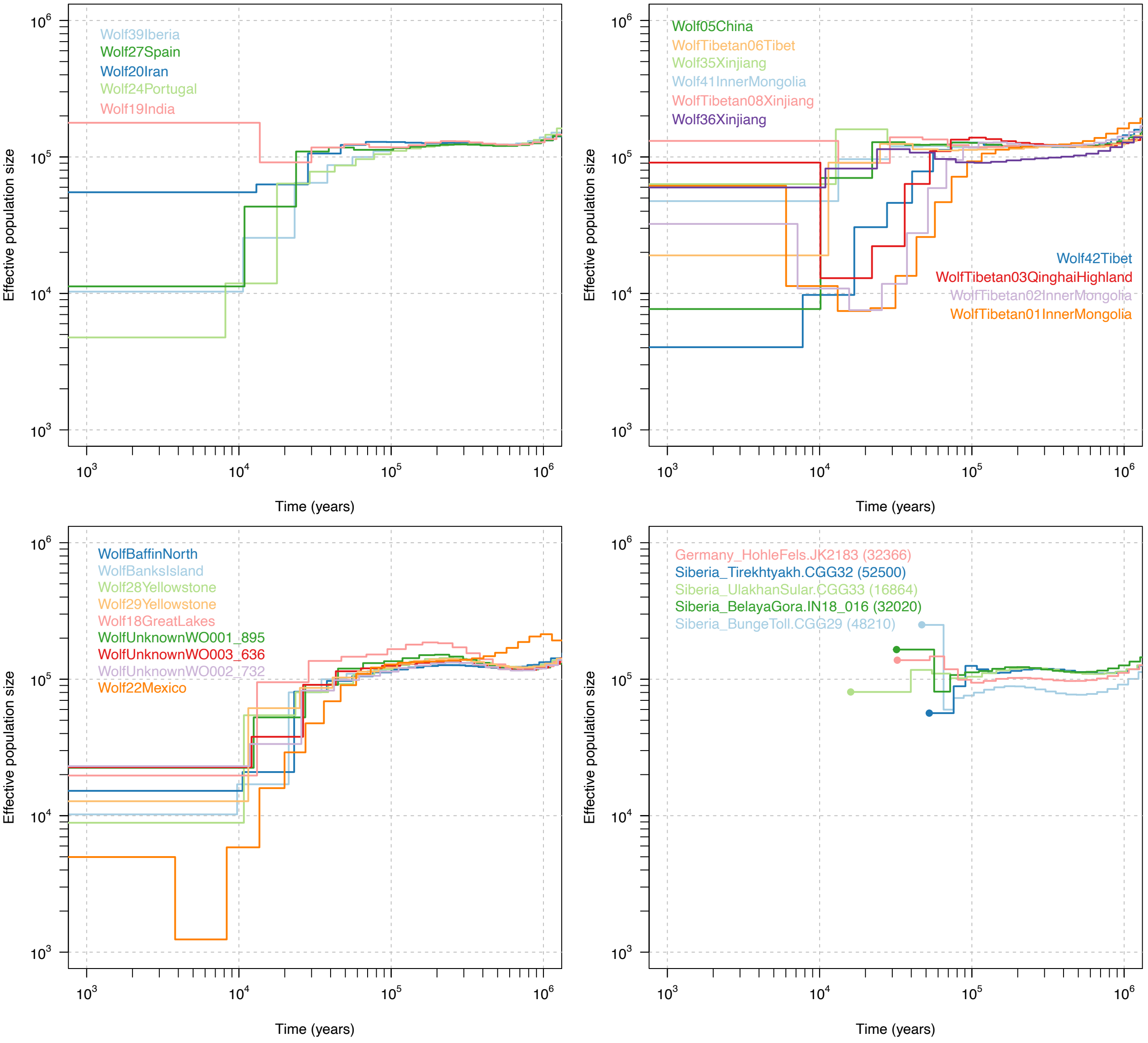

Fig. S13. MSMC2 analyses of past effective population sizes. Transition variants were set to be monomorphic, and the results were scaled using a transversions-only mutation rate. The curves for ancient genomes (bottom right) are shifted along the temporal axis according to their age.

Divergence time inferences

We used MSMC2 on pairs of haploid X chromosomes from two different male genomes to estimate divergence times between populations. Performing effective population size (N_e) inference on such a 'pseudo-diploid' chromosome is expected to result in an extremely large inferred effective population size since the point in time at which the two populations became genetically separated, reflecting the lack of coalescence events that are more shallow than that [28]. The point at which a pseudo-diploid N_e curve turns sharply upwards can thus be taken as an approximation of the population divergence time. For wolf-wolf and wolf-dog analyses, we heuristically called divergence times from the curves by identifying the most recent segment for which the estimated N_e exceeds a given threshold (80,000) and which had at least 10% higher N_e than the next, earlier segment. These thresholds were arbitrarily chosen after visual inspection of the curves, and the point estimates resulting from this are not necessarily expected to accurately capture the timing of the separation process as well as a visualisation of the full curve.

We first applied this MSMC2 methodology to the question of the divergence times between wolves and coyotes. We ran the CoyoteCalifornia individual against a set of diverse present-day and ancient wolves and dogs. The curves for most of these individuals look very similar, and show an increase in N_e starting from about ~700 kya (**Fig. S14**). However, the shape of the curve is quite gradual, making it difficult to infer a precise divergence time. It is possible that this partly reflects a genuinely gradual separation process, in which case a single point in time could not appropriately describe that process. In any case, the results show clear genetic separation between wolves and coyotes, at least since ~100 kya (consistent with all the ancient wolf genomes sequenced here being clearly identifiable as wolves, rather than from a lineage ancestral to wolves and coyotes) and perhaps starting ~700 kya. Two present-day individuals, Wolf18GreatLakes and the red wolf Wolf25Red show very different curves indicating shared ancestry with coyote at substantially more recent times scales, reflecting the coyote-related ancestry in these North American canids.

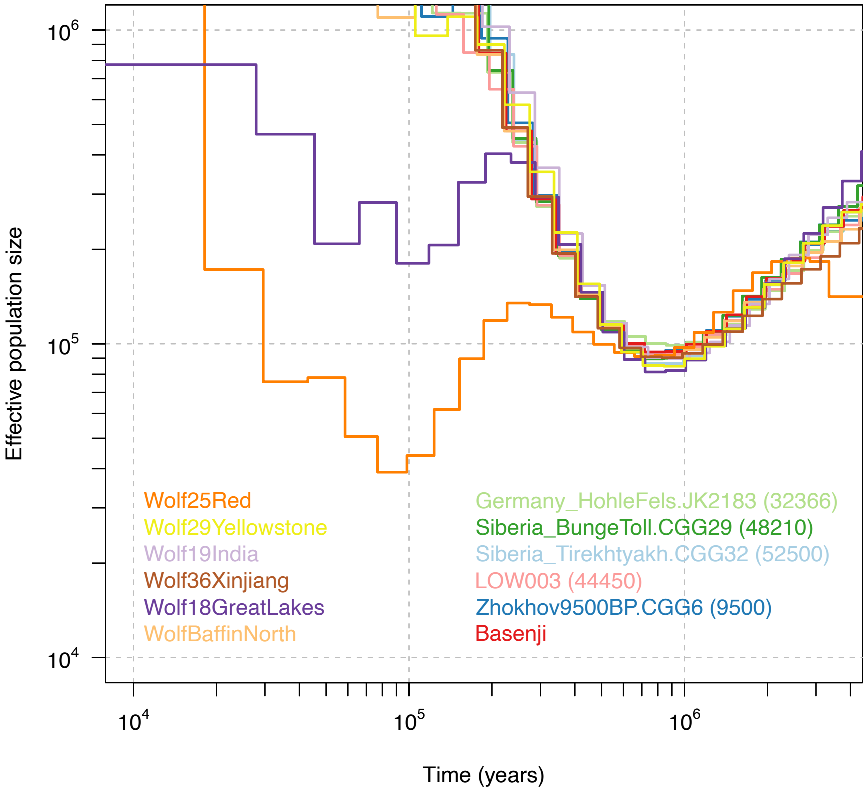

Fig. S14. MSMC2 analyses of wolf-versus-coyote divergence times on pairs of haploid male X chromosomes. The CoyoteCalifornia (Coyote01) individual was used in all comparisons, against a selection of present-day and ancient wolf and dog genomes. An increase in inferred effective population size starting from ~700 kya suggests that genetic separation between wolves and coyotes began around this time, but the gradual shape of the curve makes it difficult to infer a precise divergence time. Two North American individuals (Wolf25Red and Wolf18GreatLakes) display more recently shared ancestry with the coyote, reflecting the substantial coyote-related ancestry in these individuals.

We also applied this MSMC2 chrX methodology to pairs of wolves, involving both ancient and present-day individuals (**Fig. S15**). We found that when running the inference on two genomes that are substantially separated in time (whether one present-day and one ancient, or two ancient genomes) the inferred divergence time always tends to fall close to the age of the older of the two genomes. For example, the divergence times between the 32k-year old European wolf JK2183 and the present-day wolves Wolf19India, Wolf36Xinjiang and Wolf39Yellowstone are inferred to be 32.8, 30.9 and 32.3k years, respectively. Similarly, the divergence time between the 32k year old European wolf JK2183 and the 52.5-year old Siberian wolf CGG32 is inferred to be 58.4k years, just a few thousand years older than the age of CGG32. In some cases, no sudden increase in inferred N_e is observed, which implies that any separation between the two populations from which the X chromosomes are sampled is too recent to be detectable with this method. For example, this occurs between the two ancient genomes CGG32 and LOW003, both of which lived in Siberia 52.5 and 44.5 kya, respectively. There must necessarily be a few thousand years of divergence between them given their separation in time,

but a divergence this shallow is likely beyond the resolution of this method. In these cases, in the summary in **Fig. 2d** the split time is set to the age of the oldest genome.

An interpretation of these results is as follows. The divergence time between two genomes can be separated into two components: 1) the time that passed since the two populations separated genetically, until an individual from one of them dies and becomes an ancient DNA sample. 2) the time since the death of the first individual, until the death or sampling of the second individual. The second of these components is necessarily equal to the age difference between the two samples, and it follows that an estimated divergence time must be at least this large. Any additional divergence beyond this age difference must then be attributed to the first of these divergence components, the population divergence. The results obtained here thus imply that the population divergences among wolves are very shallow, as there is little detectable divergence beyond the age differences between genomes. In other words, that the population represented by an older wolf genome A, and the (likely unsampled) population that is ancestral to the younger wolf genome B and was contemporaneous to A, share much of their ancestry within the preceding ~10k years (the method likely does not allow for higher resolution assessments than that). To a first approximation, older wolf genomes sampled here can thus be thought of as, if not the actual ancestors (it is unlikely that we have sampled actual genetic ancestors), then as closely genetically related to the ancestors of younger wolves.

We also applied this MSMC2 chrX methodology to pairs of one wolf and one dog, as a way to potentially date the timing of the divergence of the dog lineage from the wolf populations represented by available ancient and present-day wolf genomes (**Fig. S16**). When analysed against the 32k-year old ancient wolf and older ancient wolves, the results involving dogs are very similar to those of present-day wolves: the inferred divergence times are close to the age of the older wolf. This implies that the lineage that became the ancestors of dogs had yet to diverge substantially from the 32k-year old wolf, and likely were still strongly genetically connected to other wolf populations at the time that this wolf lived.

When analysed against present-day wolves from India, China and North America, the divergence times of dogs fall largely between 18 and 30 kya. This timeframe therefore serves as an estimate of when the lineage leading to dogs separated genetically from the ancestors of these analysed present-day wolves. However, this is not an estimate based directly on ancient genomes and is therefore subject to the various caveats that apply to inferring the deep past on the basis of present-day diversity, including: modelling assumptions built into the MSMC2 method, the assumed mutation rate and generation time used to scale the results, and possibly later population processes (e.g. admixture) that might have affected the relationships between the analysed genomes since their divergence.

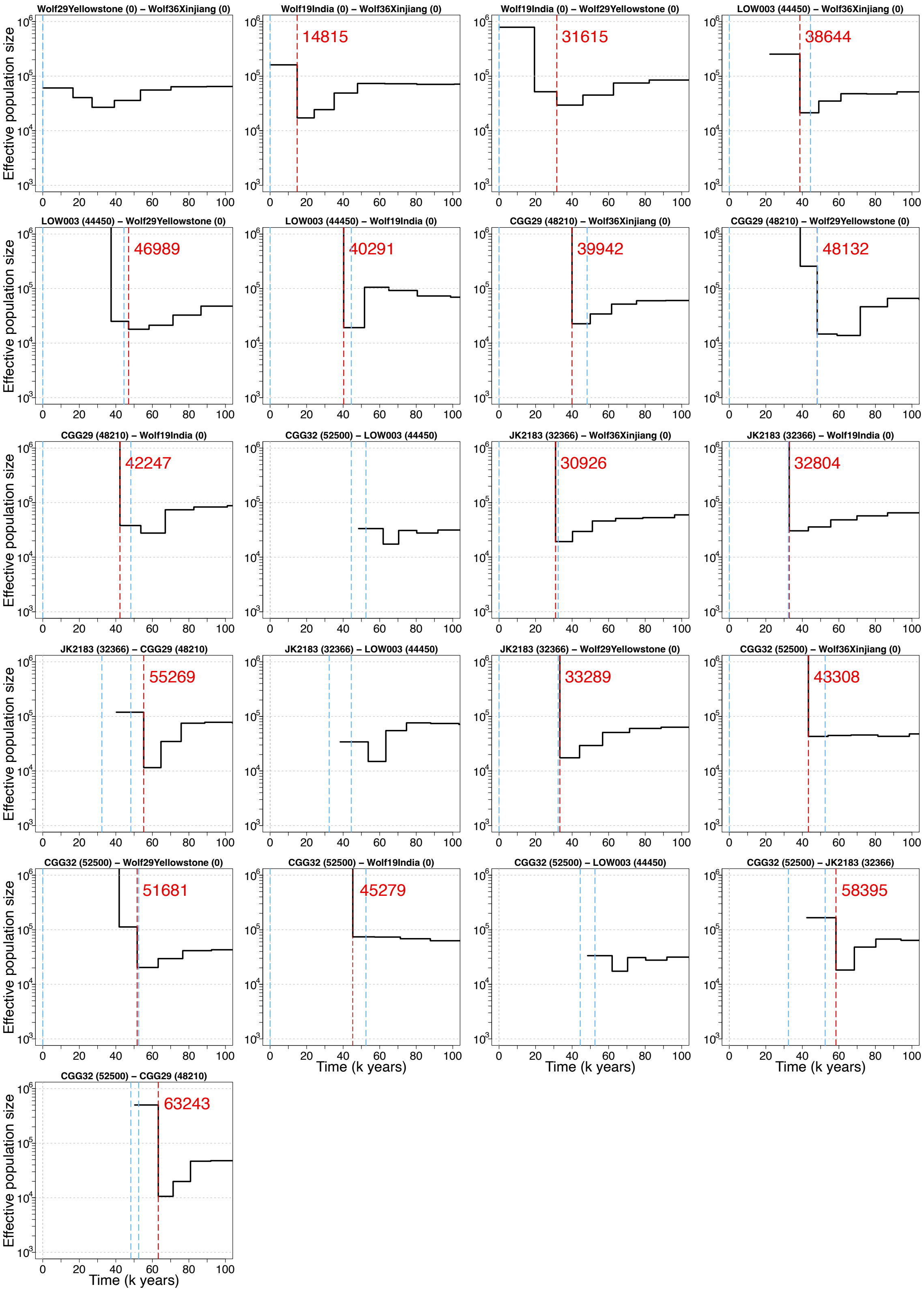

Fig. S15. MSMC2 analyses of wolf-versus-wolf divergence times on pairs of haploid male X chromosomes. The ages of the individuals in each pair are indicated with dotted, blue lines. The heuristically inferred divergence time is indicated with a dotted, red line. If no substantial spike is observed, no divergence time is inferred.

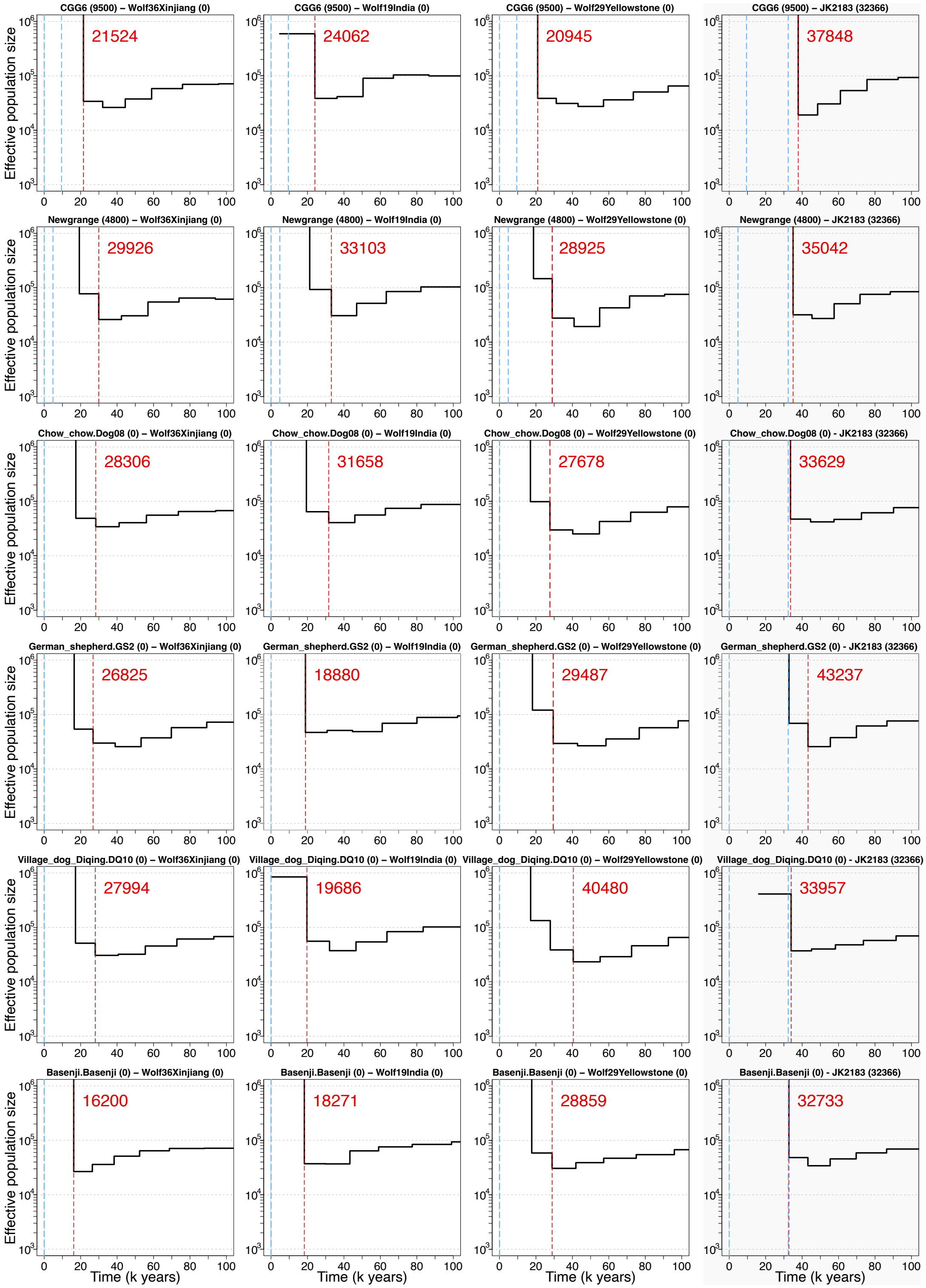

Fig. S16. MSMC2 analyses of dog-vs-wolf divergence times on pairs of haploid male X chromosomes. The ages of the individuals in each pair are indicated with dotted, blue lines. The heuristically inferred divergence time is indicated with a dotted, red line. The rightmost column displays results for dogs versus a 32k-year old wolf, for which little if any divergence deeper than the age of that wolf is detectable.

SI section 4: Natural selection analyses

Neutral simulations to assess the robustness of the selection scan

To understand whether the temporal allele frequency changes that we observe in the selection scan could possibly be the result of genetic drift alone, and to what extent our analysis is able to correct for genetic drift, we applied the same analysis to simulated, neutral populations. We used the ms program[29] to generate coalescent simulations of panmictic populations evolving in the absence of natural selection and with constant size. We applied a mutation rate of $0.4 \cdot 10^{-8}$ per site per generation[3,21] and a recombination rate of $1 \cdot 10^{-8}$ per site per generation (which is similar to empirical estimates of the dog recombination rate, i.e. ~1 cM/Mb or a total map length of ~2000 cM[30,31]). 500 chromosomes of size 5 Mb were simulated, resulting in an amount of sequence approximately as large as the wolf genome. Four separate populations were simulated, with effective population sizes (N_e) of 10,000, 25,000, 50,000 and 100,000, respectively.

We aimed for the genomes drawn from the simulations to match the empirical genomes as closely as possible in terms of sample ages and data missingness. We used the standard ms command line argument to sample 134 haplotypes from the present-day population, that is the end-point of the simulation, and paired these up to form 67 modern, diploid genomes which were arbitrarily matched up with the 67 modern wolves used in the empirical analysis. We used the "-eA" ms argument to sample ancient individuals from the simulation, applying one argument matching the age of each of the 73 ancient wolves (including the historical Japanese wolf) used in the empirical analysis (assuming a generation time of 3 years[3]), sampling two chromosomes at each of these time points. The genotypes of the ancient individuals were turned homozygous by randomly choosing one of the two alleles, so as to match the pseudohaploid nature of the empirical ancient data. Genotype missingness was then introduced into these 67 modern and 73 ancient simulated genomes, by randomly setting each genotype to missing according to a probability equal to the fraction of missing genotypes in their matched, empirical genomes. We also sampled an "outgroup" genome at 1 million years ago, which was used when calculating f_4-statistics on the simulated data (**Fig. S5**), but was not used in the selection analyses.

The ms argument thus looked like this, here for the N_e = 10,000 population:

```
ms 134 1 -a -p 12 -seeds $RANDOM $RANDOM $RANDOM -t 800 -r 2000 5000000 -eA 0.00166667 1 2 -eA
0.0266668 1 2 -eA 0.0266668 1 2 -eA 0.029375 1 2 -eA 0.042325 1 2 -eA 0.043075 1 2 -eA 0.0525583 1 2 -
eA 0.0559917 1 2 -eA 0.0719082 1 2 -eA 0.0779 1 2 -eA 0.0809668 1 2 -eA 0.0837917 1 2 -eA 0.110158 1 2
-eA 0.110242 1 2 -eA 0.111117 1 2 -eA 0.113833 1 2 -eA 0.116458 1 2 -eA 0.117683 1 2 -eA 0.117683 1 2 -
eA 0.128725 1 2 -eA 0.129517 1 2 -eA 0.140533 1 2 -eA 0.146942 1 2 -eA 0.151233 1 2 -eA 0.16425 1 2 -eA
0.178625 1 2 -eA 0.187942 1 2 -eA 0.23425 1 2 -eA 0.236208 1 2 -eA 0.237667 1 2 -eA 0.237842 1 2 -eA
0.242858 1 2 -eA 0.244292 1 2 -eA 0.249458 1 2 -eA 0.249525 1 2 -eA 0.261257 1 2 -eA 0.261368 1 2 -eA
0.261868 1 2 -eA 0.26465 1 2 -eA 0.266832 1 2 -eA 0.269717 1 2 -eA 0.270157 1 2 -eA 0.273175 1 2 -eA
0.2737 1 2 -eA 0.275168 1 2 -eA 0.276357 1 2 -eA 0.279292 1 2 -eA 0.283582 1 2 -eA 0.286525 1 2 -eA
0.29085 1 2 -eA 0.300418 1 2 -eA 0.313368 1 2 -eA 0.313468 1 2 -eA 0.340007 1 2 -eA 0.365175 1 2 -eA
0.370418 1 2 -eA 0.40175 1 2 -eA 0.41865 1 2 -eA 0.42585 1 2 -eA 0.4375 1 2 -eA 0.439543 1 2 -eA 0.4698
1 2 -eA 0.476942 1 2 -eA 0.48335 1 2 -eA 0.532682 1 2 -eA 0.589768 1 2 -eA 0.681608 1 2 -eA 0.68895 1 2
-eA 0.748468 1 2 -eA 0.760417 1 2 -eA 0.780018 1 2 -eA 0.839217 1 2 -eA 0.84755 1 2 -eA 8.33333 1 2
```

In this way we obtained simulated datasets from panmictic, neutrally evolving populations, with data properties that are very similar to the empirical wolf genome dataset. We then applied the same PLINK linear association analysis and filtering to these datasets as we had applied to the empirical data. The results show that no variants in these neutral simulations reach the genome-wide significance threshold, i.e. there are no false positives (**Fig. S17**). QQ-plots comparing the observed p-values to those expected under a uniform distribution also show no inflation of low p-values in the results from the simulations, in contrast to the results obtained on the empirical data (**Fig. 3b**).

An important part of why this analysis avoids false positives is the application of genomic control. Genomic control was developed to correct for population structure in genome-wide association studies[32]. In these panmictic simulated populations there is no geographical structure, but there is strong temporal structure. Genetic drift leads to changes in allele frequencies across the whole genome, and the magnitude of these changes depends on the effective population size - allele frequencies will change faster in a smaller population. By capturing the genome-wide inflation in the test statistics associating allele frequency to time, genomic control thus serves to correct for the changes expected from drift alone. The λ inflation factor estimated through genomic control is larger in the simulations with small effective population size, reflecting how in these simulations there is a larger amount of genetic drift to correct for (**Fig. S17**). In the simulation with an effective population size of only 10,000, many variants change in frequency as rapidly as the top variants in our empirical scan, and would have highly significant p-values if not corrected - but the application of genomic control shows that these variants are not unexpected given the magnitude of allele frequency changes genome-wide. The empirical results (**Fig. 3a,b**), where $\lambda = 1.61475$, effectively tell us that wolf population sizes must have been substantially larger than 10,000 during the time periods relevant to the selection scan, and that the observed outlier peaks cannot be explained by genetic drift alone.

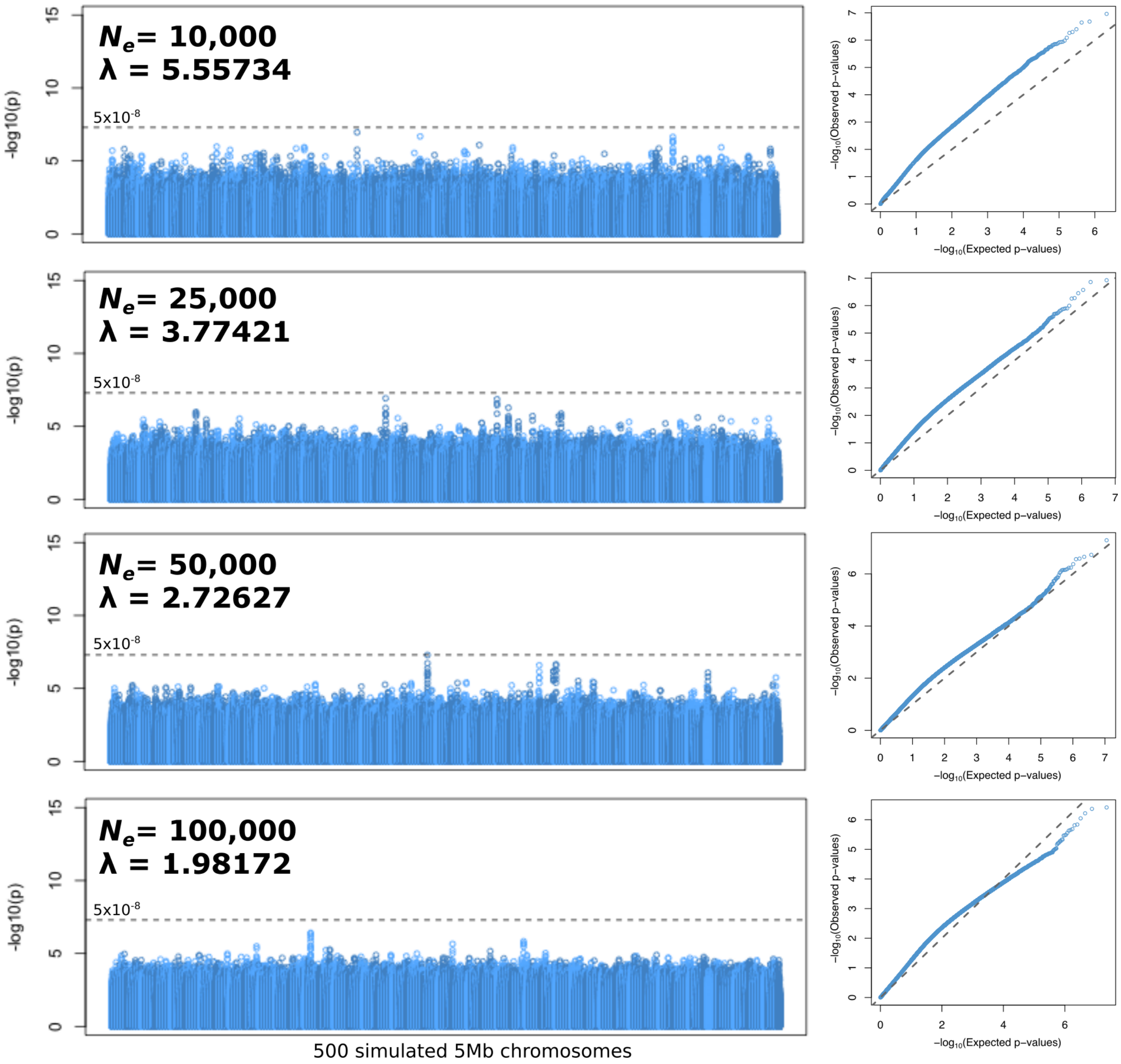

Fig. S17. Selection scans on simulated neutral populations. Manhattan plots of -log10(p-values) from linear regression (two-sided, not adjusted for multiple comparisons) for selection scans performed on four simulated, panmictic populations with different effective population sizes (N_e). The λ value displayed for each plot is the inflation factor estimated through genomic control. Corresponding QQ plots are displayed to the right of each manhattan plot.

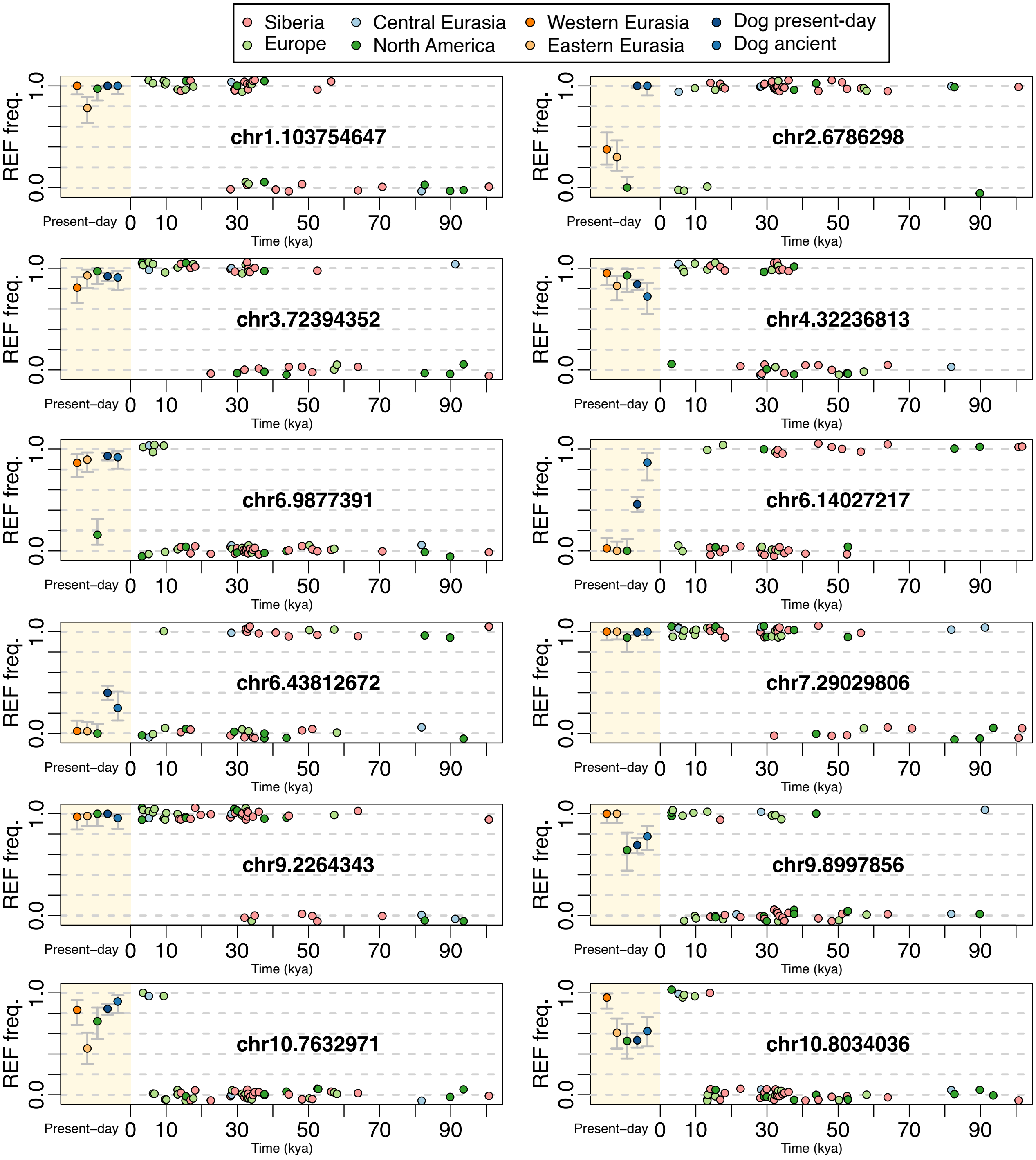

Siberia
Europe
Central Eurasia
North America
Western Eurasia
Eastern Eurasia
Dog present-day
Dog ancient
REF freq.
1.0
0.0
Present-day
0
10
30
50
70
90
Time (kya)
chr1.103754647
chr2.6786298
chr3.72394352
chr4.32236813
chr6.9877391
chr6.14027217
chr6.43812672
chr7.29029806
chr9.2264343
chr9.8997856
chr10.7632971
chr10.8034036

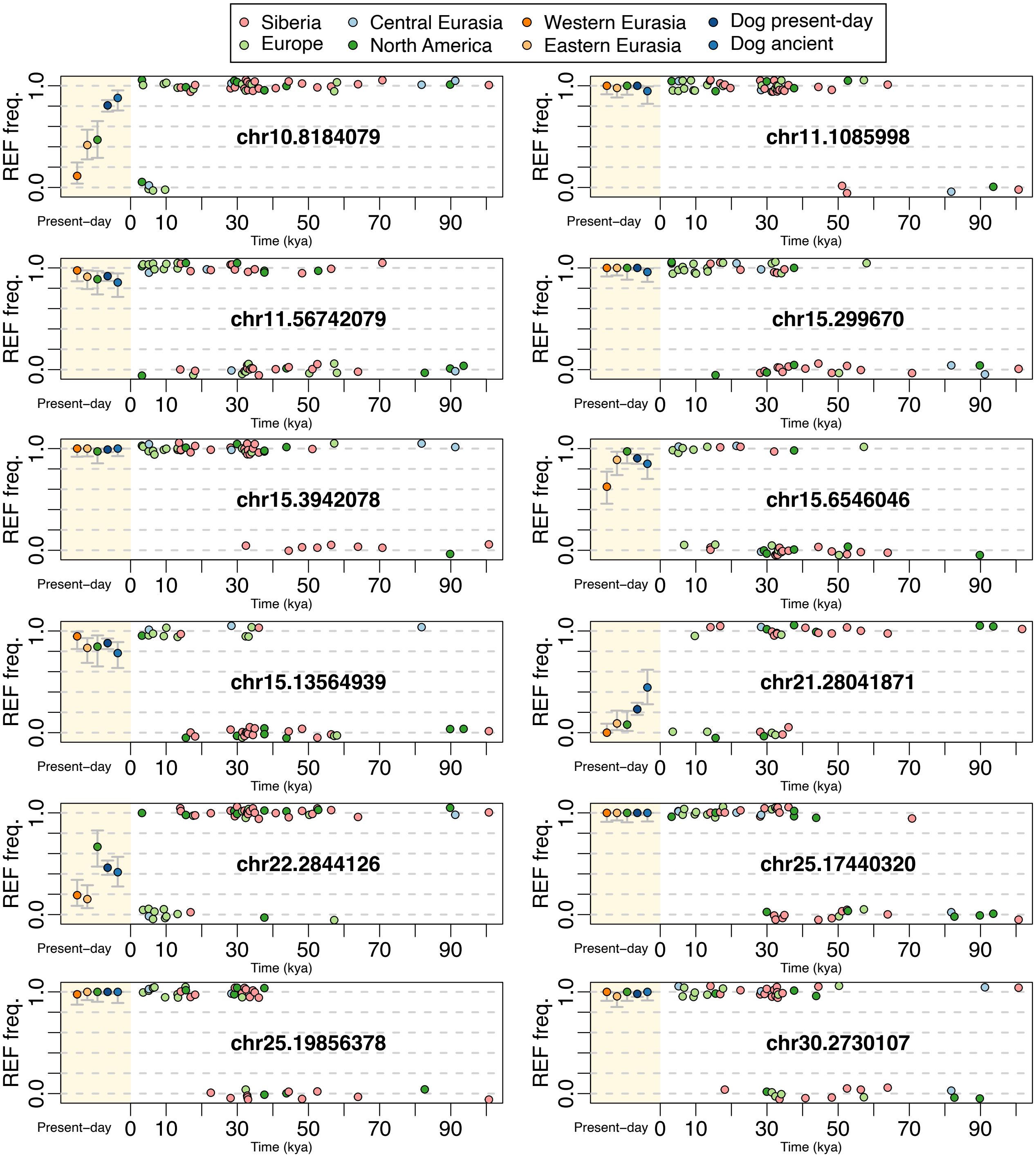

Fig. S18. Allele frequency over time at selective sweeps (two pages). The lead SNP from each peak is displayed, with genotypes for individual ancient wolves (with random vertical jitter), and allele frequencies averaged over present-day populations (ancient dogs are also plotted among the present-day populations to avoid clutter). Bars denote 95% binomial confidence intervals.

Inference of TMRCAs along the genome using Relate

Sample selection and data preparation

We initially selected all dogs and wolves from the 722g dataset[33] with coverage >8x, excluding the Andean fox, and filtered the data to exclude indels and non-biallelic SNPs and retained only sites with <1% of missing genotypes per site. We then used SHAPEIT4 v4.2.1[34] to phase the data without a reference panel. For downstream analyses using Relate[35], we selected 39 wolves, one coyote, and 95 dogs.

Relate requires an ancestral genome to correctly root inferred genealogical trees. To do so, we used the Andean fox as an outgroup, by generating a random fasta file from high coverage Andean fox data[36] mapped to the dog reference genome. Relate has been shown to be robust to occasional misspecification of ancestral alleles[35].

Another requirement in Relate is the filtering of genomic regions by whether they were callable, to adjust the local mutation rate in each local genealogical tree. This is necessary as, for instance, a lack of observed polymorphisms may be because the sample descends from a recent ancestor or because of missing data in that region.

We first applied a mappability mask to identify genomic regions where reads of length 35 bp are not uniquely mappable (the same mask that was used for the MSMC2 analyses, as described under "Data processing for MSMC2 analyses" in SI section 3). We additionally removed any sites with missing data in the Andean Fox, implying that a genomic ancestor was not callable. Finally, we applied a third filter, to account for the excluded polymorphisms with >1% missing data because the exclusion of these polymorphisms is expected to shorten TMRCAs and we wanted to make sure that this isn't driving our signal of selection on chromosome 25 (the strongest peak in the selection scan using ancient genomes). To account for this, we calculated the mean pairwise difference between the coyote and Andean fox in our data set as a rolling mean over a window size of 10 kB. We then required that the reduction in the mean pairwise difference after excluding polymorphisms with >1% missing genotypes was at most 50% and that the mean pairwise difference exceeded 0.005; the latter value was chosen to correspond to less than the 5th percentile of mean pairwise differences before filtering SNPs for missingness. As a reference, our region of interest on chromosome 25 only had a reduction in mean Andean fox-coyote difference of ~7%. The mappability mask excluded 11.5% across 38 autosomal chromosomes and our two additional filters excluded a further 4.6% of the genome.

Inference of genealogies using Relate and TMRCA calculation

We inferred genome-wide genealogies using Relate v1.1.8[35,37] using a per base per generation mutation rate of $0.4\cdot10^{-8}$ [21,38] and a recombination map downloaded from https://github.com/auton1/dog_recomb [36]. To rescale generations to years, we assumed a mean generational time of 3 years[38]. We initially ran Relate by setting the haploid effective population size to 200,000 and subsequently fitted a time-varying population size history, and branch lengths reflecting this population size history, using the EstimatePopulationSize.sh script provided with the Relate package. We used default parameters in this script but set option --th

0.8, to reduce the total number of trees used for fitting population sizes and therefore reducing computation time.

We then computed the time to the most-recent common ancestor (TMRCA) of all wolves and dogs but excluding the coyote along the genome. We reported TMRCAs averaged over bins of 1,000 bases and found that the region of interest on chromosome 25 had the youngest TMRCA genome-wide (**Fig. 3d**).

Inferring a selection coefficient for the *IFT88* sweep using CLUES
We used CLUES[39] to infer a selection coefficient for the sweep at the *IFT88* locus on chromosome 25. We aim to find the most likely selection coefficient producing the observed genetic variation in this region. However, this requires integration over genealogical histories, which is intractable. CLUES instead utilises genealogical histories inferred by Relate to compute the likelihood of each selection coefficient. This method utilises an importance sampling scheme to correct for the fact that the Relate-inferred histories will be biased if the focal variant is under selection, as the Relate prior assumes neutrality of all variation.

We used the Relate-inferred genealogies from the previous TMRCA calculation. The lead SNP from the ancient genome selection scan at chr25:17440320 was filtered out in the mappability mask, and we therefore first mapped a mutation fixed in wolves and dogs but not carried by the coyote to the local genealogical tree at chr25:17440320 using the function RelateExtract --mode MapMutations which is provided with the Relate package. We then extracted the genealogical tree at this position using the function RelateExtract --mode AncMutForSubregion. Relate can sample different branch lengths while keeping tree topology fixed to obtain an ensemble of possible histories at this locus. We obtained 100 samples using RelateCoalescentRate --mode SampleBranchLengths with option --format b to output in the format used in CLUES. Finally, we run CLUES (obtained from https://github.com/35ajstern/clues/) and set the time cutoff parameter tCutoff to 33,333 generations (100k years). This implies that we assume a selection coefficient of zero at times older than this cutoff. We obtained a maximum likelihood estimate for the selection coefficient of 0.01982 with a log-likelihood ratio of 10.15.

Genotyping the K^B melanism deletion

A three base pair deletion in the gene CBD103 has been shown to dominantly confer black coat colour in wolves and dogs[40]. It is relatively common in dogs, in North American wolves and in some populations of Italian wolves, but otherwise rare among wolves[41]. It has been detected using targeted PCR amplification in ancient individuals with dog morphology as early as in the early Holocene, and in one ancient individual with more robust, possibly wolf or more likely dog-wolf hybrid, morphology (the Ulug Depe site, Turkmenistan, ca. 5550 - ca. 4000 cal. BP)[42]. In North American wolves there is reduced diversity around this locus, suggesting a recent selective sweep, and it has been proposed that the allele introgressed from dogs in the Holocene[41,43].

We looked for this deletion in all the ancient wolf genomes by extracting the read alignments at the locus using samtools tview (*samtools tview --reference $canFam3 -p chr16:58965408 -d T*) and examining these visually. Only one wolf genome, the 14k-year old Tumat2 individual[5], showed the presence of the deletion. It has three reads containing the deletion and three reads not containing the deletion, suggesting a heterozygous genotype (**Fig. S19**).

The observation in a late Pleistocene Siberian wolf represents the earliest observation of the deletion so far. This finding does not necessarily change the understanding of the history of this deletion in North American wolves, where it still can have introgressed from dogs and then been the target of natural selection[41,43]. But the finding increases the probability that the origin of the deletion was in wolves, and that it was already segregating at low frequency in late Pleistocene wolves at the time of dog domestication. At 14 kya, the Tumat2 wolf most likely lived after the emergence of the domestic dog population, meaning that an early origin of the deletion in a domestic population followed by gene flow into Tumat2 cannot be ruled out. It also cannot be ruled out that the deletion could have arisen independently more than once.

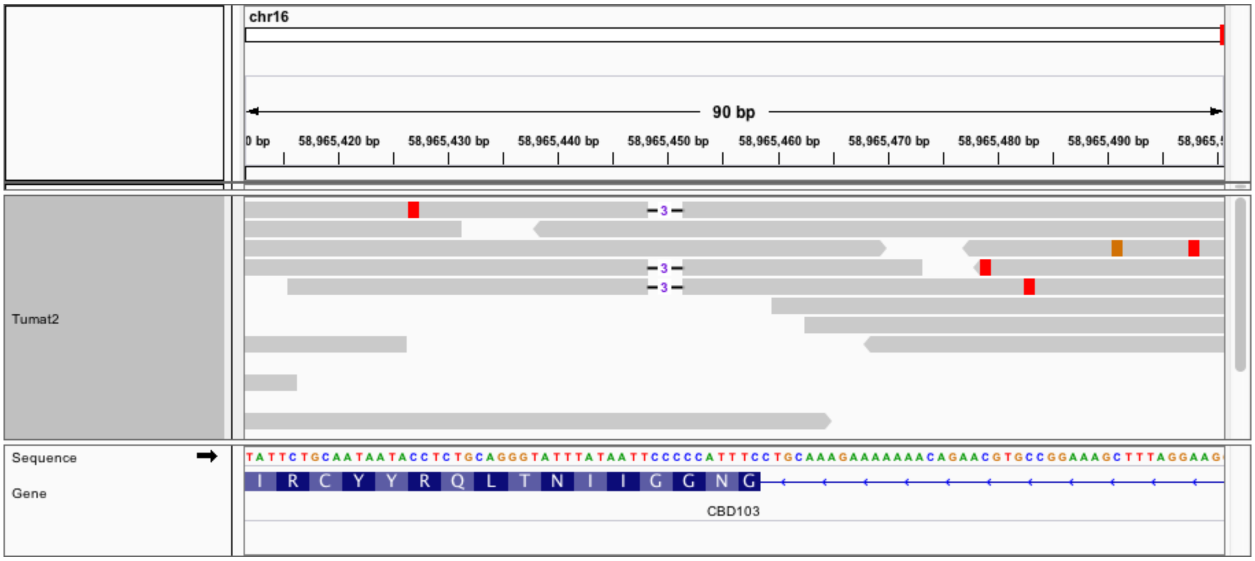

Fig. S19. The K^B deletion observed in the 14k-year old Tumat2 wolf. A screenshot from the IGV browser[44] showing reads mapped to the canFam3.1 reference genome for Tumat2, including three reads that carry the three base pair K^B deletion. All of the reads that span the site of the deletion have mapping qualities of 37. Red bases correspond to T bases, and two instances of C → T likely caused by ancient DNA deamination damage occur on reads that contain the deletion.

SI Section 5: Dog ancestry analyses

Model-free analyses of dog relationships to wolves

We performed several model-free analyses aiming to place the ancestry of dogs within the context of modern and ancient wolf diversity. First, we performed PCA analyses based only on the specific subset of f_4-statistics that have the following form: $f_4(X,A;B,C)$, where A, B, and C are wolves that lived prior to 28 kya, i.e. before the last glacial maximum and most likely before the emergence of dogs. For each target individual X, the input is thus a vector of f_4-statistics that are informative of its relationships to pre-LGM wolf genomes.

For the pre-LGM set, we excluded ancient wolves with strong reference bias, as there is a concern that such individuals could display spurious attractions to any target individuals that also display reference bias. We quantified reference bias for each individual X using the f_4-statistic f_4*(CoyoteCalifornia,X;Boxer,Dog reference)*, which takes advantage of the fact that the dog reference genome is derived from a Boxer individual, meaning that any attraction towards the reference genome over another Boxer genome should reflect reference bias. Wolves with f_4 values larger than 0.00025 were excluded, leaving 21 wolf genomes: *Germany_Aufhausener.AH574, Germany_Aufhausener.AH577, Siberia_Yana.CGG27, Siberia_Badyarikha.CGG34, Alaska_Fairbanks.JAL385, Alaska_Fairbanks.JAL48, Alaska_Fairbanks.JAL65, Alaska_Fairbanks.JAL69, Yukon_HunkerCreek.SC19.MCJ017, Germany_HohleFels.JK2174, Germany_HohleFels.JK2175, Germany_HohleFels.JK2183, Siberia_BungeToll.LOW003, Siberia_UlakhanSular.LOW008, Czechia_Predmosti.PDM100, Alaska_LillianCreek.ALAS_024, Siberia_Tirekhtyakh.VAL_033, Siberia_Badyarikha.VAL_008, Siberia_Ogorokha.VAL_050, Siberia_BelayaGora.IN18_016, Siberia_Tirekhtyakh.CGG32*

The target set of individuals to be analysed were then post-LGM and present-day wolves and dogs. The matrix of f_4-statistics for the target individuals was transformed into a distance matrix using $\sqrt{2 \times (1 - r)}$ where r is the Pearson correlation coefficient for a given pair of individuals. PCA was then performed on the distance matrix using the ppca function from the pcaMethods R package. Seven target wolves, all of which had low sequencing coverage, displayed erratic behaviour in these PCA analyses, pulling out their own PC, and were therefore excluded: *Ireland_Ballynamintra.367, Ireland_Plunkett.IRK, Siberia_Yana.CGG19, Siberia_NikitaLake.CGG20, Siberia_Berelekh.CGG21, France_Noyen.CH1109, Russia_Shaitanskaya.AL3284*. Dogs were kept out of the calculation of the PCs, and were then individually projected onto them by re-running the PCA once for each given dog, with that single dog added in and saving its coordinates (the correlation between these replicate PCs and the original PCs was >0.998 in all cases for PCs 1 to 4, meaning stochasticity between these runs was minimal). Eight dogs, all of which had low sequencing coverage, displayed erratic behaviour in these PCA analyses, and were therefore excluded: *Israel_7200BP.THRZ02, Russia_Baikal_7000BP.C26, Russia_Baikal_7400BP.C27, Sweden_StoraForvar_4000BP.C94, AL2946_Plocnik.AL2946, OL4029_Spain.OL4029, AL3223_Weyanoke.AL3223, Sweden_PWC_C90.C90*.

The results reveal the major features of post-LGM wolf population structure (**Fig. 4a, Extended Data Fig. 2a,b**), with PC1 representing deep (e.g. non-gray wolf) ancestry and separating out both North American wolves (due to their coyote admixture), and Tibetan wolves (due to their unknown, deep ancestry). PC2 largely separates wolves by an east-west axis, from North America, late Pleistocene Siberia, present-day China, late Pleistocene and present-day Europe, and lastly to the present-day Near East.

Dogs (including both modern and ancient individuals) fall towards the eastern side of this wolf structure, largely clustering with late Pleistocene Siberian wolves from 23-13 kya. However, a cline is also visible within the dogs. Colouring dogs on the basis of the f_4-statistic f_4*(AndeanFox,X;Tel Hreiz dog 7k BP,Zhokhov dog 9.5k BP)*, which captures a major axis of dog population structure between Eastern Eurasian and Near Eastern dogs[45], reveals that the cline in the relationship to wolves correlates with this aspect of dog population structure. More Eastern-related dogs fall more in the easterly direction in the PCA (towards Siberian and North American wolves), while more Near Eastern-related dogs fall more in the western direction (towards European and Near Eastern wolves).

Excluding populations with more divergent ancestry can allow for principal components that reveal more subtle structure among the remaining populations. We therefore repeated this analysis after excluding two small sets of present-day wolves which tended to generate their own clusters: Tibetan wolves (*Wolf42Tibet*, *WolfTibetan01InnerMongolia*, *WolfTibetan02InnerMongolia*, *WolfTibetan03QinghaiHighland*, *WolfTibetan04QinghaiHighland*) and two North American wolves with higher proportions of coyote ancestry (*Wolf18GreatLakes*, *Wolf40IsleRoyale*). These results were similar, but with some differences. For example, dogs do not cluster with the Siberian wolves from 23-13 kya in PC1-PC2 space. The same cline within dogs is visible in these results (**Extended Data Fig. 2c,d**).

In a traditional PCA analysis based on genotypes and present-day genomes, it would be difficult to distinguish between a dog cline resulting from dog admixture into local wolves, and a dog cline resulting from a scenario in which more than one wolf progenitor independently contributed to dog ancestry. However, in this “pre-LGM f_4-PCA”, dog admixture into local wolves should not by itself give rise to a cline within dogs, as it would not impact the pre-LGM $f_4(X,A;B,C)$ values of dogs that are computed using wolves that likely lived before the time of dog domestication. It would shift the affected wolves towards the dog cluster, because those wolves would become more similar to dogs in their profiles of pre-LGM $f_4(X,A;B,C)$ values, but it would not shift dogs away from each other. Therefore, the cline within dogs should reflect either different amounts of local wolf admixture into different dogs, or that dogs originated from more than one wolf progenitor population — these two possibilities are essentially equivalent from an f_4-statistics point of view.

We also performed this analysis using only dogs as a target set. That is, we clustered dogs using PCA on their profiles of pre-LGM $f_4(X,A;B,C)$ values, without including post-LGM and present-day wolves in projection or any other part of the analysis. PC1 in this analysis correlates

strongly with sequencing coverage, and is thus a technical aspect of the data. However, PC2 recapitulates the same cline within dogs observed above, separating Eastern Eurasian from Near Eastern dogs (**Fig. S20**). This result could not be affected by dog admixture into post-LGM wolves, as those wolves are not involved in the analysis in any way. Instead, it must reflect differences among dogs in their profiles of pre-LGM $f_4(X,A;B,C)$ values, that is, differences in their relationships to wolves that lived before the last glacial maximum, and so likely prior to the emergence of dogs.

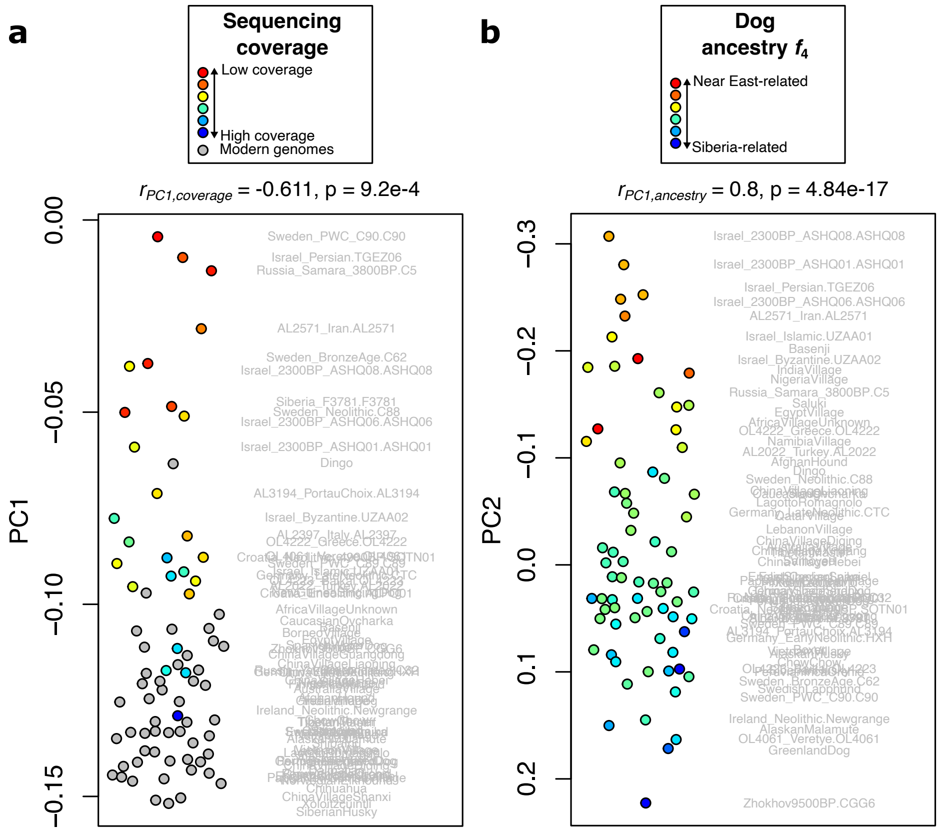

Fig. S20. Dog population structure recovered from differential relationships to pre-LGM wolves. PCA only on dogs X, based on f_4-statistics only of the form $f_4(X,A;B,C)$, where A, B, and C are wolves that lived prior to 28 kya. **a**) PC1 correlates strongly with sequencing coverage, thereby representing a technical aspect of the data rather than ancestry. Ancient dogs are coloured according to sequencing coverage, and modern dog populations (which often contain several individuals of varying coverage) are grey. **b**) PC2 correlates strongly with known dog population structure, largely separating Eastern Eurasian dogs from Near Eastern and African dogs. Dogs are coloured according to the f_4-statistic f_4*(AndeanFox,X;Zhokhov dog 9.5kya,Tel Hreiz dog 7kya)* (the Zhokhov dog itself has no value for this statistic, but is given the darkest blue colour). The recovery of PC2 in the absence of any wolves that lived more recently than 28 kya demonstrates that this axis of dog population structure must reflect differences in the relationships to wolves that lived before this time. The *r* values indicated above each plot are Pearson correlation coefficients.

f_4-statistics relating dogs to ancient wolves

We directly studied particular f_4-statistics that relate dogs to ancient wolves. We find that dogs overall are more similar to an 18k-year old wolf from Siberian than to a 13k-year old wolf from Germany (**Fig. 4b**, **Extended Data Fig. 3**). This mirrors the PCA results, in which dogs cluster more towards Siberian rather than west Eurasian wolves (**Extended Data Fig. 2**). Similarly, dogs overall are closer to the 18k-year old Siberian wolf than to three other wolves: an 18k-year old wolf from Eliseevichi in western Russia, a 5k-year old wolf from Sweden, and a 5k-year old wolf from Kazakhstan. Dogs display no significant differences in their relative similarity to the 18k-year old Siberian wolf and a 21k-year old wolf from Shaitanskaya Cave in the Ural mountains, however the latter has very low sequencing coverage (0.05x) and strong reference bias, prohibiting any strong conclusions. Dogs are slightly more similar to the 18k-year old Siberian wolf than to a 15k-year old wolf from Alaska, which appears to lack coyote admixture (**Fig. 2b**) and may have been a recent migrant from eastern Siberia.

The magnitude of these f_4-statistics and how they compare to those of wolves are also informative about the relative position of dogs within the overall diversity of wolves. For example, in the contrast between the 18k-year old Siberian wolf and the 13k-year old German wolf, dogs display f_4 values that are approximately as Siberian-shifted as those of North American wolves, and more Siberian-shifted than those of present-day Chinese and other East Eurasian wolves. They are, however, slightly less Siberian-shifted than Late Pleistocene Siberian wolves (**Extended Data Fig. 3**).

These simple comparisons show that dog ancestry overall is relatively more similar to post-LGM Siberian than to post-LGM west Eurasian wolves, and that the post-LGM Siberian wolves constitute the ancient wolves within our dataset that are most similar to dogs. However, these comparisons are only relative, and do not inform on whether any of these sampled ancient wolves are closely related to dogs in an absolute sense.

When only plotted against time, any variation among dogs in the magnitude of their f_4-statistics is not necessarily readily visible. However, when plotting pairs of f_4-statistics against each other, it is clear that dogs of different ancestry show different relative affinity to ancient Siberian and European wolves (**Fig. 4b**). Near Eastern and African dogs are relatively more European wolf-shifted than Eastern Eurasian and American dogs, with Neolithic and later European dogs intermediate, mirroring the cline revealed in the PCA analyses (**Extended Data Fig. 2**, **Fig. S20b**).

qpWave tests of dog cladality

We used the *qpWave* method[46] to further test whether dog ancestry is consistent with having arisen from a single stream of ancient wolf diversity. *qpWave* uses a target set of populations (or “left” populations within the *qpWave* framework) and a reference set of populations (or “right” populations), and analyses all f_4-statistics of the form f_4*(target,target;reference,reference)*. If all the target populations have a fully shared history since their divergence from (or last episode of

gene flow from) all of the reference populations, we say that the targets form a clade and derive from a single stream of ancestry relative to the reference set. In such a scenario, all f_4-statistics of the above form are expected to be 0, as all allele frequency differences between any two targets should only reflect genetic drift that has occurred since the formation of the clade, and these differences therefore will be uncorrelated to the allele frequency differences that exist between any two reference populations. In other words, all of the targets will be symmetrically related to the reference populations, and none of the reference populations will display excess affinity (i.e. non-zero f_4-statistics) to one target population over another. *qpWave* assigns a p-value to the hypothesis that the target populations form a clade. *qpWave* also tests whether the data is consistent with a greater number of streams than one, but given the low data quality associated with ancient DNA, we are not certain if those results are necessarily robust for our data, and we do not draw conclusions from those results.

We defined two different target sets of dogs: an "Eastern dogs" set containing five dogs that fall towards the eastern end of the cline in the exploratory f_4 and PCA analyses, and one "Southwestern" (as in southwestern Eurasia) set containing four dogs that fall towards the western end of the cline. We also defined two different reference sets of ancient wolves that are older than 28 kya (i.e. pre-LGM): one "small" set with seven wolves, and one "large" set with 25 wolves. A concern with using only a large set is that this leads to more opportunities for asymmetries (non-zero f_4-statistics) between the target and reference set due to technical biases alone (e.g. reference bias or batch effects). This is why we defined two different reference sets. We then tested the cladality of these dog target sets, as well as their union ("East + Southwest"), in turn using *qpWave* (**Extended Data Table 2**).

qpWave cannot reject the conclusion that the eastern dog set is a clade, neither with the large (p = 0.0656), nor the small (p = 0.3667) wolf reference sets. Notably, the eastern set includes the 10.9k-year old Veretye dog from Karelia in northeastern Europe, which therefore suggests that there is no evidence for any western wolf-related ancestry in this individual. Thus, the ancestry of these eastern dogs is consistent with a scenario in which they were all derived from a single wolf population. However, if eastern dogs derived ancestry from multiple wolf populations, each of which displayed the same relationships to the wolves included in the reference sets, that would not be detected here. Such a scenario in which eastern dogs were derived from multiple wolf populations of similar ancestry thus cannot be ruled out. The "single stream of ancestry" conclusion from *qpWave* is thus not fully equivalent to "a single population".

qpWave cannot strongly reject the conclusion that the southwestern dog set is a clade with the large reference set (p = 0.1622), but it can with the small reference set, though the p-value is not strong (p = 0.0229). The dogs included in this set likely have varying amounts of eastern- and western-related ancestries (**Fig. 4d**), such that we should expect that *qpWave* could detect those differences and therefore reject the clade. However, in practice, the power to do this might not be sufficiently large given the relatively small number of dogs included in this set.

When taking the union of the two dog target sets ("Eastern + Southwestern"), *qpWave* can strongly reject that all of these dogs together form a clade, with both the large ($p = 9.2 \cdot 10^{-18}$) and

the small ($p = 6.1 \cdot 10^{-5}$) reference sets. This result thus formally confirms the results implied by the cline that dogs display in the PCA analyses (**Fig. 4a**, **Extended Data Fig. 2**, **Fig. S20**), that more than one wolf population has contributed to dog ancestry. Importantly, this *qpWave* analysis reaches this same conclusion with a reference set including only wolves that lived before 28 kya, and thus likely before dog domestication, which means that the result should not be affected by later admixture from dogs into wolves.

We also performed analogous *qpWave* tests using present-day wolves as reference sets instead of ancient wolves. If there has been widespread dog admixture into present-day wolves, we would expect that the *qpWave* analysis would reject any dog clade due to the asymmetries between different dogs and wolves resulting from that admixture. To enable an analysis that can be fairly compared to the above analyses using ancient wolf reference sets, we constructed present-day wolf reference sets of the same size (7 or 25 individuals). To circumvent stochastic sampling effects and variation in ancestry and dog admixture between different wolves, we constructed 100 replicate reference sets through random sampling from a base set of 35 present-day Eurasian wolves, and ran the analyses separately for each set. The results are summarised by taking the mean (on a log-scale) and the maximum *qpWave* single stream p-values from across the 100 tests (**Extended Data Table 2**).

Focussing on the eastern dog set—the cladality of which could not be rejected relative to ancient wolves that lived prior to 28 kya—we find that its cladality is strongly rejected when using present-day wolves as reference sets, both with large (mean p-value across 100 replicates = $2.2 \cdot 10^{-46}$) and small (mean p-value across 100 replicates = $2.5 \cdot 10^{-11}$) reference sets. This thus demonstrates that, when viewed from the point of view of present-day wolf diversity, the clade status, and thus the inference of a single origin, of eastern dogs (relative to the ancient wolf diversity) is obscured. This likely reflects differential dog admixture, in different amounts and from different dog sources, into present-day wolves.

The finding that the eastern dog clade is not rejected when using ancient wolf reference sets, but is rejected when using modern wolf reference sets, demonstrates the value of ancient wolf genomes for understanding dog origins. The ancient wolf genomes circumvent the more recent admixture complexity in present-day wolves, and allow the clade status of eastern dogs to be found. If only present-day wolves were available for these analyses, it would have been difficult or impossible to reach this conclusion.

qpAdm modelling of eastern dog ancestry

We applied the same rotating *qpAdm* model testing framework with ancient wolf sources described in the section "*qpAdm modelling of post-LGM wolf ancestry"* to dog targets. We found that single-source models can be strongly rejected for all analysed dogs, including the models that have the 18k-year old *Siberia_BelayaGora.IN18_005* as the source (**Fig. 4c**). While this individual and other similar post-LGM Siberian wolves represent the ancient wolves in our dataset that dogs show the strongest similarity to (**Fig. 4a**), they are thus unlikely to be the immediate ancestors of dogs, or to be closely related to the ancestors of dogs.

In order to fit dog targets, two-source models are instead required. Focussing on eastern dogs such as the 9.5k-year old Zhokhov individual and the present-day New Guinea Singing Dog, good fits are achieved by the model that has the 18k-year old *Siberia_BelayaGora.IN18_005* as the first source and the outgroup population (an ancient dhole) as the second source. The estimated ancestry proportions in these models for Eastern dogs are 80-90% Siberian-related and 10-20% outgroup component (i.e. Zhokhov: 11.7% outgroup component, standard error 3.4%; New Guinea Singing Dog: 15% outgroup component, standard error 3.4%).

Local differentiation reflected in unsampled ancestry components in *qpAdm*

How can these *qpAdm* results on the ancestry of dogs be understood? We use an outgroup species as one of the sources in these analyses (an ancient dhole genome, although results are similar when using a coyote or Andean fox), but we do not think that dogs actually have ancestry from this other species. Dogs do not show evidence for non-gray wolf ancestry in other analyses, in contrast to, for example, North American wolves and possibly Tibetan wolves. Instead, we believe the best fit of 10-20% of dog ancestry to the outgroup population more likely reflects the model's attempt to account for the true source being an unknown wolf ancestry that is not represented by the ancient genomes in our set of candidate sources. More precisely, some ancestry that is to some extent divergent from, and lacks shared genetic drift with, the available ancestries. Thus, the fact that the best fitting *qpAdm* models involve two sources does not necessarily imply that eastern dogs such as the Zhokhov dog emerged from two distinct source populations—instead, it could reflect *qpAdm* approximating the ancestry of a single progenitor population with the sources that are available to it.

In the f_4-statistics that *qpAdm* relies on, such ancestry will give rise to negative values for statistics of the form f_4*(Outgroup,Early Siberian wolf;Later Siberian Wolf,Dog)*, meaning there is some attraction among Siberian wolves over time to the exclusion of dogs, which thus experience outgroup attraction. A direct evaluation of statistics of this form confirms negative values for dogs (**Fig. S12**). This is the case even for f_4*(Armenia_Hovk1.HOV4,Siberia_Enygen.VAL_18A;Siberia_BelayaGora.IN18_005,Dog)* featuring the ~100k-year old wolf *Siberia_Enygen.VAL_18A*, implying that the non-Siberian-related ancestry in dogs diverged from Siberian wolves before ~100 kya. There is thus a small degree of temporally deep structure between dogs and Siberian wolves, and this is likely what the *qpAdm* results are reflecting when a minority fraction of outgroup ancestry is assigned.

Many other wolves also have ancestry that is divergent from Siberian wolf ancestry, meaning that dogs are not unusual in this respect. Indeed, dogs display values of f_4*(Outgroup,Early Siberian wolf;Later Siberian Wolf;Dog)* that are similar in magnitude to those displayed by European and Chinese wolves (and smaller than those of Tibetan and Near Eastern wolves) (**Fig. S12**), suggesting that the degree of structure between Siberian wolves and dogs might be similar to the degree of structure between Siberian wolves and each of European and Chinese wolves.

In the case of European wolves, we now have a good understanding of their history and relationships to Siberian wolves throughout the late Pleistocene, because we have ancient European wolf genomes spanning the last ~60 kya. Our results (e.g. **Fig. 2a**) show that European wolves have been highly genetically connected to Siberian wolves over time, but have nonetheless maintained a small degree of differentiation that is manifested in these analyses as a minority component of deep ancestry that is divergent from Siberian wolf ancestry. It is possible that the ancestors of dogs would have had a similar relationship to Siberian wolves, wherein most of their ancestry was continually homogenised throughout the Late Pleistocene, while retaining a minority fraction of deep, local ancestry.

To further test this hypothesis, we performed *qpAdm* experiments in which we kept out the pre-LGM European wolf genomes in order to test how post-LGM and present-day European wolves would be modelled if the only available sources were Siberian (these experiments are described further in SI section 3, under "Persistence of deep local ancestries"). We found that in this set-up, European wolves obtain results that are very similar to those of dogs: they are modelled as 80-90% Siberian-related and 10-20% unsampled, divergent ancestry (**Fig. S11**). This demonstrates that the phenomenon of non-Siberian, unsampled ancestry in *qpAdm* is not unique to dogs, that it can reflect deep local structure between otherwise highly connected wolf populations, and that whether this behaviour is manifested or not depends on what ancient wolf genomes are available as sources. It thus seems likely that Eastern dog ancestry will have emerged from a population that had a relationship to Siberian wolves that was similar to the relationship that European wolves have to Siberian wolves - but which lived in some other, as yet determined part of the world. The *qpAdm* results obtained for dogs, whose ancestry is modelled as mostly Siberian-related and partly unsampled, can then likely be viewed as approximating this relationship involving high interconnectivity but also some degree of differentiation (**Extended Data Fig. 4**).

Present-day Chinese wolves are the best fit for dog ancestors currently available, but recent gene flow is an unknown factor

We tested whether any other post-LGM wolves - including present-day wolves - might serve as better proxies for dog ancestry than those included in the main *qpAdm* analysis described above. For each tested wolf X, we repeated the rotating *qpAdm* tests using the Siberian Zhokhov dog (9.5k BP) as the target and with X added in as one of the candidate sources (and like the other candidate sources, when X is not used as a source it is part of the reference set). All possible models with up to two sources were tested. A number of observations can be made from the results (**Extended Data Fig. 6**):

- For most wolves X, the Siberia (*Siberia_BelayaGora.IN18_005*, 18 kya) + unsampled ancestry model that was favoured in the main *qpAdm* analysis remained the best-fitting model, even with X added to the reference set. This is especially the case for both West Eurasian and North American wolves.

- For a few West Eurasian wolves X, the Siberia + unsampled ancestry model was strongly rejected, but so was the single-source X model (e.g. *Wolf07Israel*, *Wolf27Spain*). X is thus not a good proxy for dog ancestry, but the Siberia + unsampled ancestry model fits much worse when X is present in the reference set. A likely explanation for this is dog admixture in X, which would introduce an affinity between X and the dog target and therefore break the Siberia + unsampled ancestry model.

- For a few ancient wolves X with low sequencing coverage, many different models fit well: the Siberia + unsampled ancestry model, the single-source X model, and many two-source models involving X plus some other ancient wolf. This most likely reflects the low power to reject models when the sequencing coverage is low, and these wolves are indicated with yellow backgrounds in the figure. Few conclusions can therefore be drawn from these genomes, though for at least two of them, *Russia_Shaitanskaya.AL3284* and *Ireland_Ballynamintra.367*, the single-source X model could still be rejected, suggesting they are not matches for eastern dog ancestry.

- The set of ancient wolves from the period 25-10 kya which can be rejected as matches for Eastern dog progenitor ancestry (indicated with crosses in **Fig. 4e**), are: *France_Auneau.TU839*, *Ireland_Plunkett.IRK*, *Germany_HohleFels.JK2179*, *Ireland_Ballynamintra.367*, *Siberia_Tumat.Tumat1*, *Siberia_Tumat.Tumat2*, *Germany_HohleFels.JK2181*, *Alaska_NorthSlope.ALAS_016*, *Siberia_UlakhanSular.CGG33*, *Russia_Eliseevichi.AL2657*, *Siberia_BelayaGora.IN18_005*, *Russia_Shaitanskaya.AL3284*, *Siberia_Uyandina.VAL_011*

- For a wolf X to be a better proxy for Eastern dog ancestry than the 18 ky-old Siberian wolf, two things should be observed in these results: that the Siberia + unsampled ancestry model is strongly rejected when X is included in the reference set, and that the single-source X model achieves a good fit. Both of these are observed for a few present-day wolves from China: *Wolf38Shanxi*, *Wolf34Shanxi* and *Wolf31Liaoning*, with similar results though slightly less well-fitting single-source models for *Wolf05China*, *Wolf37InnerMongolia* and *Wolf04InnerMongolia* (whereas wolves from western China, i.e. Xinjiang and Tibet, perform less well).

  These results suggest that dog ancestry might be closer to these present-day East Asian wolves than to the 18 ky-year old Siberian wolf. The fit of the single-source models, without the need for an unsampled ancestry component, also implies that the ‘missing’ ancestry in dogs which is not represented among our ancient wolf genomes, is no longer

missing when these present-day East Asian wolves are used as sources. However, very strong conclusions about dog origins cannot be drawn from the results on these genomes for two main reasons. Firstly, since these are present-day genomes, they could have dog admixture which would influence the results (although a minor proportion of dog admixture in itself should not cause a ‘false positive’ fit for a single-source model if the rest of the genome is a poor proxy - see for example the results for *Wolf07Israel* and *Wolf27Spain* discussed above). Secondly, we do not know where the ancestors of these wolves lived at the time of dog domestication, meaning that their current geographical location will not necessarily correspond to the centre of origin of dogs.

qpAdm modelling of western dog ancestry

For western dogs, i.e. those from western Eurasia and Africa, the *qpAdm* results are different from those of eastern dogs. Unlike for eastern dogs, the two-source Siberia + unsampled ancestry model could be rejected for most European dogs, and very strongly rejected for ancient Near Eastern and present-day African dogs (**Fig. 4c**). This is consistent with the exploratory f_4 and PCA analyses, as well as the *qpWave* results, which suggest the presence of an additional component of wolf ancestry in these dogs.

We therefore analysed three-source models for Western dog targets, and found that the best ones feature, in addition to the 18k-year old *Siberia_BelayaGora.IN18_005* wolf and the outgroup component, a third source related to the 13k-year old European wolf *Germany_HohleFels.JK2179*. For several Western dogs, this three-source model achieved a good fit. For others, the p-values are below 0.01 but substantially higher than those of the two-source model (**Fig. 4c**). This suggests that the additional component of ancestry present in Western dogs is related to post-LGM European wolves. The 7.2k-year old Levantine dog THRZ02 is the one dog in our dataset that displays the strongest western affinity. Due to its low coverage (0.1x), there is not enough statistical power to distinguish between different *qpAdm* models for its ancestry, but assuming the three-source model applies to this dog, the estimates for the amounts of European wolf-related ancestry in it should be reasonable. The estimates for THRZ02 are 62% Siberian-reated, 6% unsampled ancestry and 32% European-related.

Is the western-related wolf ancestry in western dogs derived from the European wolf populations that we have sampled, or from an unsampled population that is related to them? In principle, we could address this question in the same way that we addressed whether the sampled Siberian wolves were good proxies for the eastern-related dog ancestry: can a given wolf as the sole source achieve a good *qpAdm* fit for dog ancestry, or is an outgroup component representing some degree of unsampled, divergent ancestry also required? However, this approach runs into a limitation when modelling western dog targets, because the models already require an unsampled ancestry component to fit the eastern-related part of their ancestry. If the western-related component also requires a different unsampled ancestry, those two unsampled ancestries would collapse and be modelled with a single component. It would

not be possible to reliably disentangle the two different unsampled ancestries, which would lead to a poorly specified model unlikely to produce accurate ancestry proportions.

To overcome this limitation we took an alternative approach, using the 9.5k-year old Zhokhov dog from Siberia as a source in *qpAdm*. If we make the assumption that this dog is a good representation of the eastern dog ancestry, it can thus serve as a baseline for that ancestry, screening out the more distal, unsampled wolf source and its representation in the model as Siberia + unsampled ancestry. For these analyses, we also extended the *qpAdm* reference set with five additional pre-LGM genomes to potentially provide greater constraints on ancestry proportions (age estimate in parentheses, years BP): *Siberia_Badyarikha.VAL_008* (33515), *Germany_HohleFels.JK2175* (33163), *Siberia_Yana.CGG23* (33020), *Siberia_Letniaya.LOW002* (32781), *Siberia_Ogorokha.VAL_005* (32419). For these analyses we did not perform a full rotating approach, rather we only rotated the Zhokhov dog, the European wolf *Germany_HohleFels.JK2179* and the outgroup dhole *Armenia_Hovk1.HOV4*.

We found that *qpAdm* cannot fit western dogs as a combination of the Zhokhov dog and the 13k-year old European wolf. Instead, the best-fitting models require a third, unsampled ancestry contribution (**Fig. S21**). This suggests that the European wolves that we have sampled here, while related to the source of the second component of ancestry in western dogs, are in fact not a match for the source population. Instead, there is some degree of structure between the sampled European wolves and the true source. This is very much analogous to how there is some degree of structure between the sampled Siberian wolves and the true source of the eastern dog component, as discussed above. Looking at the models that feature both the ancient European wolf and the unsampled ancestry component, the proportions of European-related ancestry is maximised in ancient Near Eastern and present-day African dogs (i.e. 55.4% in the 7.2k-year old THRZ02 from Israel). For European dogs the results are more variable: for some targets the single-source Zhokhov model is not rejected, for others it is rejected but models including the ancient European wolf do not necessarily do much better, and sometimes the estimated proportion of ancestry assigned to the European wolf is below 0. This likely reflects overall low amounts of the European-related ancestry in European dogs, resulting in less power to detect and accurately quantify it.

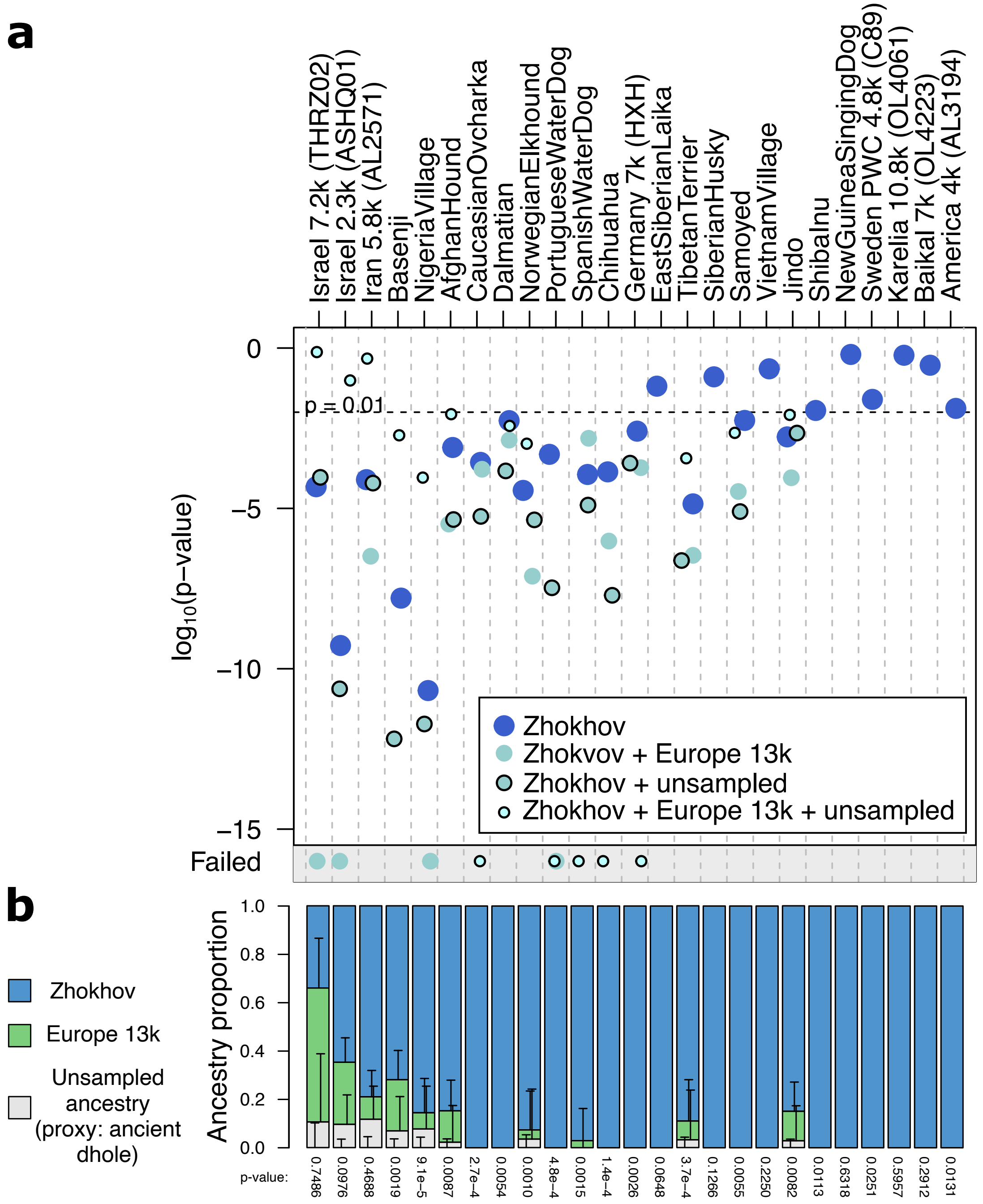

Fig. S21. Testing whether European wolf ancestry can explain the second component of ancestry in western dogs. a) The 9.5k-year old Zhokhov dog is used here as a baseline to represent the eastern dog component. For every dog target, the following models are tested with *qpWave*/*qpAdm*: a single-source Zhokhov model, a two-source Zhokhov + European 13k wolf model, a two-source Zhokhov + unsampled ancestry model, and a three-source Zhokhov + European 13k wolf + unsampled ancestry model. Models placed within the gray space labelled “Failed” either had p-values that fall below the lower limit of the plot, or had ancestry proportions that were higher than 1.1 or lower than -0.1. If a target has a model with p > 0.01, models with a larger number of sources are not plotted, to avoid clutter. b) Estimated ancestry proportions for the best-fitting model for each target. Bars denote ±1 standard error estimated from a block jackknife.

Given that ancient European wolves do not appear to be a match for the western-related wolf ancestry in western dogs, we tested if any other available wolf genomes - ancient or present-day - could be better candidates. To do this, we took all available post-LGM and present-day wolf genomes, and for each of them in turn repeated the *qpAdm* analysis described above, but with the given wolf added in as a rotating source. Using the African Basenji dog as a target (which is more suitable than the 7.2k-year old Levantine dog THRZ02, as the relatively lower coverage (0.1x) of the latter gives *qpAdm* less power to distinguish between models), we were able to evaluate whether any of these wolves, when combined with the Zhokhov Siberian dog, could explain the missing ancestry in western dogs.

The results show that none of the ancient or present-day European wolves provide good fits to western dog ancestry. As above, models involving European wolves also require an unsampled ancestry component to achieve a good fit (**Extended Data Fig. 7**). Similarly, models involving Chinese, central Asian (i.e. Kazakhstan, Altai mountains, Shaitanskaya in the Ural mountains) and North American wolves can also be rejected unless including a third, unsampled ancestry component. However, there are five wolves which provide good fits ($p > 0.01$) to the second component of ancestry in the African Basenji dog: present-day wolves from Syria, Israel, Iran, India and (although with a slightly worse fit) Saudi Arabia. Two-source models involving the Zhokhov dog and one of these present-day wolves provide good fits without the need for an unsampled ancestry component, implying that there is no “missing” ancestry left to explain in these models.

A source of the western wolf-related component of dog ancestry somewhere in the Near East or surrounding regions would align well with the observation that this type of ancestry is maximised in ancient Near Eastern and present-day African dogs (the latter which have a strong relationship to, and likely derive from, Near Eastern dogs[45]), with lower levels in European and central Asian dogs. The Indian wolf analysed here also provides a good fit, but this individual (inw, Wolf19 in the 722g dataset, BioSample accession SAMN02921311) is from a zoo and likely has recent admixture from Iranian wolves, meaning it might not necessarily be representative of Indian wolves more broadly.

It is possible that there is dog admixture in these present-day Near Eastern wolves, and if so it would likely be from local dogs that would be similar to the Near Eastern and African dogs modelled here. If this is the case, it would lead to an overestimation of the Near Eastern-related contribution to the modelled dogs. However, the crucial factor in terms of rejecting these Near Eastern wolves as sources is whether they carry some other, divergent ancestry that lacks shared genetic drift with the true source population for western dogs. Dog admixture might dilute the amount of such a divergent ancestry, but not get rid of it, such that *qpAdm* would still be expected to reject a poorly matching source even in the presence of dog admixture. Therefore, we do not believe that dog admixture would lead to a failure to reject the models involving these Near Eastern wolves as sources. Consistent with this, even though it is likely that some of the other wolves analysed here from other regions have dog admixture, they are all still rejected as sources.

Testing for a correlation between present-day dog and wolf population structures

Our findings from the ancient wolf genomes demonstrate that there are two different components of wolf ancestry in dogs: one eastern-related ancestry which is related to, but distinct from, Late Pleistocene Siberian wolves, and one western-related ancestry which is related to, but distinct from, Late Pleistocene European wolves and more closely related to present-day Near Eastern wolves. The eastern-related ancestry is present in all dogs, while the Near Eastern-related component is found in west Eurasian dogs, in its highest amounts in ancient Near Eastern and present-day African dogs (**Fig. 4d**). These results, combined with our findings that part of the present-day wolf population structure is quite old (i.e. likely older than 100 kya), and was not completely erased at the last glacial maximum, suggests that the dual ancestry signal in dogs might be visible even in present-day wolf population structure.

To test this, we used an independent dataset of array (CanineHD BeadChip) genotypes from a large number of present-day wolves and dogs[47], a dataset that also included genotype data from a number of previously published studies[48–52]. While this dataset contains fewer genetic variants (106,420 SNPs), it includes a greater number and diversity of present-day wolves than the available whole genome sequences. We also included the whole genome sequences in these analyses, subsetted to the variants covered in the array data. PCA analyses were performed using smartpca from the EIGENSOFT 7.2.1 package[53], using the “poplistname” parameter to define which individuals should be used to calculate the principal components as opposed to be projected, and the “lsqproject: YES” and “shrinkmode: YES” parameters.

First, to as much as possible avoid confounding effects due to recent dog admixture in wolves, we performed an initial PCA analysis to identify and exclude wolves with dog admixture. This was performed on dogs and wolves together, resulting in a PC1 that separated dogs and wolves. A straight line was manually fitted onto the plot, and all wolves that fell beyond this line in the direction of dogs were excluded from further analyses. While this simple analysis might not eliminate wolves that have relatively small amounts of dog admixture, it should have been able to eliminate those wolves with larger amounts. This filtering step reduced the dataset to 211 wolves for the next set of analyses.

We performed a PCA using only wolves (with default smartpca outlier removal settings). The first four principal components separate the available present-day wolves into four main clusters corresponding to major geographical regions: Europe, the Caucasus and the Near East, China and central Asia, and Siberia (**Extended Data Fig. 5**). We then projected dogs onto these principal components. In the PC1-PC2 space, dogs project to the side of the plot that contains Chinese, central Asian and Siberian wolves, rather than towards west Eurasian wolves. This appears broadly consistent with an overall eastern Eurasian wolf affinity for dogs. In the PC3-PC4 space that separates Chinese and central Asian from Siberian wolves along PC4, dogs project on the Chinese and central Asian side of the plot. This appears broadly consistent with an overall affinity towards eastern Eurasian wolves south of northeastern Siberia, rather than in northeastern Siberia. However, we caution that strong conclusions about the geographical

origins of dogs cannot be drawn from these analyses of present-day wolves, as some of these wolves could potentially have dog admixture, and as wolf population structure might have been reshaped since the emergence of dogs.

The cline within dogs that demonstrates an increasing western-related wolf affinity in western dogs is also visible in these analyses. In PC1-PC2 space, western dogs, especially those from Africa and the Near East, project closer to the Caucasus and Near East wolf cluster than what East Asian and Arctic dogs do. In PC3-PC4 space, these same dogs are similarly pulled towards the Caucasus and Near East wolf cluster, and not towards European wolves. These results support the findings from the *qpAdm* modelling, which suggested that Near Eastern wolves are better candidates than European wolves for the second component of ancestry in western dogs.

In isolation, it would not be possible to determine if the clinal behaviour of dogs in these PCA analyses reflected dual ancestry in dogs, or recent dog admixture into wolves. Recent dog admixture into wolves might amplify the signal to some extent, but as the patterns observed here align with the results obtained from the analyses of ancient wolf genomes, it is likely that these patterns at least in part reflect the same underlying dual ancestry signal in dogs.

Reinterpreting evidence for gene flow between dogs and wolves

Our finding that dogs carry two components of ancestry, derived from genetically distinct wolf populations, has implications for how evidence for gene flow between wolves and dogs should be interpreted. Previous studies also contain various results which likely reflect the same dual ancestry signal described here, and can be re-examined in the light of this. Several previous analyses have detected asymmetrical relationships between dogs and wolves: wolves being closer to some dogs than to others, and/or dogs being closer to some wolves than to others. Since these have primarily been based on present-day genomes, it has not been straightforward to determine the directionality of the admixture: whether different wolves carry ancestry derived from different dog populations (i.e. through dog admixture), or whether different dogs carry ancestry from different wolf populations (i.e. as a result of independent origins or wolf admixture). With the ancient genomes presented here, we were able to determine that at least some of these asymmetries very likely reflect the latter.

Freedman et al.[26] analysed three present-day wolf genomes and three present-day dog genomes, and found affinities between pairs of dogs and wolves from nearby geographical regions: a wolf from China was more similar to a Dingo than to the African Basenji and a European Boxer, and a wolf from Israel was more similar to a Basenji and a Boxer than to a Dingo. They modelled this pattern as a reflection of bi-directional gene flow both between wolves and dogs in the Near East (Israel wolf and Basenji) and between wolves and dogs in East Asia (Chinese wolf and Dingo). Skoglund et al.[3] similarly found that Boxers and Dingoes cannot be related to a Chinese wolf and a European wolf in a simple tree with independent dog and wolf branches, but that gene flow is necessary to explain the affinity between the wolves

and dogs in the west and between the wolves and dogs in the east. The study also found that dog-to-wolf and wolf-to-dog gene flow could both explain the data equally well.

The results presented by these studies likely reflect the same east-west, wolf-dog asymmetry described here, though it is also possible that recent local gene flow has amplified the asymmetry in present-day wolves. The findings presented here provide new insights on the directionality behind the asymmetries by showing that they, at least in part, must be the result of ancestry from west Eurasian-related wolves in Near Eastern, African and European dogs. As a consequence, the relative affinity between Chinese wolves and the Dingo does not necessarily require gene flow between these two populations. Instead, it could be an effect of Near Eastern wolf-related gene flow into the Basenji that has diluted the relative affinity of the latter to Chinese wolves.

Bergström et al.[45] similarly described widespread asymmetries between wolves and dogs, and also proposed that most of this could be explained by recent admixture from dogs into local wolves. Additionally, they identified some present-day wolves, specifically in Xinjiang in western China, that are symmetrically related to all dogs. The existence of wolves with such ancestry is unexpected under a dual ancestry model for dogs since one would expect any wolf to be closer to either eastern or western dogs. On this basis, they proposed that most of the gene flow underlying the asymmetries would have been from dogs into wolves (other than those in Xinjiang), rather than in the other direction.

Our results here show, to the contrary, that at least part of these asymmetries must reflect the Western wolf-related ancestry component present in western dogs (though it's also possible that recent, local gene flow has amplified these asymmetries). In addition, our findings on wolf population history offer a possible explanation for why the Xinjiang wolves are symmetrically related to all dogs. We modelled these wolves as intermediate between Late Pleistocene Siberian and European wolves (**Fig. 2b**). With the right proportions of ancestry related to eastern and western wolves, such an intermediate population could end up cancelling out the asymmetries to eastern and western dogs, and thereby appear to be symmetrically related to them. Our findings here thus imply that the ancestry of the Xinjiang wolves does not provide evidence against more than one wolf population contributing to the ancestry of dogs.

Multiple studies have observed that Pleistocene Siberian wolves have slightly higher affinity to dogs in Siberia and North America than to dogs in Europe, and proposed that this reflects admixture from wolves related to these Pleistocene Siberian wolves into Siberian dogs[3,15,47,54]. Our finding that there are two components of dog ancestry raises the possibility that these previous observations instead reflect how western wolf-related ancestry has diluted the Siberian wolf affinity in European and other western dogs. Using f_4-statistics, we confirmed that Pleistocene Siberian wolves are slightly closer to Siberian and Arctic dogs than to European dogs (**Fig. S22**). We also found, however, that the signal becomes stronger when using the African Basenji dog in place of the European dog, consistent with stronger dilution of eastern-related ancestry in African dogs.

Furthermore, we did not find any difference in the affinity of Pleistocene Siberian wolves to Siberian and Arctic dogs relative to the New Guinea singing dog. This demonstrates that the signal is not unique to Siberian and American dogs specifically, but is observed in east Eurasian dogs more broadly. A simple and likely explanation for these observations is that neither Siberian dogs nor New Guinea singing dogs have received post-domestication admixture from Siberian wolves, but that their stronger relative Siberian affinity reflects dilution in western dogs from the second, western-wolf related component of dog ancestry.

Frantz et al. analysed the genome of a 4,800-year old Neolithic dog from the site of Newgrange in Ireland, and suggested that this dog might have “a degree of ancestry from an ancient canid population that falls outside of the variation of modern dogs, but that is also different from modern wolves”[16]. This hypothesis was based on MSMC cross-coalescence curves in which the Newgrange dog appeared more divergent from East Asian dogs than what modern European dogs do, as well as PCA analyses in which the Newgrange dog was separated from modern dogs. Could these observations reflect the same dual ancestry signal we describe here, in which varying amounts of western Eurasian wolf-related ancestry push different western Eurasian dogs away from East Asian dogs to different degrees?

Based on the results we obtained for the Newgrange dog here, we do not think those observations reflect the same signal. We found that the Newgrange dog has less, not more, of the western Eurasian wolf-related ancestry component than what modern European dogs have (*qpAdm* estimates 6.2% for Newgrange and 14.9% for German Shepherd, **Fig. 4d**). Consistent with this, the Newgrange dog is among the most eastern-shifted dogs in the PCA based on relationships to pre-LGM wolves, more so than modern European dogs (**Fig. S20**). The second component of dog ancestry described here thus manifests itself in that modern European dogs have more of it than the Newgrange dog, and that modern European dogs are pushed away from eastern Eurasian dogs, rather than the other way around as in the observations made by Frantz et al[16]. These observations thus do not reflect the same underlying features of dog ancestry.

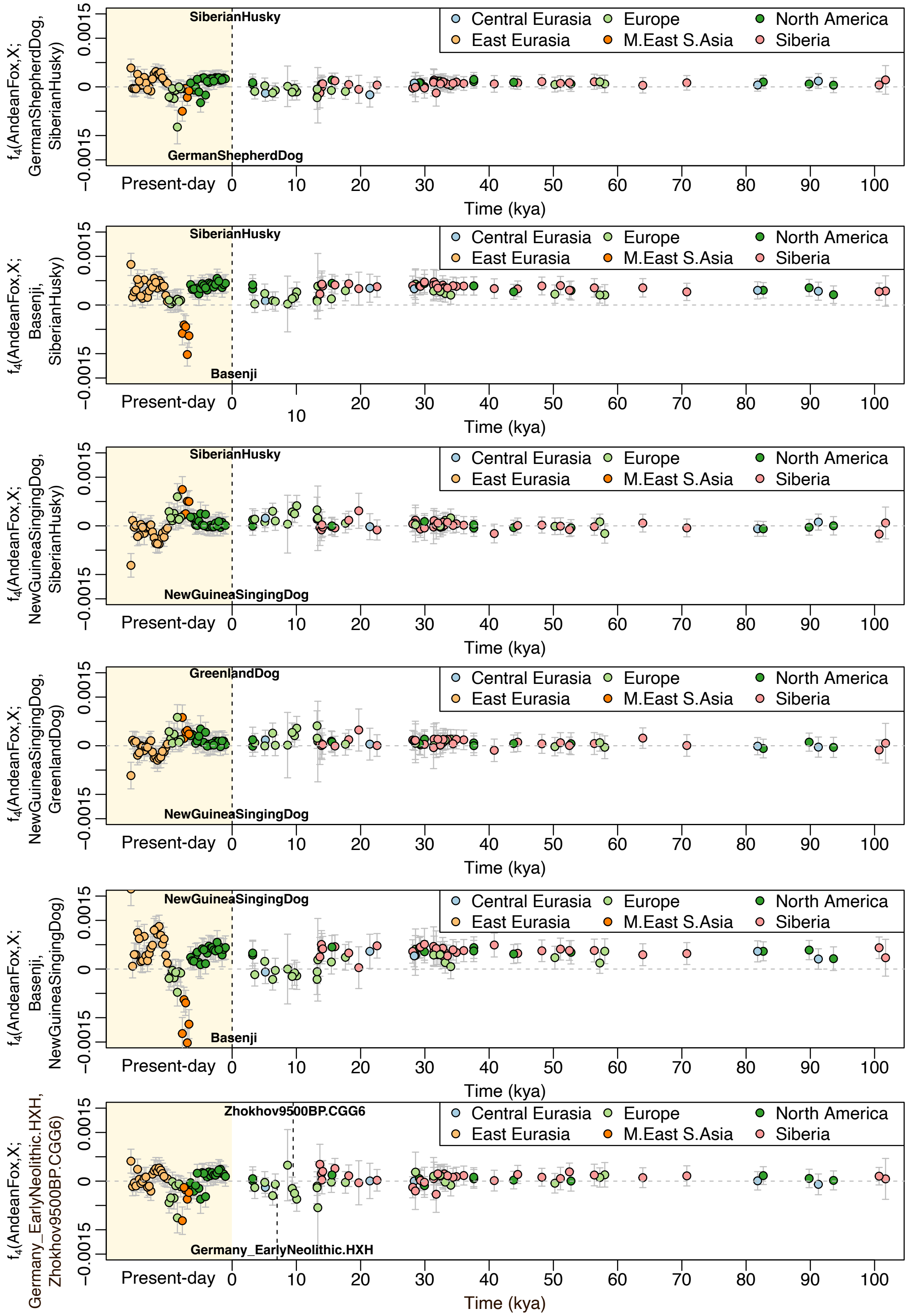

Fig. S22. f_4-statistics testing for asymmetrical relationships of wolves to pairs of dogs. Bars denote ±3 standard errors estimated from a block jackknife. The ages of the two dogs being contrasted are indicated with dashed lines, with positive values indicating affinity to the upper individual and negative values indicating affinity to the lower individual.

Reinterpreting relationships among early dog lineages

Accounting for the two distinct components of wolf ancestry that contributed to dogs will be necessary to accurately disentangle relationships among dogs themselves. We performed an admixture graph analysis of the relationships between selected dog genomes representing four major lineages. This followed a previous analysis[45] exactly, except that the American dog population used there was excluded, and that a present-day Syrian wolf was added (as the closest available proxy for the western-related dog progenitor). We thus used the following six populations: AndeanFox, NewGuineaSingingDog, Baikal (a pool of three ~7 ky-old dogs: C26, C27, OL4223), Karelia (OL4061), Levant (THRZ02), WolfSyria. We used the admixturegraph R package[55] to exhaustively test all 135,285 possible graphs that can be enumerated from these six populations with up to two admixture events. Each graph was fitted five times, and the one with the lowest "best_error" score was saved. Graphs that featured admixture in the history of the AndeanFox were ignored. We did not attempt graphs with more than six populations or two admixture events, as this would make for an extremely large set of graphs that could not be tested exhaustively.

Only a single graph fit all f_4-statistics ($|Z| < 3$). This graph models WolfSyria as an outgroup to NewGuineaSingingDog, Baikal and Karelia, and as contributing ancestry solely to the Levantine dog lineage. We refitted this graph using *qpGraph* (**Methods**), which also fit it with no outlier *f*-statistics. The *qpGraph* fit is displayed in **Fig. 4f**.

Two other graphs nearly fit the data, both of which imply WolfSyria-related ancestry in the Karelian dog lineage (via admixture from the Levant lineage, or directly from WolfSyria). Both display the same single minor (Z=3.01) outlier statistic: the graph is predicting a value of f_4*(AndeanFox,WolfSyria;Baikal,Karelia)* that is too large, implying an affinity of WolfSyria to Karelia over Baikal, which is not observed in the empirical data. To obtain more statistical power to distinguish between the top three graphs, we repeated the analysis with the ancient Levantine dog THRZ02 exchanged for the present-day African Basenji (which has a strong affinity to THRZ02[45]). THRZ02 has low sequencing coverage (0.1x) and thus lower power to reject graphs. Using the Basenji, the best-fitting graph still fits without outliers, but the two runner-up graphs can be more strongly rejected: they now have three outlier statistics, with the Z-score of the f_4*(AndeanFox,WolfSyria;Baikal,Karelia)* statistic going up to Z=6. The best-fitting graph thus appears to be a substantially better fit than any other possible graph within this six-population, two-admixture-events graph space.

To further characterise the fit of the best-fitting graph, we asked if this graph would fit if WolfSyria was exchanged for any other present-day wolf. Using *qpGraph*, the graph fits for 13 out of 17 other tested present-day wolves, including wolves both from western and eastern Eurasia (**Table S2**). However, for most of these wolves, the length of the ancestral drift branch that is shared by the present-day wolf and the lineage that contributes to THRZ02 is 0, meaning that the graph does not actually imply any shared ancestry between these two populations. By assigning this branch a length of 0, *qpGraph* is thus effectively causing the wolf-related

admixture to simply correspond to basal admixture, which is what is needed in THRZ02 to explain its relationships to the other dogs in the graph.

To further test the behaviour of different wolves in these *qpGraph* fits, we fitted a modified version of the graph that has admixture explicitly from a branch that is basal to dogs, but downstream of, and unrelated to, the wolf. For most of the tested wolves, this achieved an identical fit as the original graph. However, WolfSyria and other wolves from the Near East and South Asia (Wolf19India, Wolf20Iran, WolfSaudiArabia) behave differently in these analyses in two important ways. Firstly, in the graphs with admixture coming from these wolves, the edge length is inferred to be non-zero, implying actual shared ancestry between these wolves and THRZ02. Secondly, the graphs with just basal dog admixture do not fit for these wolves, as affinity between the given wolf and THRZ02 is not accounted for. These additional results thus support the conclusion that our best-fitting graph does in fact reflect shared ancestry between THRZ02 and present-day Near Eastern wolves specifically, rather than some more general phenomenon that manifests with any present-day wolf. In summary, these admixture graph results are consistent with the *qpWave*/*qpAdm* results relating to dog ancestry. Specifically, New Guinea singing dog, Baikal and Karelia are modelled as 100% “Eastern Dog Progenitor” ancestry and 0% “Western Dog Progenitor” ancestry (**Fig. 4c,d,e**).

In the previous admixture graph analysis[45], the only graph that fit in a similar exhaustive search (not including any wolves, but including an American dog population) was one in which the Karelian lineage received ~1/3rd of its ancestry through admixture from the Levantine (THRZ02) lineage. Assuming THRZ02 has approximately 50% western dog progenitor ancestry (**Fig. 4d**), that previous graph would imply that the Karelian dog would have one third as much, i.e. ~1/6 ≈ 16.7% western dog progenitor ancestry, which does not seem compatible with our *qpWave*/*qpAdm* results. The single-source Zhokhov dog model is not rejected for the Karelian dog (**Fig. 4d**), and in the two-source Zhokhov+WolfSyria model, the inferred ancestry proportion assigned to WolfSyria is only 2.8% (SE: 3.7%). While we cannot rule out that the Karelian dog could have a few percent western dog progenitor ancestry, undetectable at the current resolution of our data, a higher proportion seems unlikely.

This result thus suggests that the previous graph is not correct in featuring Levantine gene flow into the Karelian dog. Our new admixture graph analysis shows that alternative solutions exist, one of which instead features Karelian-related admixture going into the Levantine lineage. While available dog genomes might not impose strong constraints on the directionality of admixture between early dog lineages, introducing additional constraints through the relationships to wolf genomes can thus help distinguish between different models of dog population history. In principle, similar graph solutions to the one identified here could have been identified in the previous analysis that did not include a Near Eastern wolf[45], just with basal admixture into THRZ02 instead of Near Eastern wolf-related admixture. However, the absence of any graphs like that fitting the data suggests that a third admixture event would be needed in such topologies. Since the ancient American dog population cannot be fit as a clade with any of the other dogs in the analysis, including this population tends to require one admixture event to resolve its placement. Here, by excluding the American population, we free up one admixture

event, and thus are able to find alternative solutions. Interestingly, the topology of the graph identified here (**Fig. 4f**) has the eastern dog progenitor component of the Levantine dog coming not from a direct sister branch of the Karelian dog, but from a branch that is ancestral to Karelia and part of Baikal ancestry. Additional ancient dog genomes from western Eurasia are necessary to further disentangle these early diversification events of the eastern dog progenitor ancestry as it spread west.

Table S2. Dog admixture graph fits involving different present-day wolves. Different present-day wolves were used in the place of WolfSyria in the admixture graph in **Fig. 4f**. The Levantine dog sample THRZ02 received admixture either from a lineage related to the tested wolf, as in **Fig. 4f** ("Wolf admixture" columns), or from a branch that is basal to dogs but downstream of and unrelated to the wolf ("Basal dog admixture") columns. Edge lengths are in units of F_{ST} ×1000.

Tested wolf	Wolf admixture, nr outliers	Wolf admixture, worst outlier (Z)	Wolf admixture, shared edge length	Basal dog admixture, nr outliers	Basal dog admixture, worst outlier (Z)
**Wolf01Altai**	0	1.28	1	0	1.35
**Wolf02Chukotka**	0	-2.51	0	0	-2.51
**Wolf03Bryansk**	0	-2.06	0	0	-2.06
**Wolf06Croatia**	0	-0.89	0	0	-0.86
**Wolf07Israel**	3	-7.84	3	5	-7.84
**Wolf19India**	0	-2.66	3	3	4.09
**Wolf20Iran**	0	-1.94	3	3	4.13
**Wolf21Italy**	0	-0.98	1	0	-0.98
**Wolf24Portugal**	0	-2.15	0	0	-2.15
**Wolf27Spain**	2	-4.39	0	2	-4.39
**Wolf33Xinjiang**	2	4.34	0	2	4.34
**Wolf34Shanxi**	5	-6.45	0	5	-6.45
**Wolf35Xinjiang**	2	3.97	0	2	3.97
**Wolf36Xinjiang**	0	-2.68	0	0	-2.68
**Wolf39Iberia**	0	-1.99	0	0	-1.99
**WolfSaudiArabia**	0	-2.66	10	17	10.26
**WolfSyria**	0	-1.63	5	3	4.58
**Wolf21-M-02-15Scandinavia**	0	-2.7	0	0	-2.7

Radiocarbon dating of Near Eastern dogs

We obtained radiocarbon dates for four ancient dogs from Israel, for which whole-genome sequencing data has been published previously[45]. The dates were generated by the Oxford Radiocarbon Accelerator Unit, and calibrated using IntCal20[56] in OxCal v4.4[57] (**Table S3**). For three of the dogs, the radiocarbon dates agree well with the periods inferred from their archaeological contexts. The sample UZAA02, however, had been inferred to likely be from a Byzantine context, but the radiocarbon date showed that the specimen is more recent and instead from the Islamic period, similar in age to the sample UZAA01.

Of direct interest within the context of the current study, the date for the Neolithic dog sample THRZ02 is consistent with its late pottery Neolithic archaeological context. With a mean date of 7171 cal BP, this sample thus provides the currently earliest constraint on the appearance of western dog progenitor ancestry.

Table S3. Newly reported radiocarbon dates for four Near Eastern dog samples.

Date ID	Date uncal.	SE	Site	Sample ID	Cal BP mean	Cal BP from	Cal BP to
OxA-40938	2492	20	Tel Gezer	TGEZ06	2606	2720	2491
OxA-40605	6268	27	Tel Hreiz	THRZ02	7171	7265	7076
OxA-40606	987	20	Uza	UZAA01	876	955	797
OxA-40607	979	20	Uza	UZAA02	863	929	796

Supplementary References

1. Ho, S. Y. W. *et al.* Bayesian estimation of substitution rates from ancient DNA sequences with low information content. *Syst. Biol.* **60**, 366–375 (2011).
2. Suchard, M. A. *et al.* Bayesian phylogenetic and phylodynamic data integration using BEAST 1.10. *Virus Evolution* vol. 4 (2018).
3. Skoglund, P., Ersmark, E., Palkopoulou, E. & Dalén, L. Ancient wolf genome reveals an early divergence of domestic dog ancestors and admixture into high-latitude breeds. *Curr. Biol.* **25**, 1515–1519 (2015).
4. Loog, L. *et al.* Ancient DNA suggests modern wolves trace their origin to a Late Pleistocene expansion from Beringia. *Mol. Ecol.* **29**, 1596–1610 (2020).
5. Ramos Madrigal, J. *et al.* Genomes of Pleistocene Siberian Wolves Uncover Multiple Extinct Wolf Lineages. *Curr. Biol.* **31**, 1–9 (2021).
6. Gopalakrishnan, S. *et al.* Interspecific Gene Flow Shaped the Evolution of the Genus *Canis*. *Curr. Biol.* **28**, 3441–3449.e5 (2018).
7. Wang, M.-S. *et al.* Ancient Hybridization with an Unknown Population Facilitated High-Altitude Adaptation of Canids. *Mol. Biol. Evol.* **37**, 2616–2629 (2020).
8. Werhahn, G. *et al.* Phylogenetic evidence for the ancient Himalayan wolf: towards a clarification of its taxonomic status based on genetic sampling from western Nepal. *R Soc Open Sci* **4**, 170186 (2017).
9. Sharma, D. K., Maldonado, J. E., Jhala, Y. V. & Fleischer, R. C. Ancient wolf lineages in India. *Proc. Biol. Sci.* **271 Suppl 3**, S1–4 (2004).
10. Patterson, N. *et al.* Ancient admixture in human history. *Genetics* **192**, 1065–1093 (2012).
11. Lazaridis, I., Belfer-Cohen, A., Mallick, S. & Patterson, N. Paleolithic DNA from the Caucasus reveals core of West Eurasian ancestry. *bioRxiv* (2018).
12. Aggarwal, R. K., Kivisild, T., Ramadevi, J. & Singh, L. Mitochondrial DNA coding region sequences support the phylogenetic distinction of two Indian wolf species. *J. Zoolog. Syst.*

Evol. Res. **45**, 163–172 (2007).

13. vonHoldt, B. M. *et al.* Whole-genome sequence analysis shows that two endemic species of North American wolf are admixtures of the coyote and gray wolf. *Sci Adv* **2**, e1501714 (2016).
14. Sinding, M.-H. S. *et al.* Population genomics of grey wolves and wolf-like canids in North America. *PLoS Genet.* **14**, e1007745 (2018).
15. Sinding, M.-H. S. *et al.* Arctic-adapted dogs emerged at the Pleistocene–Holocene transition. *Science* **368**, 1495–1499 (2020).
16. Frantz, L. A. F. *et al.* Genomic and archaeological evidence suggest a dual origin of domestic dogs. *Science* **352**, 1228–1231 (2016).
17. Bai, B. *et al.* DoGSD: the dog and wolf genome SNP database. *Nucleic Acids Res.* **43**, D777–83 (2015).
18. Poplin, R. *et al.* Scaling accurate genetic variant discovery to tens of thousands of samples. *bioRxiv* 201178 (2018) doi:10.1101/201178.
19. Derrien, T. *et al.* Fast computation and applications of genome mappability. *PLoS One* **7**, e30377 (2012).
20. Wang, K., Mathieson, I., O'Connell, J. & Schiffels, S. Tracking human population structure through time from whole genome sequences. *PLoS Genet.* **16**, e1008552 (2020).
21. Koch, E. *et al.* De novo mutation rate estimation in wolves of known pedigree. *Mol. Biol. Evol.* (2019) doi:10.1093/molbev/msz159.
22. Mech, L. D., Barber-Meyer, S. M. & Erb, J. Wolf (*Canis lupus*) Generation Time and Proportion of Current Breeding Females by Age. *PLoS One* **11**, e0156682 (2016).
23. Bergström, A. *et al.* A Neolithic expansion, but strong genetic structure, in the independent history of New Guinea. *Science* **357**, 1160–1163 (2017).
24. 1000 Genomes Project Consortium. A global reference for human genetic variation. *Nature* **526**, 68–74 (2015).

25. Fan, Z. *et al.* Worldwide patterns of genomic variation and admixture in gray wolves. *Genome Research* vol. 26 163–173 (2016).
26. Freedman, A. H. *et al.* Genome sequencing highlights the dynamic early history of dogs. *PLoS Genet.* **10**, e1004016 (2014).
27. Zhang, W. *et al.* Hypoxia adaptations in the grey wolf (*Canis lupus chanco*) from Qinghai-Tibet Plateau. *PLoS Genet.* **10**, e1004466 (2014).
28. Li, H. & Durbin, R. Inference of human population history from individual whole-genome sequences. *Nature* **475**, 493–496 (2011).
29. Hudson, R. R. Generating samples under a Wright–Fisher neutral model of genetic variation. *Bioinformatics* **18**, 337–338 (2002).
30. Campbell, C. L., Bhérer, C., Morrow, B. E., Boyko, A. R. & Auton, A. A Pedigree-Based Map of Recombination in the Domestic Dog Genome. *G3* **6**, 3517–3524 (2016).
31. Wong, A. K. *et al.* A comprehensive linkage map of the dog genome. *Genetics* **184**, 595–605 (2010).
32. Devlin, B. & Roeder, K. Genomic control for association studies. *Biometrics* **55**, 997–1004 (1999).
33. Plassais, J. *et al.* Whole genome sequencing of canids reveals genomic regions under selection and variants influencing morphology. *Nat. Commun.* **10**, 1489 (2019).
34. Delaneau, O., Zagury, J. F., Robinson, M. R., Marchini, J. L. & Dermitzakis, E. T. Accurate, scalable and integrative haplotype estimation. *Nat. Commun.* **10**, 24–29 (2019).
35. Speidel, L., Forest, M., Shi, S. & Myers, S. R. A method for genome-wide genealogy estimation for thousands of samples. *Nat. Genet.* **51**, 1321–1329 (2019).
36. Auton, A. *et al.* Genetic recombination is targeted towards gene promoter regions in dogs. *PLoS Genet.* **9**, e1003984 (2013).
37. Speidel, L. et al. Inferring Population Histories for Ancient Genomes Using Genome-Wide Genealogies. *Molecular Biology and Evolution* **38**, 3497–3511 (2021).

38. Skoglund, P., Ersmark, E., Palkopoulou, E. & Dalén, L. Ancient wolf genome reveals an early divergence of domestic dog ancestors and admixture into high-latitude breeds. *Curr. Biol.* **25**, 1515–1519 (2015).

39. Stern, A. J., Wilton, P. R. & Nielsen, R. An approximate full-likelihood method for inferring selection and allele frequency trajectories from DNA sequence data. *PLoS Genet.* **15**, e1008384 (2019).

40. Candille, S. I. *et al.* A β-defensin mutation causes black coat color in domestic dogs. *Science* **318**, 1418–1423 (2007).

41. Anderson, T. M. *et al.* Molecular and evolutionary history of melanism in North American gray wolves. *Science* **323**, 1339–1343 (2009).

42. Ollivier, M. *et al.* Evidence of coat color variation sheds new light on ancient canids. *PLoS One* **8**, e75110 (2013).

43. Schweizer, R. M. *et al.* Natural Selection and Origin of a Melanistic Allele in North American Gray Wolves. *Mol. Biol. Evol.* **35**, 1190–1209 (2018).

44. Thorvaldsdóttir, H., Robinson, J. T. & Mesirov, J. P. Integrative Genomics Viewer (IGV): high-performance genomics data visualization and exploration. *Brief. Bioinform.* **14**, 178–192 (2013).

45. Bergström, A. *et al.* Origins and genetic legacy of prehistoric dogs. *Science* **370**, 557–564 (2020).

46. Reich, D. *et al.* Reconstructing Native American population history. *Nature* **488**, 370–374 (2012).

47. Pilot, M. *et al.* Global Phylogeographic and Admixture Patterns in Grey Wolves and Genetic Legacy of An Ancient Siberian Lineage. *Sci. Rep.* **9**, 17328 (2019).

48. Vaysse, A. *et al.* Identification of genomic regions associated with phenotypic variation between dog breeds using selection mapping. *PLoS Genet.* **7**, e1002316 (2011).

49. Stronen, A. V. *et al.* Genome-wide analyses suggest parallel selection for universal traits

may eclipse local environmental selection in a highly mobile carnivore. *Ecol. Evol.* **5**, 4410–4425 (2015).

50. Cronin, M. A., Cánovas, A., Bannasch, D. L., Oberbauer, A. M. & Medrano, J. F. Single nucleotide polymorphism (SNP) variation of wolves (*Canis lupus*) in Southeast Alaska and comparison with wolves, dogs, and coyotes in North America. *J. Hered.* **106**, 26–36 (2015).
51. Fitak, R. R., Rinkevich, S. E. & Culver, M. Genome-Wide Analysis of SNPs Is Consistent with No Domestic Dog Ancestry in the Endangered Mexican Wolf (*Canis lupus baileyi*). *J. Hered.* **109**, 372–383 (2018).
52. Pilot, M. *et al.* On the origin of mongrels: evolutionary history of free-breeding dogs in Eurasia. *Proc. Biol. Sci.* **282**, 20152189 (2015).
53. Patterson, N., Price, A. L. & Reich, D. Population structure and eigenanalysis. *PLoS Genet.* **2**, e190 (2006).
54. Ramos-Madrigal, J. *et al.* Genomes of Pleistocene Siberian Wolves Uncover Multiple Extinct Wolf Lineages. *Curr. Biol.* (2020) doi:10.1016/j.cub.2020.10.002.
55. Leppälä, K., Nielsen, S. V. & Mailund, T. admixturegraph: an R package for admixture graph manipulation and fitting. *Bioinformatics* **33**, 1738–1740 (2017).
56. Reimer, P. J. *et al.* The IntCal20 Northern Hemisphere Radiocarbon Age Calibration Curve (0–55 cal kBP). *Radiocarbon* **62**, 725–757 (2020).
57. Ramsey, C. B. Bayesian Analysis of Radiocarbon Dates. *Radiocarbon* **51**, 337–360 (2009).
