# Supplementary material for: Grey wolf genomic history reveals a dual ancestry of dogs
Source: Nature. 2022 Jun 29;607(7918):313–20. doi: 10.1038/s41586-022-04824-9 (PMC9279150; doi:10.1038/s41586-022-04824-9)
Supplement: Supplementary file 2 — Reporting Summary [file 41586_2022_4824_MOESM2_ESM.pdf]

nature portfolio

Corresponding author(s): Anders Bergstrom, Pontus Skoglund

Last updated by author(s): Mar 3, 2022

# Reporting Summary

Nature Portfolio wishes to improve the reproducibility of the work that we publish. This form provides structure for consistency and transparency in reporting. For further information on Nature Portfolio policies, see our Editorial Policies and the Editorial Policy Checklist.

## Statistics

For all statistical analyses, confirm that the following items are present in the figure legend, table legend, main text, or Methods section.

| n/a | Confirmed | |
|---|---|---|
| ☒ | ☐ | The exact sample size (*n*) for each experimental group/condition, given as a discrete number and unit of measurement |
| ☒ | ☐ | A statement on whether measurements were taken from distinct samples or whether the same sample was measured repeatedly |
| ☐ | ☒ | The statistical test(s) used AND whether they are one- or two-sided<br>*Only common tests should be described solely by name; describe more complex techniques in the Methods section.* |
| ☐ | ☒ | A description of all covariates tested |
| ☐ | ☒ | A description of any assumptions or corrections, such as tests of normality and adjustment for multiple comparisons |
| ☐ | ☒ | A full description of the statistical parameters including central tendency (e.g. means) or other basic estimates (e.g. regression coefficient) AND variation (e.g. standard deviation) or associated estimates of uncertainty (e.g. confidence intervals) |
| ☐ | ☒ | For null hypothesis testing, the test statistic (e.g. *F*, *t*, *r*) with confidence intervals, effect sizes, degrees of freedom and *P* value noted<br>*Give P values as exact values whenever suitable.* |
| ☐ | ☒ | For Bayesian analysis, information on the choice of priors and Markov chain Monte Carlo settings |
| ☒ | ☐ | For hierarchical and complex designs, identification of the appropriate level for tests and full reporting of outcomes |
| ☐ | ☒ | Estimates of effect sizes (e.g. Cohen's *d*, Pearson's *r*), indicating how they were calculated |

*Our web collection on statistics for biologists contains articles on many of the points above.*

## Software and code

Policy information about availability of computer code

| | |
|---|---|
| Data collection | No software was used for data collection. |
| Data analysis | SeqPrep, BWA aln v.0.7.17, BWA mem v0.7.15, PMDtools v0.60, Picard Tools v2.21.4, GATK HaplotypeCaller v3.6, bcftools v.1.8, htsbox pileup r345, samtools v1.9, Geneious v9.0.5, Clustal Omega v1.2.4, BEAST v1.10.1, JModelTest2 v2.1.10, ADMIXTOOLS v5.0, R package pcaMethods v1.74.0, EIGENSOFT 7.2.1, gem-mappability v1.315, MSMC2 v2.1.2, PLINK v1.90b5.2, ms, SHAPEIT4 v4.2.1, Relate v1.1.8, CLUES, Integrative Genomics Viewer (IGV) 2.4.14, R package mapdata 2.3.0, OxCal v4.4 |

For manuscripts utilizing custom algorithms or software that are central to the research but not yet described in published literature, software must be made available to editors and reviewers. We strongly encourage code deposition in a community repository (e.g. GitHub). See the Nature Portfolio guidelines for submitting code & software for further information.

## Data

Policy information about availability of data

All manuscripts must include a data availability statement. This statement should provide the following information, where applicable:

- Accession codes, unique identifiers, or web links for publicly available datasets
- A description of any restrictions on data availability
- For clinical datasets or third party data, please ensure that the statement adheres to our policy

The generated DNA sequencing data is available in the European Nucleotide Archive (ENA) under study accession PRJEB42199. Previously published genomic data analysed here is available under accession numbers PRJNA448733, PRJCA000335, PRJEB20635, PRJNA496590, PRJNA494815, PRJEB7788, PRJEB13070, PRJNA319283, PRJEB22026, PRJNA608847, PRJEB38079, PRJEB39580 and PRJEB41490, with individual genomes used listed in Supplementary Data 2. The

canFam3.1 reference genome is available under NCBI assembly accession GCF_000002285.3.

# Field-specific reporting

Please select the one below that is the best fit for your research. If you are not sure, read the appropriate sections before making your selection.

☒ Life sciences ☐ Behavioural & social sciences ☐ Ecological, evolutionary & environmental sciences

For a reference copy of the document with all sections, see nature.com/documents/nr-reporting-summary-flat.pdf

# Life sciences study design

All studies must disclose on these points even when the disclosure is negative.

| | |
|---|---|
| Sample size | No sample size calculations were made. This is a genomic study of paleontological material, where sample size was shaped by availability of material (ancient wolf remains) and their DNA preservation upon screening. This is the largest ancient genomic study of Pleistocene genomes to date. The sample size of over 70 ancient wolves and hundreds of canids in the published literature provides substantial statistical power, notably due to the evolutionary variance being accounted for by analysis of the entire genome - comprising tens of hundreds of thousands of independent loci. |
| Data exclusions | All genome sequencing data collected for this study was analyzed. Certain population genetic analyses were restricted to subsets of genomes (e.g. those meeting thresholds of sequencing coverage or other measures of data quality) as detailed in the Methods section and Supplementary Information. |
| Replication | This was a retrospective study of an evolutionary history that has occurred only once, and it was not possible to observe independent replicates of this history. |
| Randomization | This was a retrospective study of an evolutionary history that has occurred only once, and it was not possible to randomize the application of different past processes to the analyzed genome sequences. |
| Blinding | Blinding was not applicable to this study, as each genome sequence had to be associated with its spatial and temporal metadata in order to draw conclusions. |

# Reporting for specific materials, systems and methods

We require information from authors about some types of materials, experimental systems and methods used in many studies. Here, indicate whether each material, system or method listed is relevant to your study. If you are not sure if a list item applies to your research, read the appropriate section before selecting a response.

## Materials & experimental systems

| n/a | Involved in the study | |
|---|---|---|
| ☒ | ☐ | Antibodies |
| ☒ | ☐ | Eukaryotic cell lines |
| ☐ | ☒ | Palaeontology and archaeology |
| ☒ | ☐ | Animals and other organisms |
| ☒ | ☐ | Human research participants |
| ☒ | ☐ | Clinical data |
| ☒ | ☐ | Dual use research of concern |

## Methods

| n/a | Involved in the study | |
|---|---|---|
| ☒ | ☐ | ChIP-seq |
| ☒ | ☐ | Flow cytometry |
| ☒ | ☐ | MRI-based neuroimaging |

## Palaeontology and Archaeology

| | |
|---|---|
| Specimen provenance | The metadata for the 67 wolf remains from which novel genome sequencing data is reported is described in the table in Supplementary Data 1. For each specimen, this table lists the name and geographical coordinates of the site of excavation or collection, the steward institution that provided access to and is responsible for the long-term storage of the specimen, the excavation or museum collection identifier if applicable, and what skeletal element was sampled for the purpose of DNA extraction. As no new excavations were performed in this study, no excavation permits were necessary. Sampling for DNA extraction was performed with the permission of the specimen stewards, all of which are listed in Supplementary Data 1, and most of which are authors on the paper. |
| Specimen deposition | The metadata table in Supplementary Data 1 lists, for each of the 67 wolf remains from which novel genome sequencing data is reported, the steward institution that provided access to and is responsible for the long-term storage of the specimen, and the excavation or museum collection identifier if applicable. Requests for access to the specimens should be directed to these host institutions. |

| | |
|---|---|
| Dating methods | New radiocarbon dates were obtained from the Oxford Radiocarbon Accelerator Unit and calibrated using the IntCal20 calibration curve in the OxCal v4.4 software. We refer to the dating laboratory for details on their experimental protocol. |

☒ Tick this box to confirm that the raw and calibrated dates are available in the paper or in Supplementary Information.

| | |
|---|---|
| Ethics oversight | No ethical oversight was required as this study comprises only zooarchaeological material, previously collected and curated by individual institutions and researchers following local regulations. Sampling for DNA was performed aiming to minimize the destructive impact on the zooarchaeological material. |

Note that full information on the approval of the study protocol must also be provided in the manuscript.
